# Visible light-induced photocatalytic C–H ethoxycarbonylmethylation of imidazoheterocycles with ethyl diazoacetate†

**DOI:** 10.1039/d0ra05795a

**Published:** 2020-07-28

**Authors:** Suvam Bhattacharjee, Sudip Laru, Sadhanendu Samanta, Mukta Singsardar, Alakananda Hajra

**Affiliations:** Department of Chemistry, Visva-Bharati (A Central University) Santiniketan 731235 West Bengal India alakananda.hajra@visva-bharati.ac.in http://www.visvabharati.ac.in/AlakanandaHajra.html

## Abstract

A visible light-mediated regioselective C3-ethoxycarbonylmethylation of imidazopyridines with ethyl diazoacetate (EDA) was achieved under mild reaction conditions. In contrast to the carbene precursors from α-diazoester a first C3-ethoxycarbonylmethylation of imidazopyridines *via* a radical intermediate has been established. The present methodology provides a concise route to access pharmacologically useful esters with wide functional group tolerance in high yields.

## Abstract 2

A visible-light-promoted regioselective ethoxycarbonylmethylation of imidazoheterocycles has been developed using an α-diazoester *via* a radical pathway.

## Graphical abstract

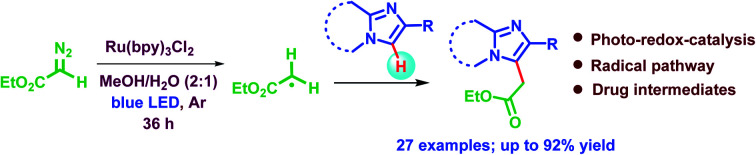

Ester groups are a most powerful and versatile synthetic building block in natural products as well as in organic synthesis since they can be derivatized to diverse functional groups such as carbonyl, hydroxymethyl, and amide, *etc.*^1^ Beside the traditional methods of using xanthates for ethoxycarbonylmethylation,^2^ the introduction of diazoesters represents a very important strategy for the synthesis of acetate derivatives.^3^ Generally, thermal,^4*a*^ photoinduced,^4*b*^ or metal catalyzed^4*c*^ reactions of diazo compounds produce reactive carbene precursors which further undergo an addition reaction, insertion reaction, cyclopropanation, Wolff rearrangement, *etc.* (Scheme 1a).^4^ In contrast to the carbene precursors from α-diazoester, a new methodology is always highly desirable especially with a significantly different mode of activation under a distinct mechanism.^5^ In this context, a new strategy for installation of alkyl radicals from diazo compounds under photoredox-catalysis is very important.^6^ Recently, visible light-mediated photoredox catalyzed direct C–H bond functionalization reactions have come to the forefront in synthetic organic chemistry^7^ where Ru(ii)-polypyridyl complexes have been extensively used as photoredox-catalysts due to their unique photo-physical properties.^8^

**Scheme 1:**
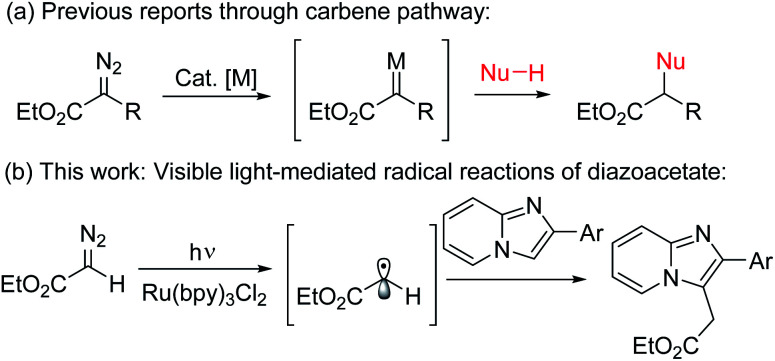
Visible light-mediated ethoxycarbonylmethylation. (a) Previous reports through carbene pathway. (b) This work: visible-light mediated radical reaction of diazoacetate with imidazopyridine.

Imidazo[1,2-*a*]pyridines, an important class of nitrogen-based privileged structural motifs widely found in a variety of natural products and alkaloids.^9^ In pharmaceutical chemistry, it possesses large number of biological activities like antitumor, antibacterial, antifungal, antipyretic, analgesic, antiinflammatory, *etc.*^10*a*^ It is also important in material sciences due to its unique excited state intramolecular proton transfer phenomena (ESIPT).^10*b*^ In particular, ethoxycarbonylmethylated imidazopyridines are the core structure of several marketed drugs such as alpidem, zolpidem, saripidem, *etc.*^11^ Therefore, practical methodologies for the synthesis of alkylated ester substituted imidazoheterocycles are highly desirable and will be of great interest to the synthetic chemists.^11^ However, to the best of our knowledge there is no such method for the direct photo-initiated ethoxycarbonylmethylation reaction of imidazoheterocycles with diazo compounds through distinct radical pathway.^11–13^ As part of our ongoing research on photoredox-catalyzed C–H functionalization reactions,^13^ herein we describe an environmentally benign visible light-promoted ethoxycarbonylmethylation for the synthesis of alkyl substituted imidazopyridines by the coupling between imidazopyridines and ethyl diazoacetate using Ru(bpy)_3_Cl_2_ as photoredox-catalyst under argon atmosphere at room temperature (Scheme 1b).

To begin, the reaction was carried out using 2-phenylimidazo[1,2-*a*]pyridine (1a) and ethyl diazoacetate (EDA) (2) (2.0 equiv.) in presence of 0.2 mol% of Ru(bpy)_3_Cl_2_ as a photoredox-catalyst under the irradiation of 34 W blue LED lamp in methanol at room temperature. To our delight, 49% yield of the ethyl 2-(2-phenylimidazo[1,2-*a*]pyridin-3-yl)acetate (3a) was obtained after 36 h under argon atmosphere. However, no desired product was formed in aerobic conditions (Table 1, entry 1). Next, we screened the effect of different solvents such as EtOH, DCM, MeCN, and toluene but these were not suitable like MeOH (Table 1, entries 2–5). Interestingly, the binary solvent mixture (MeOH and H_2_O, 1 : 1) was found to be effective for the formation of alkylated product in 76% yield (Table 1, entry 6). The yield of the reaction was increased using a binary mixture of MeOH : H_2_O (2 : 1) (Table 1, entry 7). However, only a trace amount of product was detected with the mixture of (1 : 2) MeOH : H_2_O and no product was observed in H_2_O (Table 1, entries 8 and 9). Instead of MeOH : H_2_O (2 : 1) by using EtOH : H_2_O (2 : 1) desired product (3a) was obtained in 68% yield after 36 h (Table 1 entry 10). The reaction did not proceed at all in presence of other classical photoredox-catalysts like Ir(ppy)_3_, rose bengal, eosin Y, eosin B, and rhodamine B (Table 1, entries 11–15). Moreover, no product was formed in absence of photoredox-catalyst as well as light source (Table 1, entries 16 and 17) which confirm that photo-catalyst and light source are essential for this transformation. We also performed the reaction in thermal condition at 60 °C (Table 1, entry 18). The desired product (3a) was not formed which indicated that the reaction probably go through the charge transfer/EDA-type complex not *via* carbene pathway. The yield of the reaction decreased when the amount of ethyl diazoacetate was either lowered or increased (Table 1, entry 19). In addition, 56% yield of the desired product was obtained under the irradiation of 20 W blue LED and no alkylated product was observed using 30 W white LED (Table 1, entry 20). From this experiment, we confirmed that the probable reaction pathway *via* charge-transfer/EDA-type complex absorbs wave length only from the emitted blue LED light not from the emitted 30 W white LED. Finally, optimized yield (89%) was achieved using 0.2 mol% Ru(bpy)_3_Cl_2_ and 2.0 equiv. ethyl diazoacetate in 2.0 mL mixture of MeOH : H_2_O (2 : 1) with the irradiation of 34 W blue LED for 36 h at room temperature under argon atmosphere (Table 1, entry 7).

**Table 1:** Optimization of the reaction conditions^a^

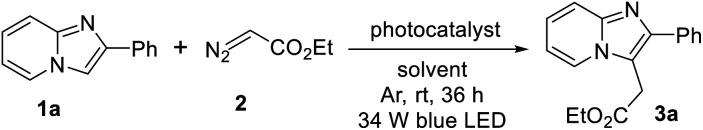			
Entry	Photocatalyst (0.2 mol%)	Solvent	Yield (%)
1	Ru(bpy)_3_Cl_2_	MeOH	49, NR^b^
2	Ru(bpy)_3_Cl_2_	EtOH	21
3	Ru(bpy)_3_Cl_2_	DCM	43
4	Ru(bpy)_3_Cl_2_	MeCN	47
5	Ru(bpy)_3_Cl_2_	Toluene	NR
6	Ru(bpy)_3_Cl_2_	MeOH : H_2_O (1 : 1)	76
7	Ru(bpy)_3_Cl_2_	MeOH : H_2_O (2 : 1)	89
8	Ru(bpy)_3_Cl_2_	MeOH : H_2_O (1 : 2)	Trace
9	Ru(bpy)_3_Cl_2_	H_2_O	NR
10	Ru(bpy)_3_Cl_2_	EtOH : H_2_O (2 : 1)	68
11	Ir(ppy)_3_	MeOH : H_2_O (2 : 1)	Trace
12	Rose bengal	MeOH : H_2_O (2 : 1)	NR
13	Eosin Y	MeOH : H_2_O (2 : 1)	NR
14	Eosin B	MeOH : H_2_O (2 : 1)	NR
15	Rhodamine B	MeOH : H_2_O (2 : 1)	NR
16	—	MeOH : H_2_O (2 : 1)	NR^c^
17	Ru(bpy)_3_Cl_2_	MeOH : H_2_O (2 : 1)	NR^d^
18	Ru(bpy)_3_Cl_2_	MeOH : H_2_O (2 : 1)	NR^e^
19	Ru(bpy)_3_Cl_2_	MeOH : H_2_O (2 : 1)	45^f^, 84^g^
20	Ru(bpy)_3_Cl_2_	MeOH : H_2_O (2 : 1)	56^h^, NR^i^

a Reaction conditions: all reactions were carried out with 0.25 mmol of 1a, 0.5 mmol of 2, and 0.2 mol% of photocatalyst in 2.0 mL of solvent for 36 h at room temperature under argon atmosphere and irradiation with 34 W blue LED. NR = no reaction.

b In aerobic condition.

c Absence of Ru(bpy)_3_Cl_2_.

d Without light source at rt.

e Reaction at 60 °C without light scource.

f 1.5 equiv. EDA.

g 2.5 equiv. EDA.

h Irradiation with 20 W blue LED.

i 30 W white LED.

With the optimized reaction conditions in hand we explored the substrate scope of this methodology with various substituted imidazopyridines. At first we checked the effect of various substituents on the pyridine ring of imidazopyridine derivatives and the results are summarized in Scheme 2. Imidazopyridine containing various electron-donating groups (8-Me and 7-OMe) efficiently reacted with ethyl diazoacetate to provide the desired products in good yields (3b and 3c). It is important to note that substrates bearing electron-withdrawing groups did not afford alkylated products under the optimized reaction condition. However, addition of 10 mol% *N*,*N*-dimethyl-*m*-toluidine as a redox-active additive gave a satisfactory yield of the desired products (see ESI†). Therefore in modified reaction conditions, halogens containing imidazopyridines like –F, –Br, –I and strong electron-withdrawing group such as –CF_3_, –CN containing substrates were successfully afforded the desired products in 67–90% yields (3d–3h). Next, we examined the substrate scope with various substitutions in phenyl part of imidazo[1,2-*a*]pyridine. Imidazopyridine with electron-releasing (4-Me, and 4-OMe), halogens (4-F, 4-Cl, and 3-Br) and strong electron-withdrawing group (4-CF_3_, and 4-CN) containing substrates produced the desired products with good to excellent yields (3i–3p). 2-Hydroxy substituted imidazo[1,2-*a*]pyridine was also worked well in this transformation (3k). In addition, disubstituted imdazopyridines with different substituents at both the phenyl and pyridine ring underwent the reaction very smoothly (3q, and 3r). Heteroaryl and naphthyl substituted imidazopyridines produced the desired products without any difficulties (3s–3u). However, unsubstituted imidazo[1,2-*a*]pyridine did not produce the desired product in this protocol. To highlight the applicability of the present protocol a gram-scale reaction was carried out taking 1a in 6.0 mmol scale under normal laboratory setup. The yield of ethyl 2-(2-phenylimidazo[1,2-*a*]pyridin-3-yl)acetate (3a) was slightly diminished to 70% after 36 h along with the recovery of excess starting material.

**Scheme 2:**
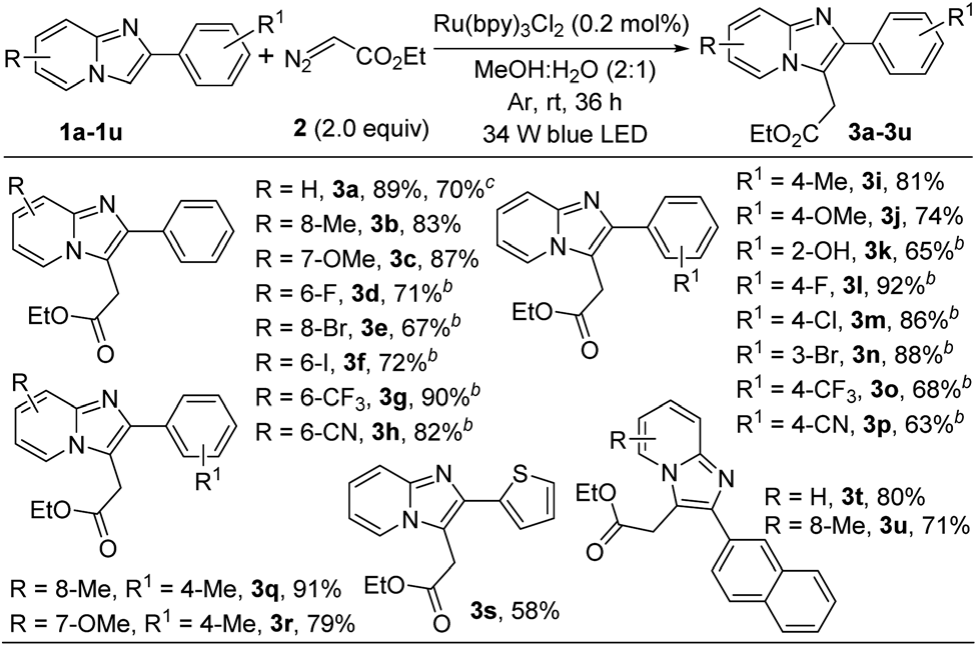
Substrate scope of imidazopyridines.^*a*^ ^*a*^Reaction conditions: 0.25 mmol of 1, 0.5 mmol of 2 in presence of 0.2 mol% of Ru(bpy)_3_Cl_2_ in 2.0 mL of MeOH : H_2_O (2 : 1) at room temperature under 34 W blue LED and argon atmosphere for 36 h. ^*b*^10 mol% *N*,*N*-dimethyl-*m*-toluidine was added as an additive. ^*c*^6.0 mmol scale.

Next, to show the generality of this present methodology, we investigated the reaction taking other imidazoheterocycles like imidazo[2,1-*b*]thiazole and benzo[*d*]imidazo[2,1-*b*]thiazoles (Scheme 3). 6-Phenylimidazo-[2,1-*b*]thiazole reacted smoothly to afford 5a in 66% yield. Benzo[*d*]imidazo[2,1-*b*]thiazole containing –Me, –OMe, and –Cl substituted derivatives were well tolerable under the present reaction conditions (5b–5e). The structure of ethyl 2-(7-methoxy-2-phenylbenzo[*d*]imidazo[2,1-*b*]thiazol-3-yl)acetate (5c) was confirmed by X-ray crystallography.^14^ In addition, thiophene substituted benzo[*d*]imidazo[2,1-*b*]thiazole furnished the corresponding alkylated product in good yield (5f).

**Scheme 3:**
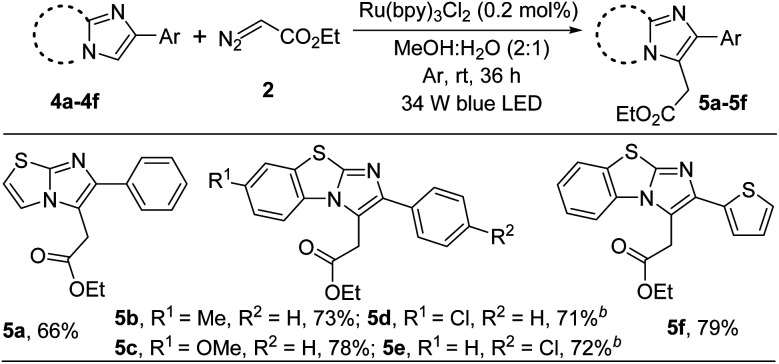
Substrate scope.^*a*^ ^*a*^Reaction conditions: 0.25 mmol of 4, 0.5 mmol of 2 in presence of 0.2 mol% of Ru(bpy)_3_Cl_2_ in 2.0 mL of MeOH : H_2_O (2 : 1) at room temperature under 34 W blue LED and argon atmosphere for 36 h. ^*b*^10 mol% *N*,*N*-dimethyl-*m*-toluidine was added as an additive.

After that, we have directly modified the synthesized ethoxycarbonylmethylated product (3q) to amide derivative (6q) in 56% yield using benzylamine in the presence of La(OTf)_3_ as a catalyst under ambient air at rt to 70 °C as shown in Fig. 1.

**Fig. 1:**
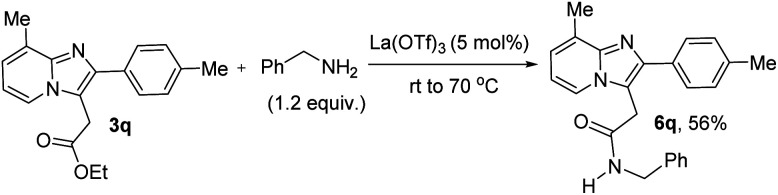
Modification of ester group *via* direct amidation process.

A few control experiments were performed to understand the mechanistic insights of the present protocol (Scheme 4). The alkylation reaction did not proceed in the presence of 3.0 equiv. radical scavengers like 2,2,6,6-tetramethylpiperidine-1-oxyl (TEMPO), 2,6-di-*tert*-butyl-4-methyl phenol (BHT), 1,1-diphenylethylene (DPE), and *p*-benzoquinone (BQ) which suggest the reaction probably proceeds through radical pathway (Scheme 4, eqn (A)). Moreover, 3-phenylimidazo[1,2-*a*]pyridine (1v) did not produce the C-2 alkylated product that signifies the regioselectivity of the present protocol (Scheme 4, eqn (B)). Taking 2-(2-(vinyloxy)phenyl)imidazo[1,2-*a*]pyridine we also performed this reaction with ethyl diazoacetate under this standard reaction conditions. Desired C-3 alkylated product (8a) was formed in 35% yield (Scheme 4, eqn (C)). But cyclopropanation product was not formed in the alkene part of the allyl group *via* carbene pathway. From this experimental result strongly imply that the present strategy only proceeds through radical mechanistic pathway.

**Scheme 4:**
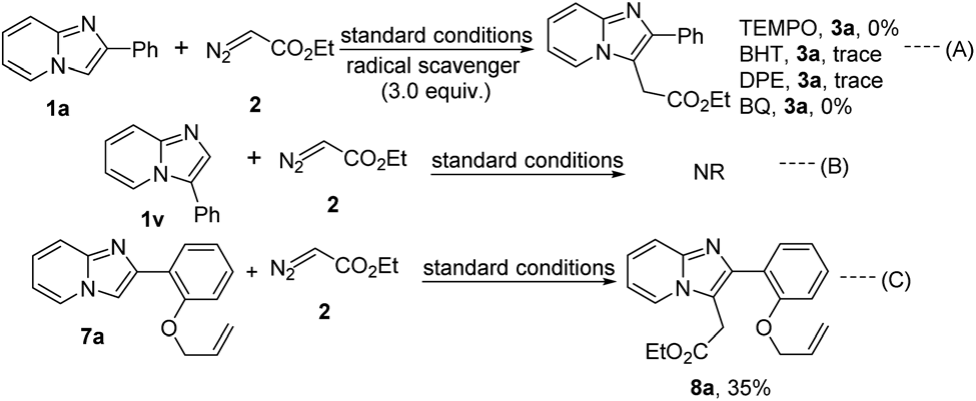
Control experiments.

Based on the above mechanistic studies and previous literature reports,^7,8,13^ a possible radical mechanism was proposed for the formation of ethyl 2-(2-phenylimidazo[1,2-*a*]pyridin-3-yl)acetate (3a) as presented in Scheme 5. Initially, photo-catalyst Ru(bpy)_3_^2+^ is transformed to its excited-state *Ru(bpy)_3_^2+^ under blue LED irradiation. In the presence of MeOH/H_2_O, diazo compounds are in equilibrium with their protonated form (A). Then cationic diazoester involves single-electron transfer (SET) process with excited photocatalyst *Ru(bpy)_3_^2+^ leading to the formation of alkyl radical intermediate (B) and Ru(bpy)_3_^3+^.^6*b*,*c*^ Next, alkyl radical intermediate (B) effectively reacts with imidazopyridine (1a) to produce imidazole radical intermediate (C) which is successively transformed to imidazolium radical cation intermediate (D) along with the generation of ground state photocatalyst. Finally, C-3 alkylated imidazopyridine (3a) is obtained through deprotonation of intermediate D. It is worthy to mention that Meggers *et al.*^7*h*^ did not observe quenching of *Ru(bpy)_3_^2+^ whereas Gryko *et al.*^6*c*^ did observe the quenching of *Ru(bpy)_3_^2+^ by ethyl diazoacetate that was accelerated in the presence of protic sources.

**Scheme 5:**
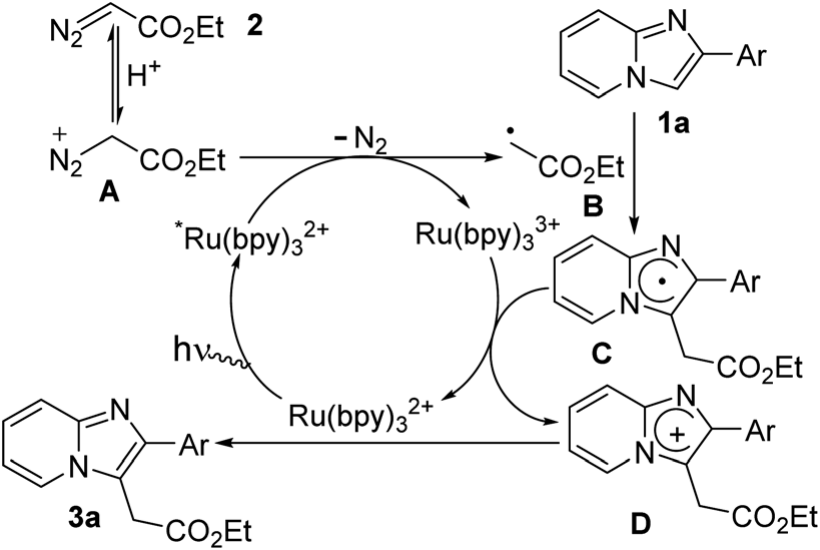
Proposed mechanistic pathway.

In case of halogen or electron-withdrawing substituted imidazoheterocycles a possible mechanism for the radical alkylation reaction is illustrated in Scheme 6.^6*b*,*c*^ Reductive quenching of *Ru(bpy)_3_^2+^ by SET oxidation of aniline derivatives affords radical cation E and Ru(bpy)_3_^+^. Then reduced photocatalyst Ru(bpy)_3_^+^ comes back to ground state with the generation of radical intermediate B*via* the elimination of N_2_. Subsequently, radical intermediate B reacts with imidazopyridine 1 to produce radical intermediate C′. Finally, the desired product 3′ is produced through the hydrogen atom transfer (HAT) process between radical cation E and radical intermediate C′.

**Scheme 6:**
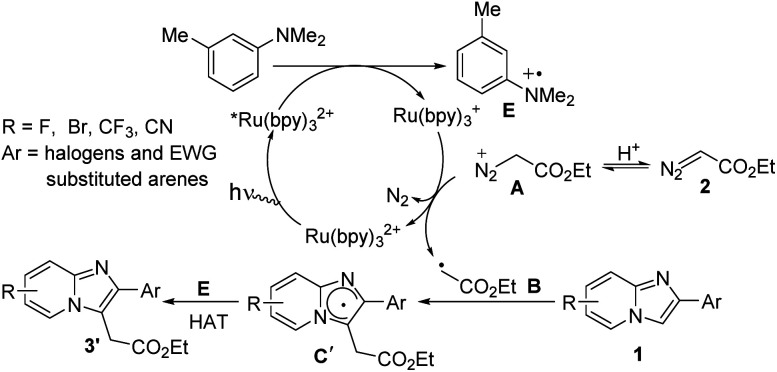
Mechanistic pathway for electon-deficient substrates.

## Conclusions

In summary, we have developed an efficient photoredox-catalyzed ethoxycarbonylmethylation of imidazoheterocycles with ethyl diazoester as an alkylating reagent under the irradiation of blue LED at room temperature. A wide variety of functionalized imidazoheterocycles were well tolerated under the mild reaction conditions. A gram-scale synthesis was also performed to demonstrate the potential synthetic application of this new method. In contrast to the carbene precursors from α-diazoester a first C3-ethoxycarbonylmethylation of imidazopyridines *via* radical intermediate has been established. We believe the present protocol to be of significant applicability for the diversification of heterocyclic moieties in a multitude of medicinal applications.

## Conflicts of interest

There are no conflicts to declare.

## Supplementary Material

RA-010-D0RA05795A-s001

RA-010-D0RA05795A-s002
