# Supplementary material for: Visible light-induced photocatalytic C–H ethoxycarbonylmethylation of imidazoheterocycles with ethyl diazoacetate
Source: RSC Adv. 2020 Jul 28;10(47):27984–8. doi: 10.1039/d0ra05795a (PMC9055643; doi:10.1039/d0ra05795a)
Supplement: RA-010-D0RA05795A-s001 [file RA-010-D0RA05795A-s001.pdf]

Electronic Supplementary Material (ESI) for RSC Advances.
This journal is © The Royal Society of Chemistry 2020

Supporting Information

Visible Light-Induced Photocatalytic C-H Ethoxycarbonylmethylation of Imidazoheterocycles with Ethyl Diazoacetate

Suvam Bhattacharjee, Sudip Laru, Sadhanendu Samanta, Mukta Singsardar, and Alakananda Hajra*

Department of Chemistry, Visva-Bharati (A Central University), Santiniketan 731235, India

Email: alakananda.hajra@visva-bharati.ac.in

Contents

Sl. No.	Topics	Page No.
1.	General Information	S2
Table S1.	Optimization of the Reaction Conditions	S2
2.	Reaction setup	S3-S4
3.	Structure determination (X-ray crystallographic data for **5c**):	S5
4.	Characterization data for the synthesized compounds (**1c**):	S6
5.	General experimental procedure for the synthesized compounds (**3a**-**5f**):	S6
6.	Characterization data for the synthesized compounds (**3a**-**8a**):	S7-S19
7.	NMR spectra [^{1}H and $^{13}C\{^{1}H\}$)] of synthesized products	S20-S80

1. General Information: All reagents were bought from commercial sources and used as recieved without further purification. All reactions involving moisture sensitive reactants were executed using oven dried glassware. All commercially available solvents were used after distillation.^{1}H NMR spectra were determined on 400 MHz spectrometer as solutions in $CDCl_3$ and $^{13}C\{^{1}H\}$ spectra were recorded at 100 MHz spectrometer in $CDCl_3$ solution. Chemical shifts (δ) are expressed in parts per million (ppm) and coupling constants (J) are given in Hz. Chemical shifts are referenced to $CDCl_3$ (δ = 7.26 for ^{1}H and δ = 77.16 for $^{13}C\{^{1}H\}$ NMR) as internal standard. NMR spectra use the following abbreviations to describe the multiplicity: s (singlet), d (doublet), t (triplet), q (quartet) and m (multiplet). The progress of reaction was checked by TLC plates (silica gel coated glass slide) and the spots were visualized under UV light. Melting points (M.p.) were determined after recrystallization of solid compounds from a solution of dichloromethane/petroleum ether (1:3).

Table S1. Optimization of the Reaction Conditions[a]

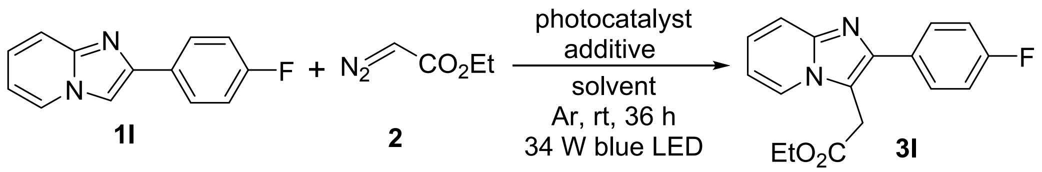

Entry	Photocatalyst (0.2 mol %)	Additive (10 mol %)	Solvent	Yield (%)
1	$Ru(bpy)_3Cl_2$	-	$MeOH:H_2O$ (2:1)	trace
2	$Ru(bpy)_3Cl_2$	aniline	$MeOH:H_2O$ (2:1)	42
3	$Ru(bpy)_3Cl_2$	DBU	$MeOH:H_2O$ (2:1)	11
4	$Ru(bpy)_3Cl_2$	TEA	$MeOH:H_2O$ (2:1)	38
5	$Ru(bpy)_3Cl_2$	*N*,*N*-dimethyl aniline	$MeOH:H_2O$ (2:1)	78
**6**	**$Ru(bpy)_3Cl_2$**	***N*,*N*-dimethyl *m*-toluidine**	**$MeOH:H_2O$ (2:1)**	**92**
7	$Ru(bpy)_3Cl_2$	DABCO	$MeOH:H_2O$ (2:1)	24

[a]Reaction conditions: 0.25 mmol of **1l**, 0.5 mmol of **2** in presence of 0.2 mol% of $Ru(bpy)_3Cl_2$ and 10 mol% of additive in 2.0 mL of $MeOH:H_2O$ (2:1) at room temperature under 34 W blue LED and argon atmosphere for 36 h.

2. Reaction setup

The reaction was irradiated with Kessil 34 W blue LED (Model No: H150-Blue). The LED light was positioned 7 cm away from the reaction vessel. The description of this light is given below:

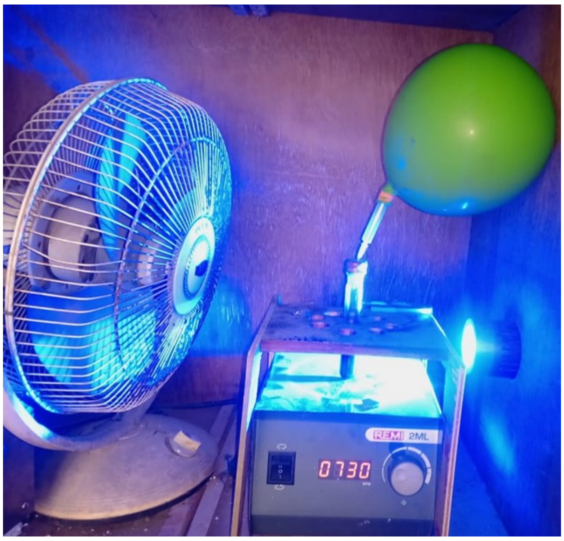

Figure S1. Milligram scale reaction set up

Description for Kessil (34 W) Blue LED

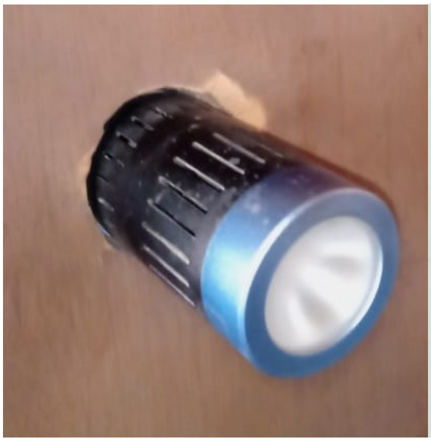

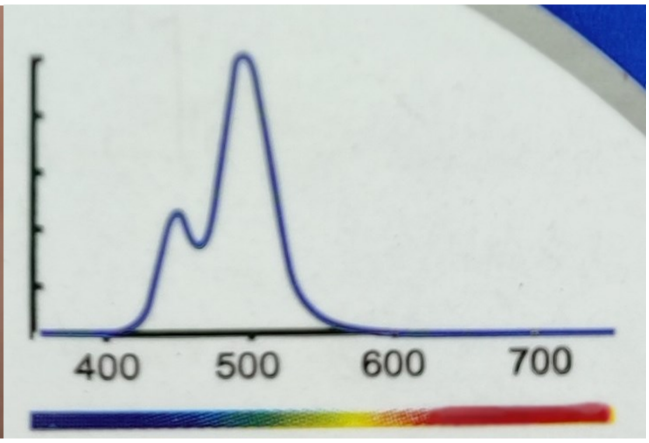

Figure S2. Kessil H150 Blue LED **Figure S3.** Emission spectrum of 34 W blue LED

	Specification
Power	34 W
Voltage	1.5 A
Wavelength	~ 450, 495 nm (max)

Figure S4. Description for 34 W blue LED light

For optimizing the reaction conditions, photo-induced reaction was also checked with commercially available 30 W white LED light, which was positioned 7 cm away from the reaction vessel but no desired product was produced. The description of this light is given below. The reaction is not significantly affected by temperature. The fan was used to maintain temperature of the reactor \(20 ~ 30 ^{o}C). There is no spectral description.

Description for 30 W white LED light

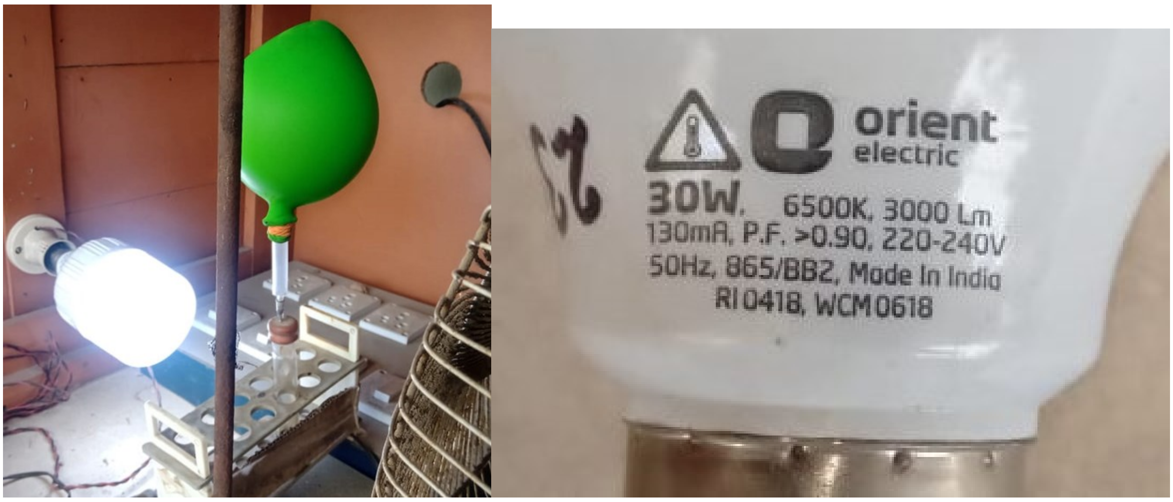

Figure S5. Set up of 30 W white LED

	Specification
Power	30 W
Voltage	130 mA

Figure S6. Description for 30 W white LED

3. Structure determination (X-ray crystallographic data for 5c):

The white crystal of **5c** was obtained by crystallization from a solution in dichloromethane/petroleum ether after purification by column chromatography. Chemical formula $C_{20}H_{18}N_2O_3S$.

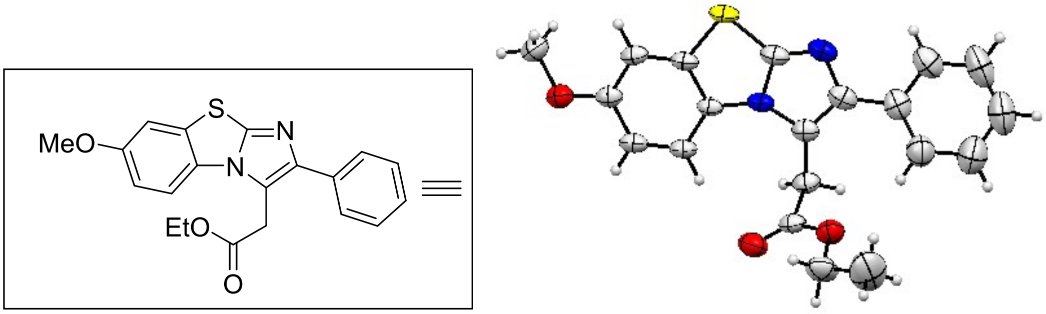

ORTEP (with 50% probability) diagram for the structure ethyl 2-(7-methoxy-2-phenylbenzo[d]imidazo[2,1-b]thiazol-3-yl)acetate (**5c**).

**Wavelength**	0.71073 Å	
**Formula**	$C_{20}H_{18}N_2O_3S$	
**Crystal system**	orthorhombic	
**Space group**	P c a 21	
**Unit cell dimensions**	a = 7.0148(14)Å	α = 90 °
	b = 17.582(4)Å	β = 90 °
	c = 14.402(3)Å	γ = 90 °
**Volume**	1776.2(6)$Å^3$	
**Z**	4	
**R-factor (%)**	4.16	

The crystallographic data have been deposited with the Cambridge Crystallographic Data Centre as supplementary publication with a CCDC reference number CCDC **1976157**.

4. Characterization data for the synthesized compounds (1c):

Starting Materials: All the imidazoheterocycles were prepared by our reported method.[13]

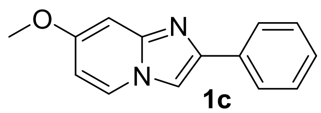

1c

7-Methoxy-2-phenylimidazo[1,2-*a*]pyridine (1c):[13] Yellow solid (3.0 mmol, 88%, 592 mg); ^{1}H NMR ($CDCl_3$, 400 MHz): δ 7.90-7.86 (m, 3H), 7.65 (s, 1H), 7.40 (t, J = 7.6 Hz, 2H), 7.29 (t, J = 7.6 Hz, 1H), 6.89 (s, 1H), 6.48-6.45 (m, 1H), 3.83 (s, 3H); ^{13}C{^{1}H} NMR ($CDCl_3$, 100 MHz): δ 158.0, 147.2, 145.4, 133.9, 128.7, 127.7, 126.0, 125.8, 107.6, 106.9, 94.7, 55.5.

5. General experimental procedure for the synthesized compounds (3a-5f):

A mixture of 2-phenylimidazo[1,2-*a*]pyridine (**1a**, 0.25 mmol, 48.5 mg), ethyl diazoacetate (**2**, 0.5 mmol, 40 µL, containing ~15% of toluene as a stabilizer) and 0.2 mol % $Ru(bpy)_3Cl_2$ (0.2 mol %, 0.3 mg) were taken in an oven-dried reaction vessel equipped with a magnetic stir bar under argon atmosphere. Then (2:1) solvent mixture MeOH:H_2O (2.0 mL) was added to the reaction. After that the reaction mixture was stirred under the irradiation of 34 W blue LED (Kessil 34 W) at room temperature for 36 h. The progress of the reaction was monitored by TLC. After completion, the reaction mixture was evaporated and quenched with 10 mL water/ethyl acetate (1:3). Then the reaction mixture was extracted with ethyl acetate and the organic phase was dried over anhydrous Na_2SO_4. The crude residue was obtained after evaporating the solvent in vacuum and was purified by column chromatography on silica gel (100-200 mesh) using a mixture of petroleum ether and ethyl acetate as an eluting solvent to afford the pure product.

6. Characterization data for the synthesized compounds (3a-8a):

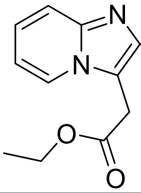

3a

Ethyl 2-(2-phenylimidazo[1,2-*a*]pyridin-3-yl)acetate (**3a**):[11c] Yellow solid (62 mg, 89%); M.p. 127-128 ^{o}C; ^{1}H NMR (CDCl$_3$, 400 MHz): δ 8.12 (d, J = 6.8 Hz, 1H), 7.83 (d, J = 7.2 Hz, 2H), 7.67 (d, J = 8.8 Hz, 1H), 7.48 (t, J = 7.6 Hz, 2H), 7.38 (t, J = 7.6 Hz, 1H), 7.25-7.21 (m, 1H), 6.87 (t, J = 6.8 Hz, 1H), 4.22 (q, J = 7.2 Hz, 2H), 4.04 (s, 2H), 1.27 (t, J = 7.2 Hz, 3H); ^{13}C{^{1}H} NMR (CDCl$_3$, 100 MHz): δ 169.5, 145.1, 144.7, 134.1, 128.7, 128.0, 124.6, 123.8, 118.0, 117.7, 113.1, 112.5, 61.7, 30.9, 14.2.

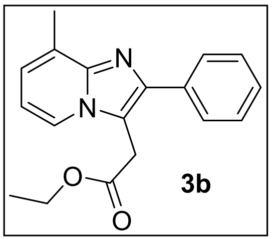

3b

Ethyl 2-(8-methyl-2-phenylimidazo[1,2-*a*]pyridin-3-yl)acetate (**3b**):[2b] Yellow solid (61 mg, 83%); M.p. 85-86 ^{o}C; ^{1}H NMR (CDCl$_3$, 400 MHz): δ 7.98 (d, J = 6.8 Hz, 1H), 7.84-7.82 (m, 2H), 7.47 (t, J = 7.6 Hz, 2H), 7.39-7.35 (m, 1H), 7.03-7.01 (m, 1H), 6.78 (t, J = 6.8 Hz, 1H), 4.21 (q, J = 7.2 Hz, 2H), 4.01 (s, 2H), 2.67 (s, 3H), 1.27 (t, J = 7.2 Hz, 3H); ^{13}C{^{1}H} NMR (CDCl$_3$, 100 MHz): δ 169.6, 145.5, 144.3, 134.4, 128.9, 128.7, 127.9, 127.7, 123.4, 121.6, 113.4, 112.5, 61.7, 31.0, 17.2, 14.3.

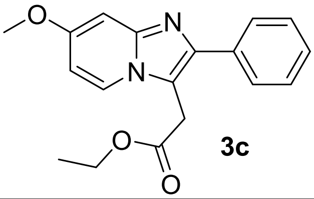

3c

Ethyl 2-(7-methoxy-2-phenylimidazo[1,2-*a*]pyridin-3-yl)acetate (**3c**): Yellow gummy mass (67 mg, 87%); ^{1}H NMR ($CDCl_3$, 400 MHz): δ 7.96 (d, J = 7.6 Hz, 1H), 7.82-7.80 (m, 2H), 7.46 (t, J = 7.6 Hz, 2H), 7.36 (t, J = 7.6 Hz, 1H), 6.93 (d, J = 2.4 Hz, 1H), 6.59-6.56 (m, 1H), 4.21 (q, J = 7.2 Hz, 2H), 3.98 (s, 2H), 3.86 (s, 3H), 1.27 (t, J = 7.2 Hz, 3H); ^{13}C{^{1}H} NMR ($CDCl_3$, 100 MHz): δ 169.6, 158.2, 146.4, 143.7, 133.9, 128.7, 128.4, 127.9, 124.4, 111.8, 107.6, 94.7, 61.7, 55.7, 30.8, 14.2; Anal. Calcd for $C_{18}H_{18}N_2O_3$: C, 69.66; H, 5.85; N, 9.03%; Found C, 69.49; H, 5.80; N, 9.10%.

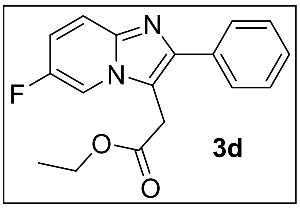

Ethyl 2-(6-fluoro-2-phenylimidazo[1,2-*a*]pyridin-3-yl)acetate (**3d**):[2b] Yellow gummy mass (53 mg, 71%); ^{1}H NMR ($CDCl_3$, 400 MHz): δ 8.10-8.08 (m,1H), 7.81-7.79 (m, 2H), 7.68-7.64 (m, 1H), 7.48 (t, J = 7.6 Hz, 2H), 7.43-7.34 (m, 1H), 7.18-7.13 (m, 1H), 4.24 (q, J = 7.2 Hz, 2H), 4.01 (s, 2H), 1.29 (t, J = 7.2 Hz, 3H); ^{13}C{^{1}H} NMR ($CDCl_3$, 100 MHz): δ 169.1, 153.4 ($J_{C\text{-}F}$ = 236.0 Hz), 146.0, 142.8, 133.8, 128.7 ($J_{C\text{-}F}$ = 18.0 Hz), 128.2, 126.1, 118.1 ($J_{C\text{-}F}$ = 10.0 Hz), 116.5 ($J_{C\text{-}F}$ = 26.0 Hz), 114.5, 110.6 ($J_{C\text{-}F}$ = 41.0 Hz), 61.8, 31.0, 14.2.

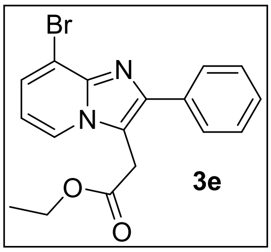

Ethyl 2-(8-bromo-2-phenylimidazo[1,2-*a*]pyridin-3-yl)acetate (**3e**): Yellow gummy mass (60 mg, 67%); ^{1}H NMR ($CDCl_3$, 400 MHz): δ 8.11-8.09 (m, 1H), 7.84-7.82 (m, 2H), 7.49-7.45 (m, 3H), 7.41-7.36 (m, 1H), 6.75-6.72 (m, 1H), 4.21 (q, J = 7.2 Hz, 2H), 4.01 (s, 2H), 1.26 (t, J = 7.2 Hz, 3H); ^{13}C{^{1}H} NMR ($CDCl_3$, 100 MHz): δ 169.2, 145.5, 143.0, 133.6,

129.0, 128.7, 128.2, 127.0, 123.2, 114.9, 112.5, 111.8, 61.8, 31.1, 14.2; Anal. Calcd for $C_{17}H_{15}BrN_2O_2$: C, 56.84; H, 4.21; N, 7.80%; Found C, 56.70; H, 4.25; N, 7.88%.

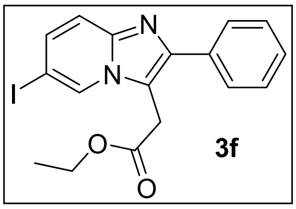

Ethyl 2-(6-iodo-2-phenylimidazo[1,2-*a*]pyridin-3-yl)acetate (3f): Yellow solid (73 mg, 72%); M.p. 98-99 °C; ^{1}H NMR ($CDCl_3$, 400 MHz): δ 8.42 (s, 1H), 7.81 (d, J = 7.2 Hz, 2H), 7.56 (d, J = 9.6 Hz, 1H), 7.53-7.39 (m, 4H), 4.25 (q, J = 7.2 Hz, 2H), 4.03 (s, 2H), 1.31 (t, J = 7.2 Hz, 3H); ^{13}C{^{1}H} NMR ($CDCl_3$, 100 MHz): δ 169.1, 145.2, 143.7, 133.6, 132.5, 129.0, 128.8, 128.7, 128.3, 126.3, 118.7, 113.1, 61.9, 30.9, 14.3; Anal. Calcd for $C_{17}H_{15}IN_2O_2$: C, 50.26; H, 3.72; N, 6.90%; Found C, 50.43; H, 3.78; N, 6.79%.

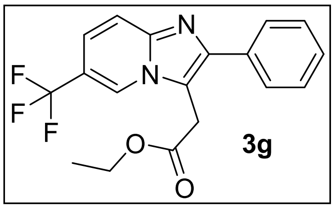

Ethyl 2-(2-phenyl-6-(trifluoromethyl)imidazo[1,2-*a*]pyridin-3-yl)acetate (**3g**): Yellow solid (78 mg, 90%); M.p. 82-83 °C; ^{1}H NMR ($CDCl_3$, 400 MHz): δ 8.54 (s, 1H), 7.82 (d, J = 7.2 Hz, 2H), 7.74 (d, J = 9.6 Hz, 1H), 7.49 (t, J = 7.6 Hz, 2H), 7.42-7.36 (m, 2H), 4.24 (q, J = 7.2 Hz, 2H), 4.07 (s, 2H), 1.29 (t, J = 7.2 Hz, 3H); ^{13}C{^{1}H} NMR ($CDCl_3$, 100 MHz): δ 168.9, 145.7 ($J_{C\text{-}F}$ = 175.0 Hz), 133.3, 128.8 ($J_{C\text{-}F}$ = 12.0 Hz), 128.5, 125.1, 119.8 (q, $J_{C\text{-}F}$ = 263.0 Hz), 123.2 (q, $J_{C\text{-}F}$ = 6.0 Hz), 122.4, 120.4, 118.3, 117.3 (q, $J_{C\text{-}F}$ = 34.0 Hz), 114.5, 62.0, 30.8, 14.1; Anal. Calcd for $C_{18}H_{15}F_3N_2O_2$: C, 62.07; H, 4.34; N, 8.04%; Found C, 62.22; H, 4.39; N, 7.91%.

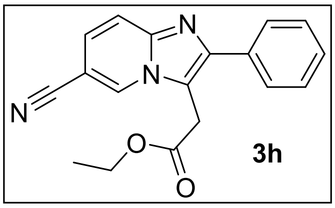

Ethyl 2-(6-cyano-2-phenylimidazo[1,2-*a*]pyridin-3-yl)acetate (**3h**): Yellow gummy mass (62 mg, 82%); ^{1}H NMR (CDCl$_3$, 400 MHz): δ 8.62 (s, 1H), 7.82-7.80 (m, 2H), 7.73 (d, J = 9.6 Hz, 1H), 7.51 (t, J = 7.6 Hz, 2H), 7.43 (t, J = 7.6 Hz, 1H), 7.35-7.32 (m, 1H), 4.26 (q, J = 7.2 Hz, 2H), 4.08 (s, 2H), 1.32 (t, J = 7.2 Hz, 3H); ^{13}C{^{1}H} NMR (CDCl$_3$, 100 MHz): δ 168.8, 144.3, 132.9, 132.8, 130.3, 129.0, 128.89, 128.81, 124.4, 118.6, 116.9, 114.5, 98.7, 62.2, 30.7, 14.3; Anal. Calcd for $C_{18}H_{15}N_3O_2$: C, 70.81; H, 4.95; N, 13.76%; Found C, 71.01; H, 5.01; N, 13.66%.

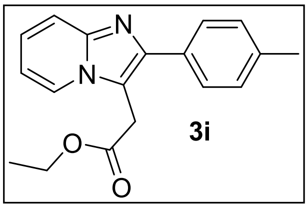

Ethyl 2-(2-(*p*-tolyl)imidazo[1,2-*a*]pyridin-3-yl)acetate (**3i**):[2b] Yellow gummy mass (59 mg, 81%); ^{1}H NMR (CDCl$_3$, 400 MHz): δ 8.12 (d, J = 6.8 Hz, 1H), 7.74-7.72 (m, 2H), 7.68-7.65 (m, 1H), 7.29 (d, J = 8.0 Hz, 2H), 7.24-7.20 (m, 1H), 6.88-6.84 (m, 1H), 4.22 (q, J = 7.2 Hz, 2H), 4.03 (s, 2H), 2.41 (s, 3H), 1.27 (t, J = 7.2 Hz, 3H); ^{13}C{^{1}H} NMR (CDCl$_3$, 100 MHz): δ 169.6, 145.0, 144.7, 137.9, 131.2, 129.5, 128.6, 124.5, 123.8, 117.6, 112.8, 112.4, 61.7, 31.0, 21.4, 14.3.

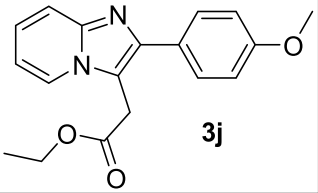

Ethyl 2-(2-(4-methoxyphenyl)imidazo[1,2-*a*]pyridin-3-yl)acetate (**3j**):[2b] Yellow gummy mass (57.5 mg, 74%); ^{1}H NMR (CDCl$_3$, 400 MHz): δ 8.09 (d, J = 6.8 Hz, 1H), 7.77-7.75 (m,

2H), 7.64-7.61 (m, 1H), 7.21-7.17 (m, 1H), 7.00 (d, J = 8.8 Hz, 2H), 6.85-6.81 (m, 1H), 4.20 (q, J = 7.2 Hz, 2H), 3.99 (s, 2H), 3.83 (s, 3H), 1.25 (t, J = 7.2 Hz, 3H); $^{13}C\{^1H\}$ NMR ($CDCl_3$, 100 MHz): δ 169.5, 159.6, 144.9, 144.4, 130.3, 129.8, 126.5, 124.5, 123.7, 117.3, 114.1, 112.3, 61.6, 55.3, 30.9, 14.2.

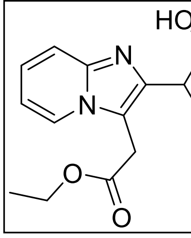

3k

Ethyl 2-(2-(2-hydroxyphenyl)imidazo[1,2-*a*]pyridin-3-yl)acetate *(3k)*: Yellow gummy mass (48 mg, 65%); ^1H NMR ($CDCl_3$, 400 MHz): δ 8.20 (d, J = 7.2 Hz, 1H), 7.73-7.71 (m, 1H), 7.62 (d, J = 9.2 Hz, 1H), 7.32-7.28 (m, 2H), 7.08-7.06 (m, 1H), 6.97-6.95 (m, 2H), 4.24 (q, J = 7.2 Hz, 2H), 4.13 (s, 2H), 1.28 (t, J = 7.2 Hz, 3H); $^{13}C\{^1H\}$ NMR ($CDCl_3$, 100 MHz): δ 169.2, 157.5, 143.3, 142.4, 129.9, 127.5, 125.4, 123.6, 119.3, 117.6, 117.2, 116.9, 113.2, 112.6, 61.9, 31.2, 14.2; Anal. Calcd for $C_{17}H_{16}N_2O_3$: C, 68.91; H, 5.44; N, 9.45%; Found: C, 68.67; H, 5.38; N, 9.57%.

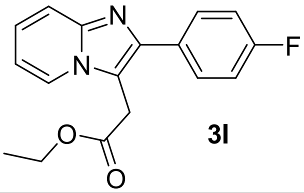

3l

Ethyl 2-(2-(4-fluorophenyl)imidazo[1,2-*a*]pyridin-3-yl)acetate (**3l**): Yellow solid (68 mg, 92%); M.p. 92-93 °C; ^1H NMR ($CDCl_3$, 400 MHz): δ 8.11 (d, J = 6.8 Hz, 1H), 7.83-7.78 (m, 2H), 7.63 (d, J = 9.2 Hz, 1H), 7.23-7.12 (m, 3H), 6.85 (t, J = 6.8 Hz, 1H), 4.20 (q, J = 7.2 Hz, 2H), 3.99 (s, 2H), 1.26 (t, J = 7.2 Hz, 3H); $^{13}C\{^1H\}$ NMR ($CDCl_3$, 100 MHz): δ 169.4, 162.8 (J_{C-F} = 245.0 Hz), 145.0, 143.8, 130.3 (J_{C-F} = 8.0 Hz), 130.3, 130.2, 124.7, 123.8, 117.6, 115.6 (J_{C-F} = 21.0 Hz), 112.7 (J_{C-F} = 30.0 Hz), 61.7, 30.8, 14.2; Anal. Calcd for $C_{17}H_{15}FN_2O_2$: C, 68.45; H, 5.07; N, 9.39%; Found C, 68.24; H, 5.10; N, 9.29%.

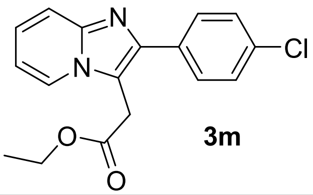

3m

Ethyl 2-(2-(4-chlorophenyl)imidazo[1,2-*a*]pyridin-3-yl)acetate (**3m**):[2b] Yellow gummy mass (67 mg, 86%); ^{1}H NMR ($CDCl_3$, 400 MHz): δ 8.12 (d, J = 7.2 Hz, 1H), 7.78 (d, J = 8.4 Hz, 2H), 7.63 (d, J = 9.2 Hz, 1H), 7.43 (d, J = 8.4 Hz, 2H), 7.24-7.20 (m, 1H), 6.86 (t, J = 7.2 Hz, 1H), 4.20 (q, J = 7.2 Hz, 2H), 3.99 (s, 2H), 1.26 (t, J = 7.2 Hz, 3H); $^{13}C\{^1H\}$ NMR ($CDCl_3$, 100 MHz): δ 169.3, 145.1, 143.5, 134.0, 132.6, 129.8, 128.9, 124.8, 123.8, 117.6, 113.1, 112.6, 61.8, 30.9, 14.2; HRMS (ESI-TOF) m/z: [M + H^+] Calcd for $C_{17}H_{16}ClN_2O_2$: 315.0895; Found 315.0894.

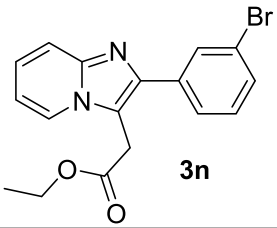

3n

Ethyl 2-(2-(3-bromophenyl)imidazo[1,2-*a*]pyridin-3-yl)acetate (**3n**):[11c] Yellow gummy mass (79 mg, 88%); ^{1}H NMR ($CDCl_3$, 400 MHz): δ 8.13 (d, J = 7.2 Hz, 1H), 8.04 (s, 1H), 7.76 (d, J = 7.6 Hz, 1H), 7.63 (d, J = 8.2 Hz, 1H), 7.49 (d, J = 8.0 Hz, 1H), 7.32 (t, J = 8.4 Hz, 1H), 7.24-7.20 (m, 1H), 6.88-6.84 (m, 1H), 4.21 (q, J = 7.2 Hz, 2H), 4.00 (s, 2H), 1.28 (t, J = 7.2 Hz, 3H); $^{13}C\{^1H\}$ NMR ($CDCl_3$, 100 MHz): δ 169.2, 145.1, 143.1, 136.2, 131.5, 130.9, 130.2, 127.1, 124.9, 123.8, 122.8, 117.7, 113.4, 112.7, 61.8, 30.8, 14.2.

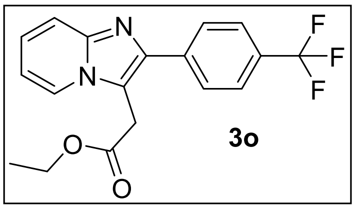

3o

Ethyl 2-(2-(4-(trifluoromethyl)phenyl)imidazo[1,2-*a*]pyridin-3-yl)acetate (**3o**): Yellow solid (59 mg, 68%); M.p. 88-89 ^oC; ^{1}H NMR ($CDCl_3$, 400 MHz): δ 8.16 (d, J = 6.8 Hz, 1H),

7.99 (d, J = 8.0 Hz, 2H), 7.73 (d, J = 8.0 Hz, 2H), 7.67 (d, J = 9.2 Hz, 1H), 7.29-7.25 (m, 1H), 6.92-6.89 (m, 1H), 4.23 (q, J = 7.2 Hz, 2H), 4.04 (s, 2H), 1.29 (t, J = 7.2 Hz, 3H); $^{13}C\{^1H\}$ NMR ($CDCl_3$, 100 MHz): δ 169.2, 145.3, 143.2, 137.7, 129.4, 128.8, 125.5 (q, $J_{C\text{-}F}$ = 4.0 Hz), 125.1, 124.7, 123.9, 119.6 (q, $J_{C\text{-}F}$ = 258.0 Hz), 117.9, 112.9, 61.9, 30.9, 14.2; Anal. Calcd for $C_{18}H_{15}F_3N_2O_2$: C, 62.07; H, 4.34; N, 8.04%; Found C, 62.27; H, 4.30; N, 8.13%.

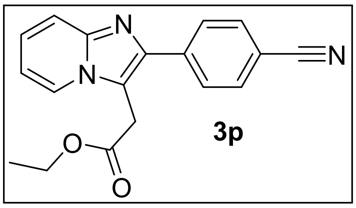

Ethyl 2-(2-(4-cyanophenyl)imidazo[1,2-*a*]pyridin-3-yl)acetate (**3p**): Yellow gummy mass (48 mg, 63%); ^1H NMR ($CDCl_3$, 400 MHz): δ 8.16 (d, J = 6.8 Hz, 1H), 7.99 (d, J = 8.4 Hz, 2H), 7.78-7.74 (m, 2H), 7.66 (d, J = 8.8 Hz, 1H), 7.29-7.25 (m, 1H), 6.93-6.90 (m, 1H), 4.23 (q, J = 7.2 Hz, 2H), 4.03 (s, 2H), 1.28 (t, J = 7.2 Hz, 3H); $^{13}C\{^1H\}$ NMR ($CDCl_3$, 100 MHz): δ 169.0, 145.4, 142.6, 138.8, 132.5, 129.0, 125.4, 123.9, 119.0, 117.9, 114.2, 113.0, 111.4, 62.0, 30.9, 14.2; Anal. Calcd for $C_{18}H_{15}N_3O_2$: C, 70.81; H, 4.95; N, 13.76%; Found: C, 70.65; H, 4.88; N, 13.84%.

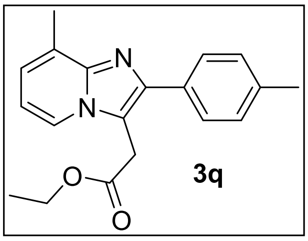

Ethyl 2-(8-methyl-2-(*p*-tolyl)imidazo[1,2-*a*]pyridin-3-yl)acetate (**3q**): Brown gummy mass (70 mg, 91%); ^1H NMR ($CDCl_3$, 400 MHz): δ 7.95 (d, J = 7.2 Hz, 1H), 7.72 (d, J = 8.0 Hz, 2H), 7.28 (d, J = 8.0 Hz, 2H), 7.00-6.98 (m, 1H), 6.75 (t, J = 6.8 Hz, 1H), 4.19 (q, J = 7.2 Hz, 2H), 3.99 (s, 2H), 2.65 (s, 3H), 2.40 (s, 3H), 1.25 (t, J = 7.2 Hz, 3H); $^{13}C\{^1H\}$ NMR ($CDCl_3$, 100 MHz): δ 169.6, 145.4, 144.2, 137.5, 131.4, 129.3, 128.7, 127.4, 123.2, 121.5, 113.1,

112.3, 61.5, 30.9, 21.3, 17.2, 14.2; HRMS (ESI-TOF) m/z: [M + H$^+$] Calcd for $C_{19}H_{21}N_2O_2$: 309.1598; Found 309.1603.

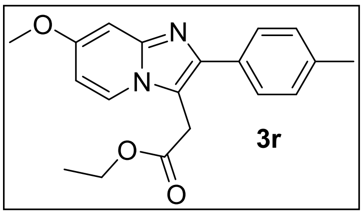

Ethyl 2-(7-methoxy-2-(*p*-tolyl)imidazo[1,2-*a*]pyridin-3-yl)acetate (**3r**): Yellow gummy mass (64 mg, 79%); ^{1}H NMR (CDCl$_3$, 400 MHz): δ 7.96 (d, J = 7.6 Hz, 1H), 7.70 (d, J = 8.0 Hz, 2H), 7.27 (d, J = 7.6 Hz, 2H), 6.98 (d, J = 2.4 Hz, 1H), 6.60-6.58 (m, 1H), 4.21 (q, J = 7.2 Hz, 2H), 3.97 (s, 2H), 3.87 (s, 3H), 2.40 (s, 3H), 1.27 (t, J = 7.2 Hz, 3H); ^{13}C{^{1}H} NMR (CDCl$_3$, 100 MHz): δ 169.6, 158.3, 146.2, 143.5, 137.8, 130.9, 129.4, 128.8, 124.4, 111.6, 107.6, 94.7, 61.7, 55.7, 30.9, 21.4, 14.2; Anal. Calcd for $C_{19}H_{20}N_2O_3$: C, 70.35; H, 6.21; N, 8.64%; Found: C, 70.56; H, 6.27; N, 8.52%.

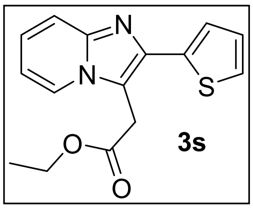

Ethyl 2-(2-(thiophen-2-yl)imidazo[1,2-*a*]pyridin-3-yl)acetate (**3s**):[2b] Yellow gummy mass (41.5 mg, 58%); ^{1}H NMR (CDCl$_3$, 400 MHz): δ 8.11 (d, J = 7.2 Hz, 1H), 7.64 (d, J = 7.2 Hz, 1H), 7.53-7.52 (m, 1H), 7.41-7.38 (m, 1H), 7.24-7.20 (m, 1H), 7.15-7.13 (m, 1H), 6.90-6.81 (m, 1H), 4.19 (q, J = 7.2 Hz, 2H), 4.11 (s, 2H), 1.25 (t, J = 7.2 Hz, 3H); ^{13}C{^{1}H} NMR (CDCl$_3$, 100 MHz): δ 169.1, 145.1, 137.2, 127.9, 126.5, 126.1, 125.4, 124.9, 123.7, 117.5, 112.6, 112.4, 61.8, 30.9, 14.2.

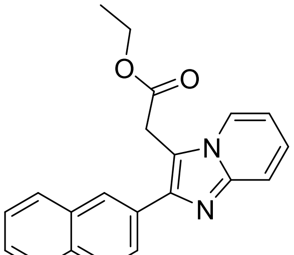

3t

Ethyl 2-(2-(naphthalen-2-yl)imidazo[1,2-*a*]pyridin-3-yl)acetate (**3t**): Yellow gummy mass (66 mg, 80%); ^{1}H NMR ($CDCl_3$, 400 MHz): δ 8.33 (s, 1H), 8.17 (d, J = 6.8 Hz, 1H), 8.02-8.00 (m, 1H), 7.96-7.86 (m, 3H), 7.70 (d, J = 9.2 Hz, 1H), 7.51-7.48 (m, 2H), 7.26-7.22 (m, 1H), 6.89-6.86 (m, 1H), 4.25 (q, J = 7.2 Hz, 2H), 4.10 (s, 2H), 1.32 (t, J = 7.2 Hz, 3H); ^{13}C{^{1}H} NMR ($CDCl_3$, 100 MHz): δ 169.5, 145.2, 144.6, 133.5, 133.0, 131.5, 128.45, 128.40, 127.8, 127.7, 126.6, 126.3, 126.2, 124.7, 123.8, 117.6, 113.3, 112.5, 61.8, 31.0, 14.3; Anal. Calcd for $C_{21}H_{18}N_2O_2$: C, 76.34; H, 5.49; N, 8.48%; Found: C, 76.52; H, 5.43; N, 8.59%.

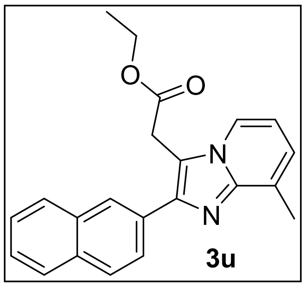

3u

Ethyl 2-(8-methyl-2-(naphthalen-2-yl)imidazo[1,2-*a*]pyridin-3-yl)acetate (**3u**): Yellow gummy mass (61 mg, 71%); ^{1}H NMR ($CDCl_3$, 400 MHz): δ 8.31 (s, 1H), 8.04-8.00 (m, 2H), 7.96-7.91 (m, 2H), 7.89-7.86 (m, 1H), 7.51-7.49 (m, 2H), 7.05 (d, J = 6.8 Hz, 1H), 6.81 (t, J = 6.8 Hz, 1H), 4.25 (q, J = 7.2 Hz, 2H); 4.08 (s, 2H), 2.70 (s, 3H), 1.32 (t, J = 7.2 Hz, 3H); ^{13}C{^{1}H} NMR ($CDCl_3$, 100 MHz): δ 169.6, 145.8, 144.2, 133.6, 133.0, 131.8, 128.5, 128.3, 127.88, 127.84, 127.7, 126.9, 126.2, 126.1, 123.5, 121.7, 113.8, 112.6, 61.7, 31.2, 17.3, 14.3; HRMS (ESI-TOF) m/z: [M + H$^+$] Calcd for $C_{22}H_{21}N_2O_2$: 345.1598; Found 345.1598.

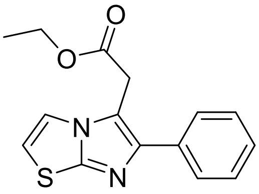

5a

Ethyl 2-(6-phenylimidazo[2,1-*b*]thiazol-5-yl)acetate (**5a**):[2b] Yellow gummy mass (47 mg, 66%); ^{1}H NMR (CDCl$_3$, 400 MHz): δ 7.73-7.71 (m, 2H), 7.49 (d, J = 4.4 Hz, 1H), 7.45-7.41 (m, 2H), 7.32 (t, J = 7.2 Hz, 1H), 6.82 (d, J = 4.4 Hz, 1H), 4.20 (q, J = 8.0 Hz, 2H), 3.92 (s, 2H), 1.27 (t, J = 7.2 Hz, 3H); ^{13}C{^{1}H} NMR (CDCl$_3$, 100 MHz): δ 169.5, 149.2, 145.5, 134.2, 128.6, 127.9, 127.5, 118.0, 114.5, 112.3, 61.7, 31.6, 14.2.

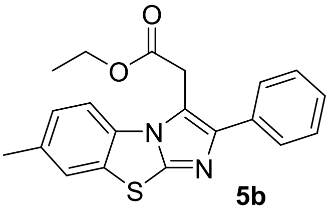

5b

Ethyl 2-(7-methyl-2-phenylbenzo[*d*]imidazo[2,1-*b*]thiazol-3-yl)acetate (**5b**): Yellow solid (64 mg, 73%); M.p. 137-138 ^{o}C; ^{1}H NMR (CDCl$_3$, 400 MHz): δ 7.73 (d, J = 7.2 Hz, 2H), 7.65 (d, J = 8.4 Hz, 1H), 7.50 (s, 1H), 7.45 (t, J = 7.6 Hz, 2H), 7.35 (t, J = 7.2 Hz, 1H), 7.21 (d, J = 8.4 Hz, 1H), 4.25 (q, J = 7.2 Hz, 2H), 4.18 (s, 2H), 2.45 (s, 3H), 1.27 (t, J = 7.2 Hz, 3H); ^{13}C{^{1}H} NMR (CDCl$_3$, 100 MHz): δ 170.1, 147.5, 145.9, 134.8, 134.1, 131.1, 130.6, 128.7, 128.1, 127.7, 127.1, 124.6, 116.8, 112.7, 61.8, 31.9, 21.3, 14.3; HRMS (ESI-TOF) m/z: [M + H$^+$] Calcd for $C_{20}H_{19}N_2O_2S$: 351.1162; Found 351.1163.

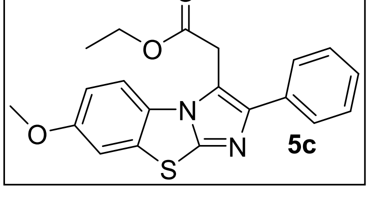

5c

Ethyl 2-(7-methoxy-2-phenylbenzo[*d*]imidazo[2,1-*b*]thiazol-3-yl)acetate (**5c**): White solid (71 mg, 78%); M.p. 140-141 °C; ^{1}H NMR (CDCl$_3$, 400 MHz): δ 7.73 (d, J = 7.2 Hz, 2H), 7.67 (d, J = 8.8 Hz, 1H), 7.44 (t, J = 8.0 Hz, 2H), 7.36-7.32 (m, 1H), 7.19 (d, J = 2.8 Hz, 1H), 6.97-6.95 (m, 1H), 4.25 (q, J = 7.2 Hz, 2H), 4.15 (s, 2H), 3.85 (s, 3H), 1.26 (t, J = 7.2 Hz, 3H); ^{13}C{^{1}H} NMR (CDCl$_3$, 100 MHz):δ 170.0, 156.9, 146.9, 145.6, 134.1, 131.8, 128.6, 128.0, 127.6, 127.3, 116.7, 113.6, 113.1, 108.8, 61.8, 55.9, 31.7, 14.2; HRMS (ESI-TOF) m/z: [M + H$^+$] Calcd for $C_{20}H_{19}N_2O_3S$: 367.1110; Found 367.1110.

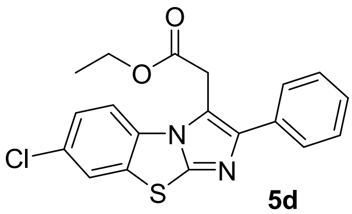

5d

Ethyl 2-(7-chloro-2-phenylbenzo[*d*]imidazo[2,1-*b*]thiazol-3-yl)acetate (**5d**): Yellow solid (65 mg, 71%); M.p. 141-142 °C; ^{1}H NMR (CDCl$_3$, 400 MHz): δ 7.73 (d, J = 8.4 Hz, 3H), 7.69 (d, J = 2.0 Hz, 1H), 7.46 (t, J = 8.0 Hz, 2H), 7.41-7.35 (m, 2H), 4.26 (q, J = 7.2 Hz, 2H), 4.17 (s, 2H), 1.28 (t, J = 7.2 Hz, 3H); ^{13}C{^{1}H} NMR (CDCl$_3$, 100 MHz): δ 169.8, 147.3, 146.5, 133.7, 132.1, 131.7, 130.2, 128.7, 128.1, 127.9, 126.4, 124.2, 117.0, 113.8, 62.0, 31.7, 14.3; Anal. Calcd for $C_{19}H_{15}ClN_2O_2S$: C, 61.54; H, 4.08; N, 7.55%; Found C, 61.36; H, 4.01; N, 7.63%.

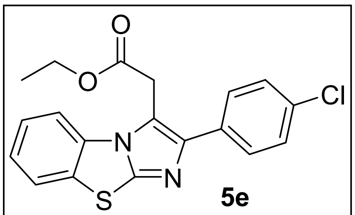

5e

Ethyl 2-(2-(4-chlorophenyl)benzo[*d*]imidazo[2,1-*b*]thiazol-3-yl)acetate (**5e**): Yellow solid (67 mg, 72%); M.p. 90-91 °C; ^{1}H NMR (CDCl$_3$, 400 MHz): δ 7.81 (d, J = 8.0 Hz, 1H), 7.72-7.68 (m, 3H), 7.45-7.41 (m, 3H), 7.36-7.32 (m, 1H), 4.26 (q, J = 7.2 Hz, 2H), 4.16 (s, 2H), 1.27 (t, J = 7.2 Hz, 3H); ^{13}C{^{1}H} NMR (CDCl$_3$, 100 MHz): δ 169.8, 147.7, 145.1, 133.7,

133.1, 132.5, 130.5, 129.3, 128.9, 126.2, 124.8, 124.5, 117.1, 113.1, 62.0, 31.8, 14.2; Anal. Calcd for $C_{19}H_{15}ClN_2O_2S$: C, 61.54; H, 4.08; N, 7.55%; Found C, 61.74; H, 4.04; N, 7.64%.

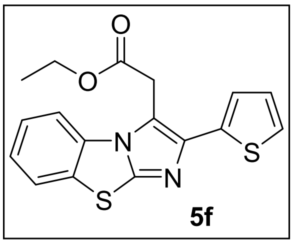

Ethyl 2-(2-(thiophen-2-yl)benzo[*d*]imidazo[2,1-*b*]thiazol-3-yl)acetate (**5f**): Yellow solid (67 mg, 79%); M.p. 91-92 °C; ^{1}H NMR ($CDCl_3$, 400 MHz): δ 7.83 (d, *J* = 8.0 Hz, 1H), 7.69 (d, *J* = 7.6 Hz, 1H), 7.45-7.40 (m, 2H), 7.35-7.31 (m, 2H), 7.12-7.10 (m, 1H), 4.26-4.21 (m, 4H), 1.25 (t, *J* = 7.2 Hz, 3H); $^{13}C\{^1H\}$ NMR ($CDCl_3$, 100 MHz): δ 169.5, 147.8, 140.5, 136.9, 133.0, 130.5, 127.7, 126.2, 125.4, 124.9, 124.8, 124.4, 116.3, 113.1, 61.9, 31.9, 14.2; Anal. Calcd for $C_{17}H_{14}N_2O_2S_2$: C, 59.63; H, 4.12; N, 8.18%; Found C, 59.47; H, 4.15; N, 8.07%.

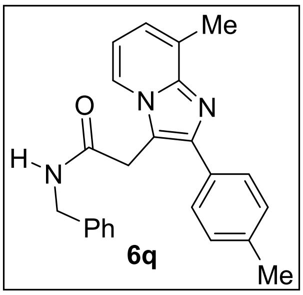

***N*-Benzyl-2-(8-methyl-2-(*p*-tolyl)imidazo[1,2-*a*]pyridin-3-yl)acetamide** (**6q**): Brown gummy (52 mg, 56%); ^{1}H NMR ($CDCl_3$, 400 MHz): δ 7.86 (d, *J* = 6.8 Hz, 1H), 7.62-7.59 (m, 2H), 7.24-7.19 (m, 4H), 7.08-7.03 (m, 4H), 6.78 (t, *J* = 6.8 Hz, 1H), 5.98 (s, 1H), 4.37 (d, *J* = 5.6 Hz, 2H), 4.03 (s, 2H), 2.65 (s, 3H), 2.39 (s, 3H); $^{13}C\{^1H\}$ NMR ($CDCl_3$, 100 MHz): δ 168.5, 145.4, 144.5, 138.1, 137.8, 130.9, 129.6, 128.7, 128.4, 127.7, 127.66, 127.60, 124.0, 121.2, 113.5, 113.0, 43.8, 32.8, 21.4, 17.2; Anal. Calcd for $C_{24}H_{23}N_3O$: C, 78.02; H, 6.27; N, 11.37%; Found C, 78.21; H, 6.32; N, 11.30%.

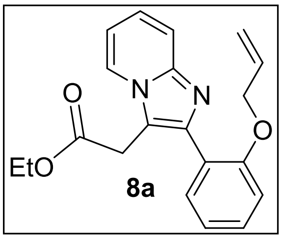

Ethyl 2-(2-(2-(allyloxy)phenyl)imidazo[1,2-*a*]pyridin-3-yl)acetate (8a): Yellow Gummy (29 mg, 35%); ^{1}H NMR ($CDCl_3$, 400 MHz): δ 7.98 (d, *J* = 6.8 Hz, 1H), 7.67-7.64 (m, 2H), 7.36-7.32 (m, 1H), 7.26-7.18 (m, 1H), 7.08 (t, *J* = 7.2 Hz, 1H), 7.00-6.98 (m, 1H), 6.84 (t, *J* = 6.8 Hz, 1H), 5.98-5.88 (m, 1H), 5.29-5.24 (m, 1H), 5.19-5.16 (m, 1H), 4.52 (d, *J* = 2.8 Hz, 2H), 4.12 (q, *J* = 7.2 Hz, 2H), 3.93 (s, 2H), 1.20 (t, J = 7.2 Hz, 3H); $^{13}C\{^1H\}$ NMR ($CDCl_3$, 100 MHz): δ 169.7, 155.8, 145.1, 141.4, 133.6, 132.5, 129.5, 140.0, 123.9, 123.6, 121.4, 117.7, 117.4, 115.1, 113.2, 112.1, 69.9, 61.3, 30.9, 14.2; Anal. Calcd for $C_{20}H_{20}N_2O_3$: C, 71.41; H, 5.99; N, 8.33%; Found C, 71.24; H, 6.03; N, 8.41%.

7. NMR spectra [^{1}H and ^{13}C{^{1}H})] of synthesized products:

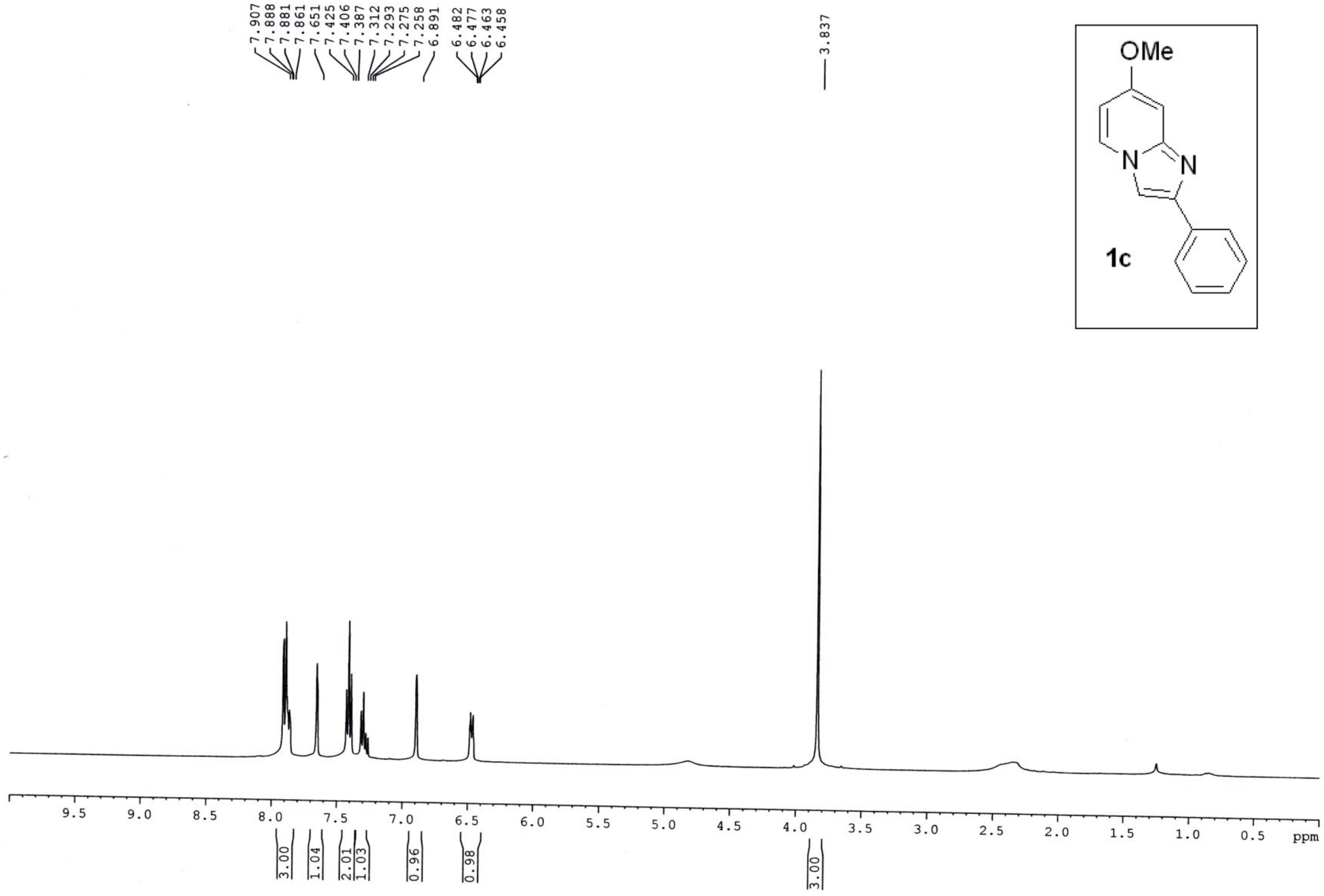
7.907
7.888
7.881
7.861
7.651
7.425
7.406
7.387
7.312
7.293
7.275
7.258
6.891
6.482
6.477
6.463
6.458
3.837
OMe
N
N
1c
9.5
9.0
8.5
8.0
7.5
7.0
6.5
6.0
5.5
5.0
4.5
4.0
3.5
3.0
2.5
2.0
1.5
1.0
0.5
ppm
3.00
1.04
2.01
1.03
0.96
0.98
3.00

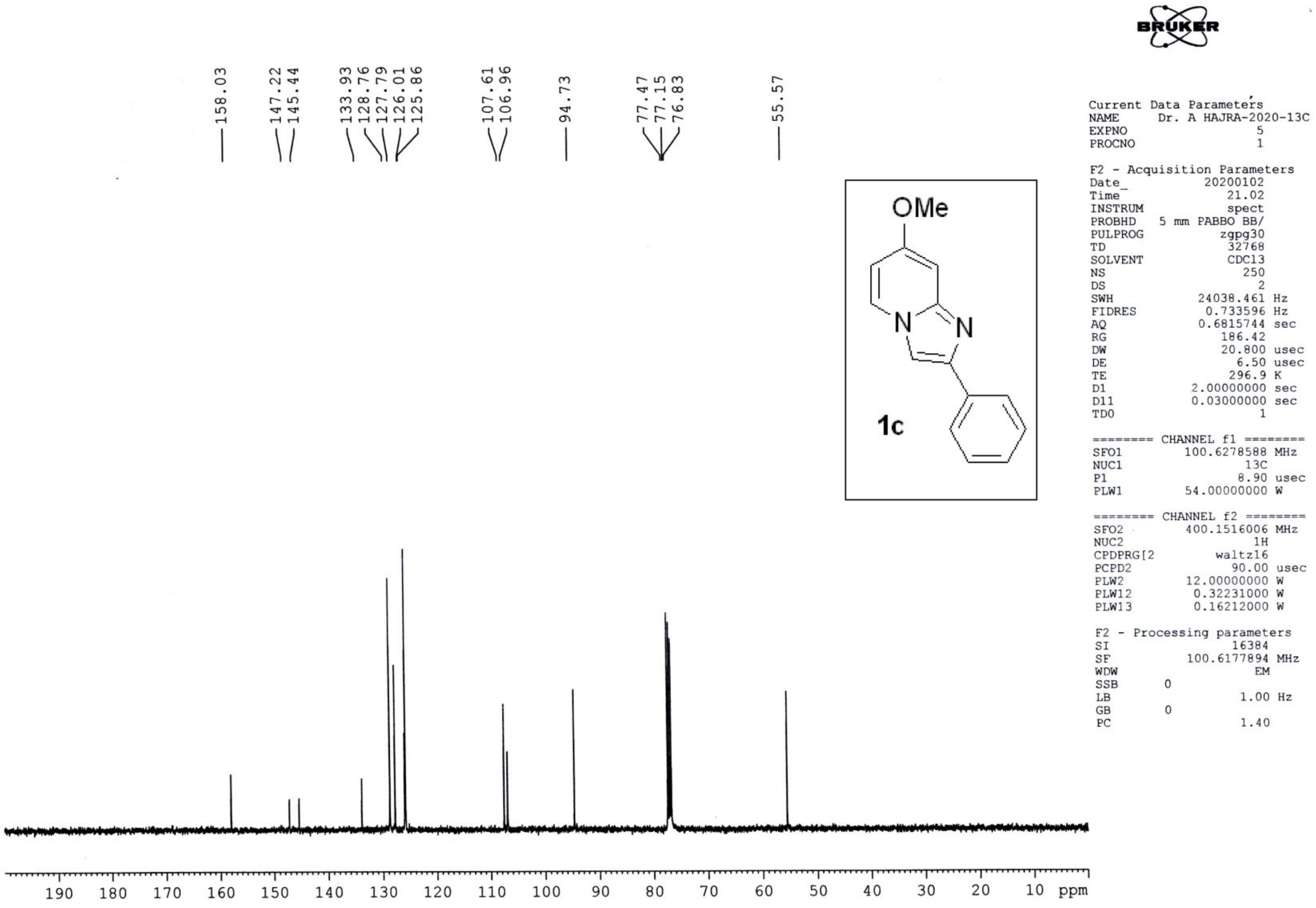
158.03
147.22
145.44
133.93
128.76
127.79
126.01
125.86
107.61
106.96
94.73
77.47
77.15
76.83
55.57
OMe
N
N
1c
190 180 170 160 150 140 130 120 110 100 90 80 70 60 50 40 30 20 10 ppm
BRUKER
Current Data Parameters
NAME Dr. A HAJRA-2020-13C
EXPNO 5
PROCNO 1
F2 - Acquisition Parameters
Date_ 20200102
Time 21.02
INSTRUM spect
PROBHD 5 mm PABBO BB/
PULPROG zgpg30
TD 32768
SOLVENT CDCl3
NS 250
DS 2
SWH 24038.461 Hz
FIDRES 0.733596 Hz
AQ 0.6815744 sec
RG 186.42
DW 20.800 usec
DE 6.50 usec
TE 296.9 K
D1 2.00000000 sec
D11 0.03000000 sec
TD0 1
======== CHANNEL f1 ========
SFO1 100.6278588 MHz
NUC1 13C
P1 8.90 usec
PLW1 54.00000000 W
======== CHANNEL f2 ========
SFO2 400.1516006 MHz
NUC2 1H
CPDPRG[2 waltz16
PCPD2 90.00 usec
PLW2 12.00000000 W
PLW12 0.32231000 W
PLW13 0.16212000 W
F2 - Processing parameters
SI 16384
SF 100.6177894 MHz
WDW EM
SSB 0
LB 1.00 Hz
GB 0
PC 1.40

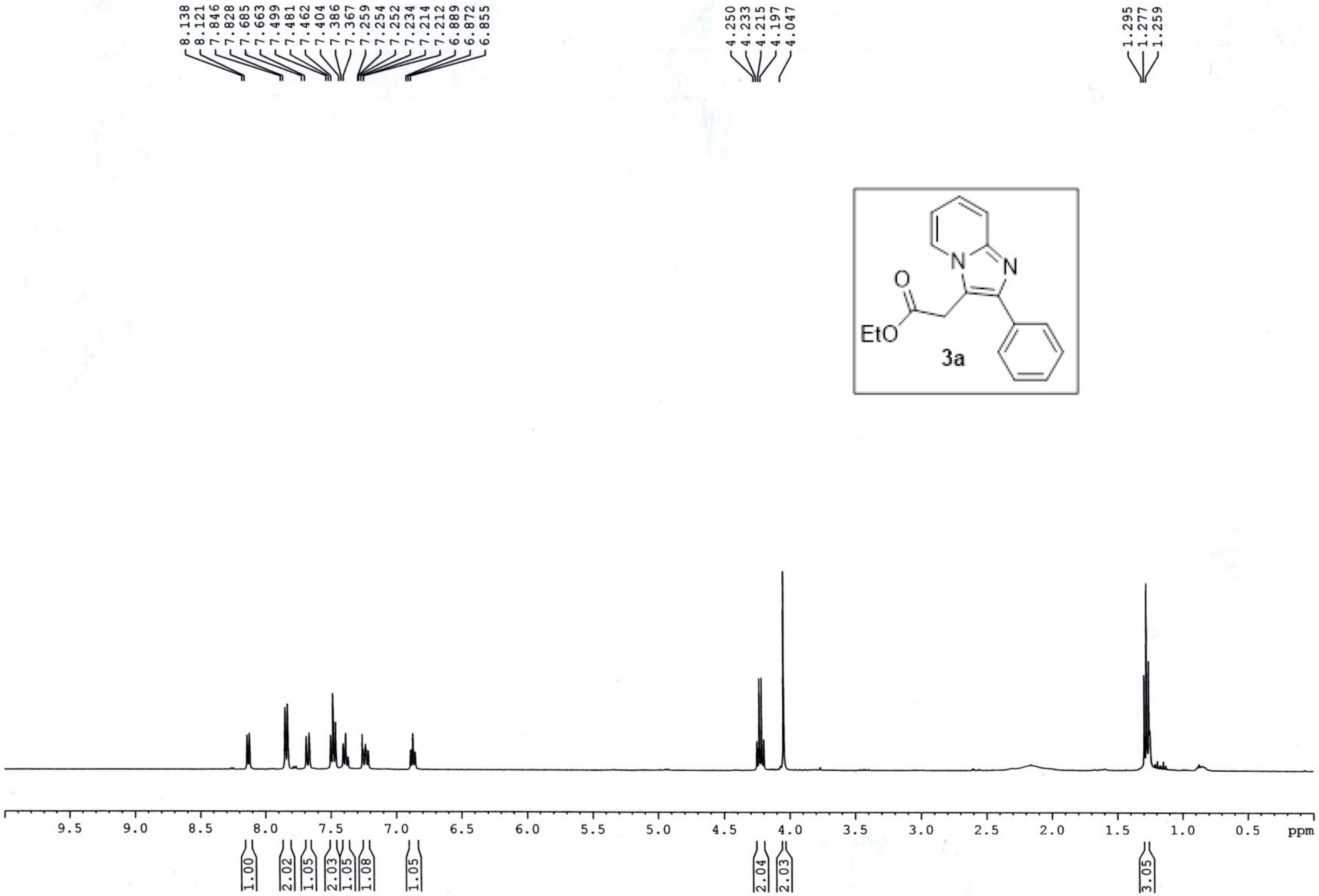
8.138
8.121
7.846
7.828
7.685
7.663
7.499
7.481
7.462
7.404
7.386
7.367
7.259
7.254
7.252
7.234
7.214
7.212
6.889
6.872
6.855
4.250
4.233
4.215
4.197
4.047
1.295
1.277
1.259
O
N
N
EtO
3a
9.5 9.0 8.5 8.0 7.5 7.0 6.5 6.0 5.5 5.0 4.5 4.0 3.5 3.0 2.5 2.0 1.5 1.0 0.5 ppm
1.00
2.02
1.05
2.03
1.05
1.08
1.05
2.04
2.03
3.05

13C of VBSB 22/3

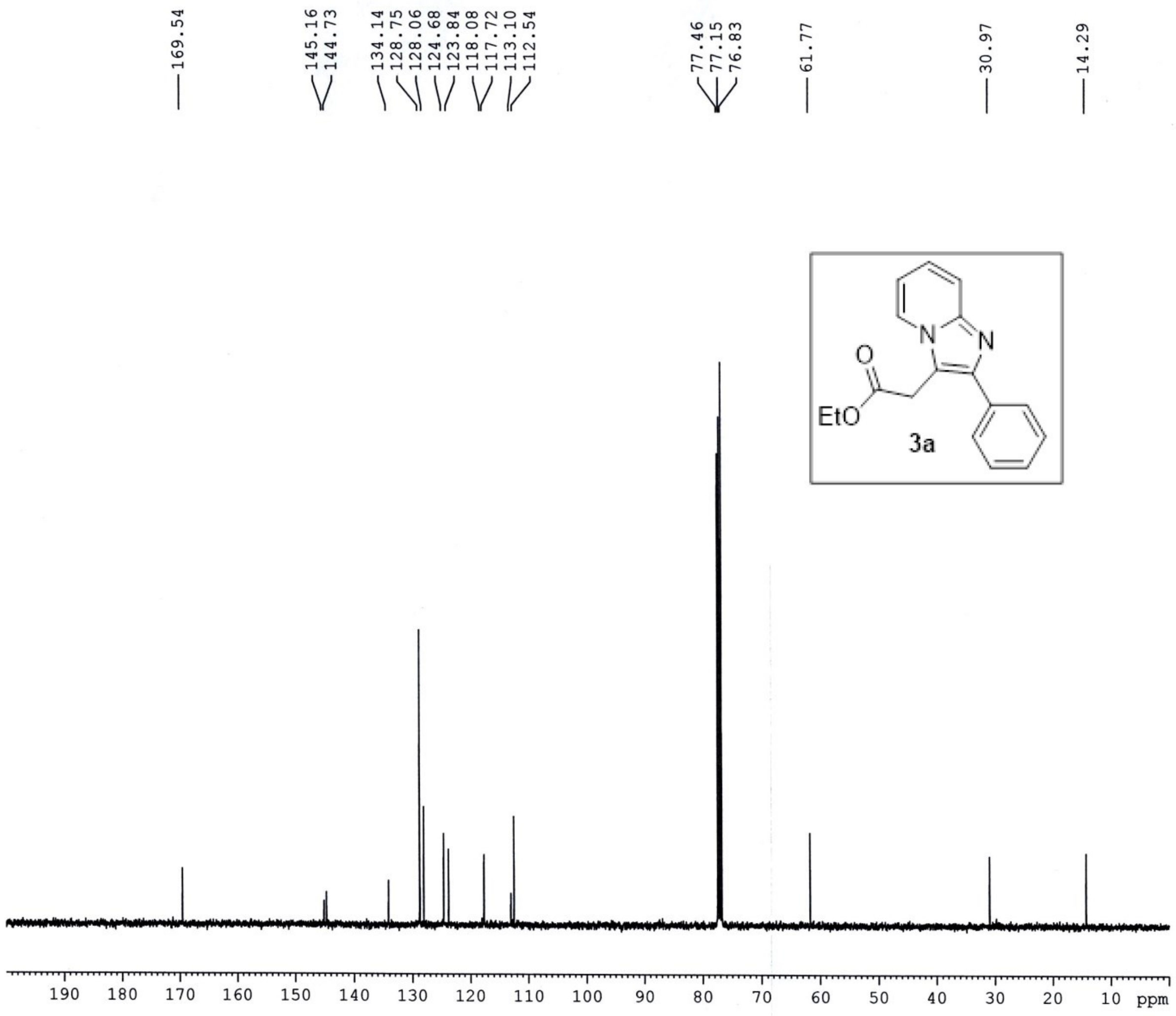


```
Current Data Parameters
NAME     Dr. A HAJRA-2019-13C
EXPNO                    360
PROCNO                     1

F2 - Acquisition Parameters
Date_               20190910
Time                   11.32
INSTRUM                spect
PROBHD   5 mm PABBO BB/
PULPROG              zgpg30
TD                     32768
SOLVENT                CDCl3
NS                       512
DS                         2
SWH                24038.461 Hz
FIDRES              0.733596 Hz
AQ                 0.6815744 sec
RG                     135.7
DW                    20.800 usec
DE                      6.50 usec
TE                     298.7 K
D1                2.00000000 sec
D11               0.03000000 sec
TD0                        1

======== CHANNEL f1 ========
SFO1             100.6278588 MHz
NUC1                     13C
P1                      8.90 usec
PLW1             54.00000000 W

======== CHANNEL f2 ========
SFO2             400.1516006 MHz
NUC2                      1H
CPDPRG[2             waltz16
PCPD2                  90.00 usec
PLW2             12.00000000 W
PLW12             0.32231000 W
PLW13             0.16212000 W

F2 - Processing parameters
SI                     16384
SF               100.6177857 MHz
WDW                       EM
SSB      0
LB                      1.00 Hz
GB       0
PC                      1.40
```

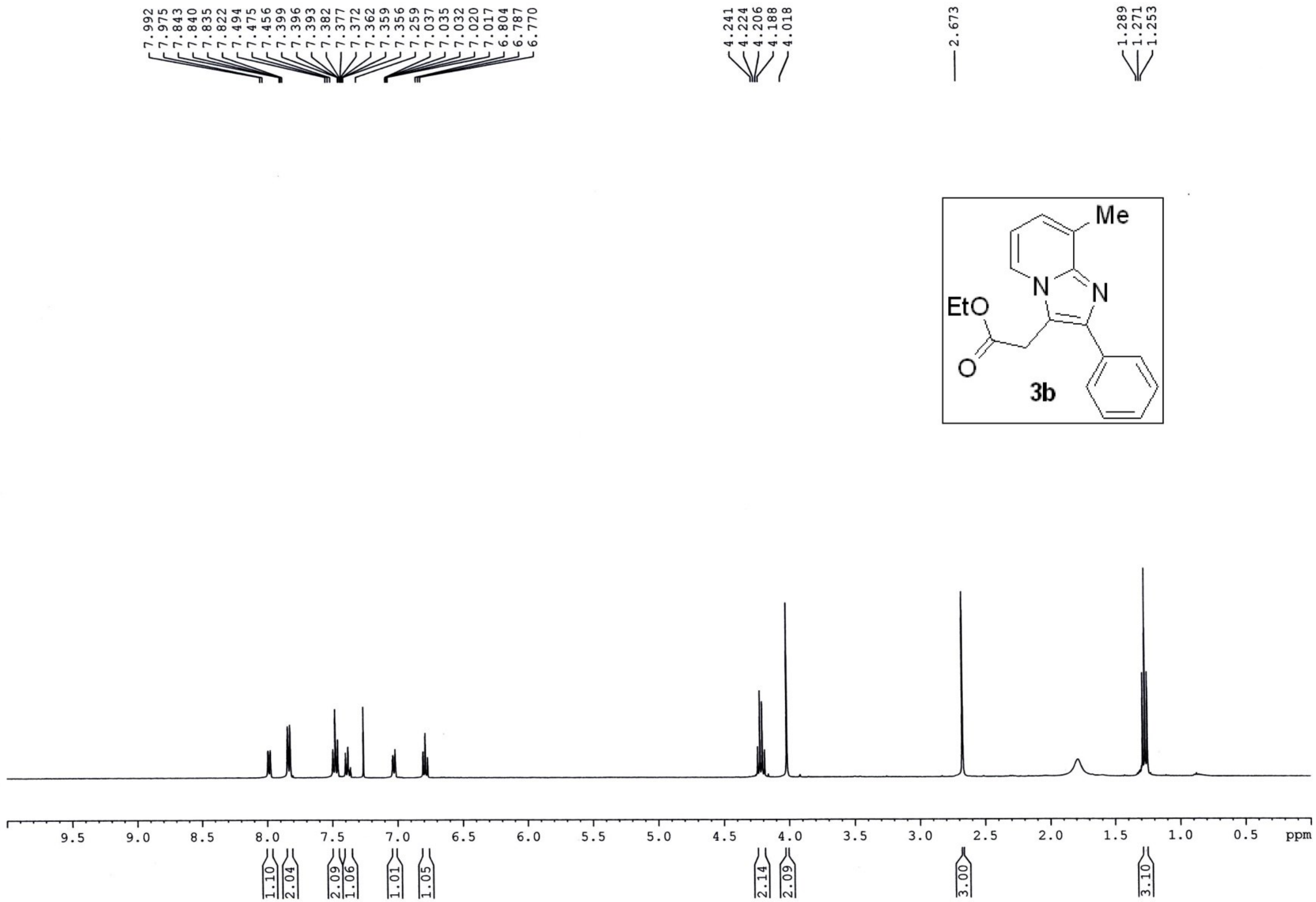
7.992
7.975
7.843
7.840
7.835
7.822
7.494
7.475
7.456
7.399
7.396
7.393
7.382
7.377
7.372
7.362
7.359
7.356
7.259
7.037
7.035
7.032
7.020
7.017
6.804
6.787
6.770
4.241
4.224
4.206
4.188
4.018
2.673
1.289
1.271
1.253
Me
N
N
EtO
O
3b
9.5 9.0 8.5 8.0 7.5 7.0 6.5 6.0 5.5 5.0 4.5 4.0 3.5 3.0 2.5 2.0 1.5 1.0 0.5 ppm
1.10
2.04
2.09
1.06
1.01
1.05
2.14
2.09
3.00
3.10

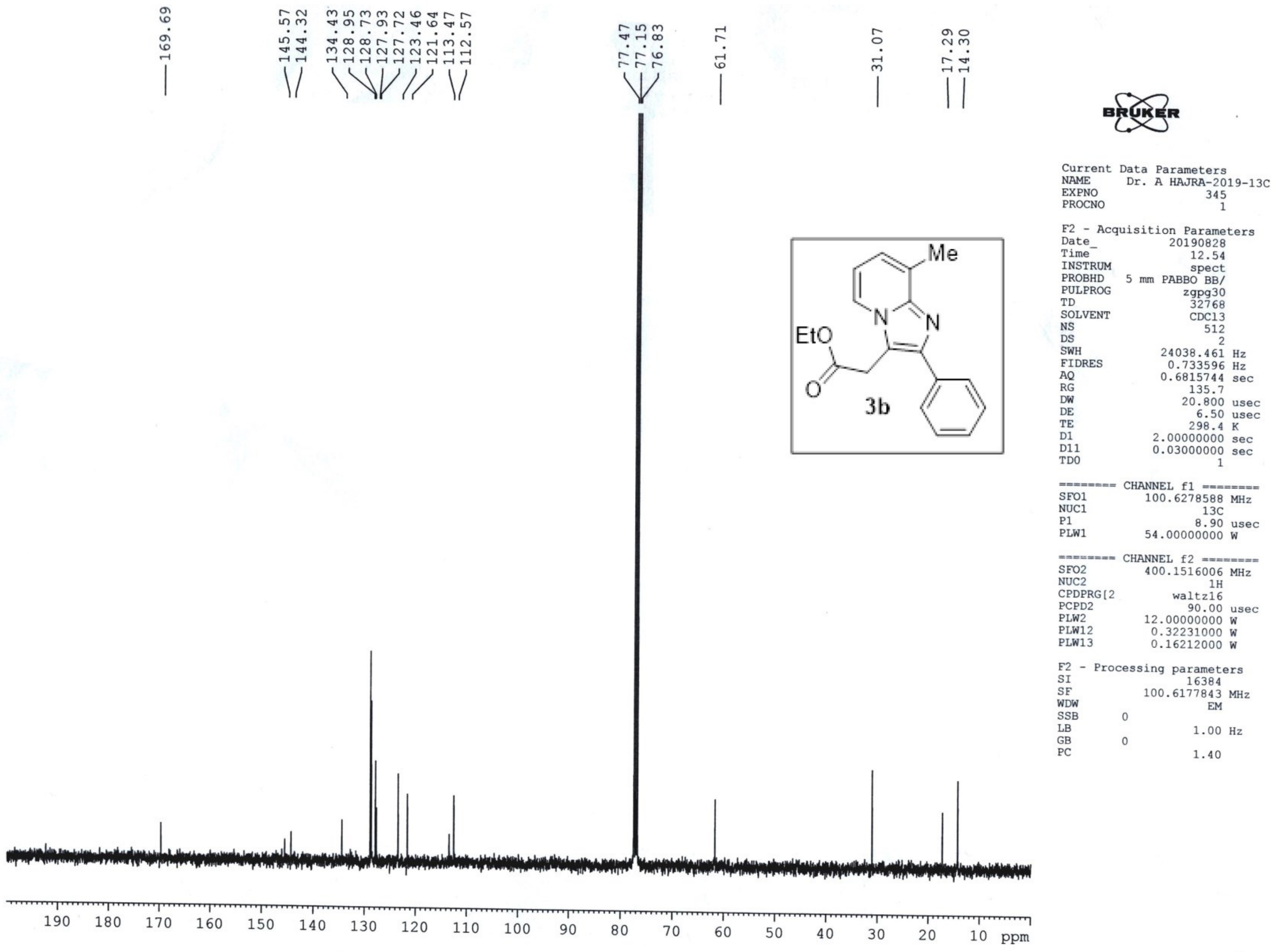
169.69
145.57
144.32
134.43
128.95
128.73
127.93
127.72
123.46
121.64
113.47
112.57
77.47
77.15
76.83
61.71
31.07
17.29
14.30
Me
N
N
EtO
O
3b
BRUKER
Current Data Parameters
NAME Dr. A HAJRA-2019-13C
EXPNO 345
PROCNO 1
F2 - Acquisition Parameters
Date_ 20190828
Time 12.54
INSTRUM spect
PROBHD 5 mm PABBO BB/
PULPROG zgpg30
TD 32768
SOLVENT CDCl3
NS 512
DS 2
SWH 24038.461 Hz
FIDRES 0.733596 Hz
AQ 0.6815744 sec
RG 135.7
DW 20.800 usec
DE 6.50 usec
TE 298.4 K
D1 2.00000000 sec
D11 0.03000000 sec
TD0 1
======== CHANNEL f1 ========
SFO1 100.6278588 MHz
NUC1 13C
P1 8.90 usec
PLW1 54.00000000 W
======== CHANNEL f2 ========
SFO2 400.1516006 MHz
NUC2 1H
CPDPRG[2 waltz16
PCPD2 90.00 usec
PLW2 12.00000000 W
PLW12 0.32231000 W
PLW13 0.16212000 W
F2 - Processing parameters
SI 16384
SF 100.6177843 MHz
WDW EM
SSB 0
LB 1.00 Hz
GB 0
PC 1.40
190 180 170 160 150 140 130 120 110 100 90 80 70 60 50 40 30 20 10 ppm

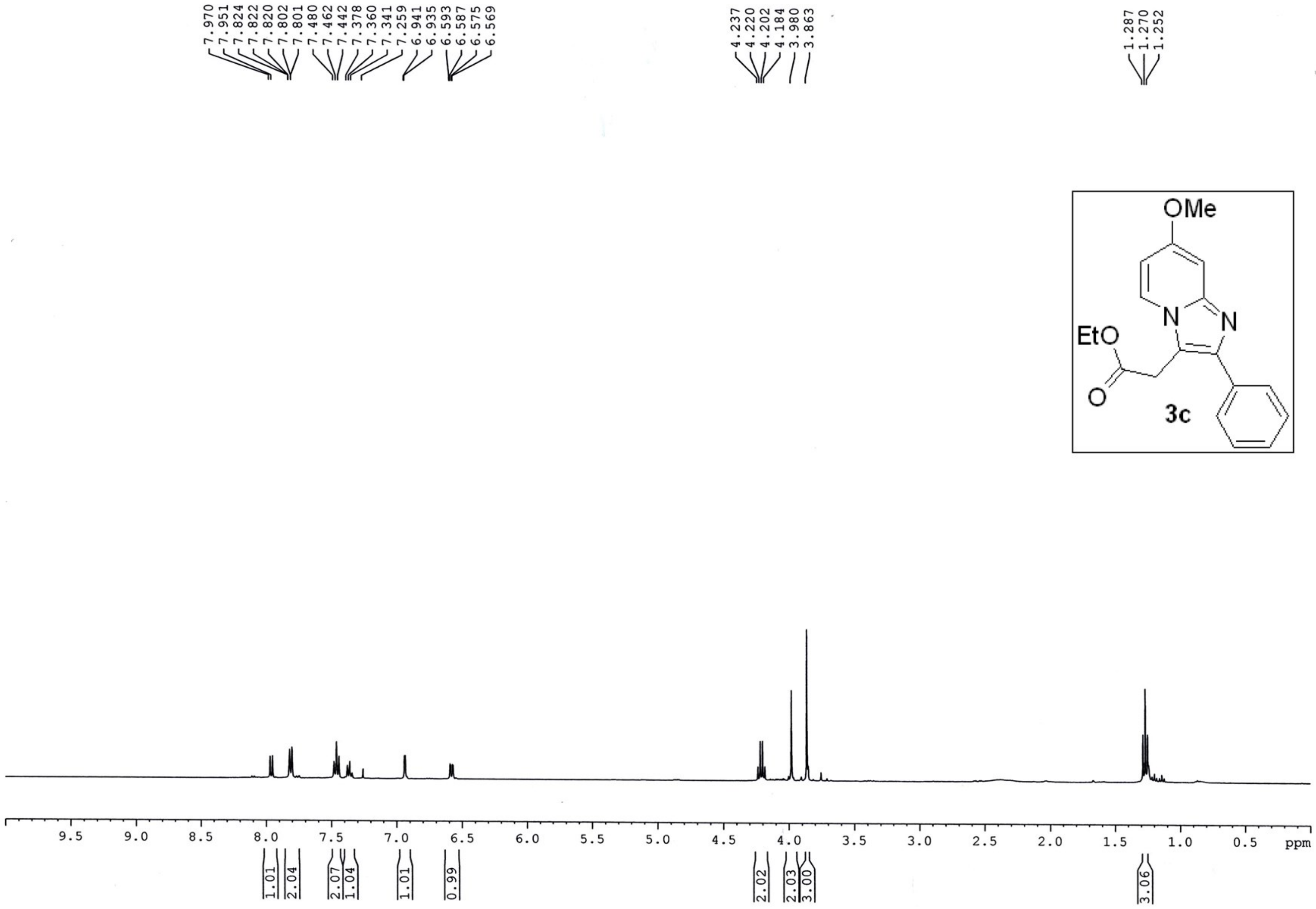
7.970
7.951
7.824
7.822
7.820
7.802
7.801
7.480
7.462
7.442
7.378
7.360
7.341
7.259
6.941
6.935
6.593
6.587
6.575
6.569
4.237
4.220
4.202
4.184
3.980
3.863
1.287
1.270
1.252
OMe
N
N
EtO
O
3c
9.5 9.0 8.5 8.0 7.5 7.0 6.5 6.0 5.5 5.0 4.5 4.0 3.5 3.0 2.5 2.0 1.5 1.0 0.5 ppm
1.01
2.04
2.07
1.04
1.01
0.99
2.02
2.03
3.00
3.06

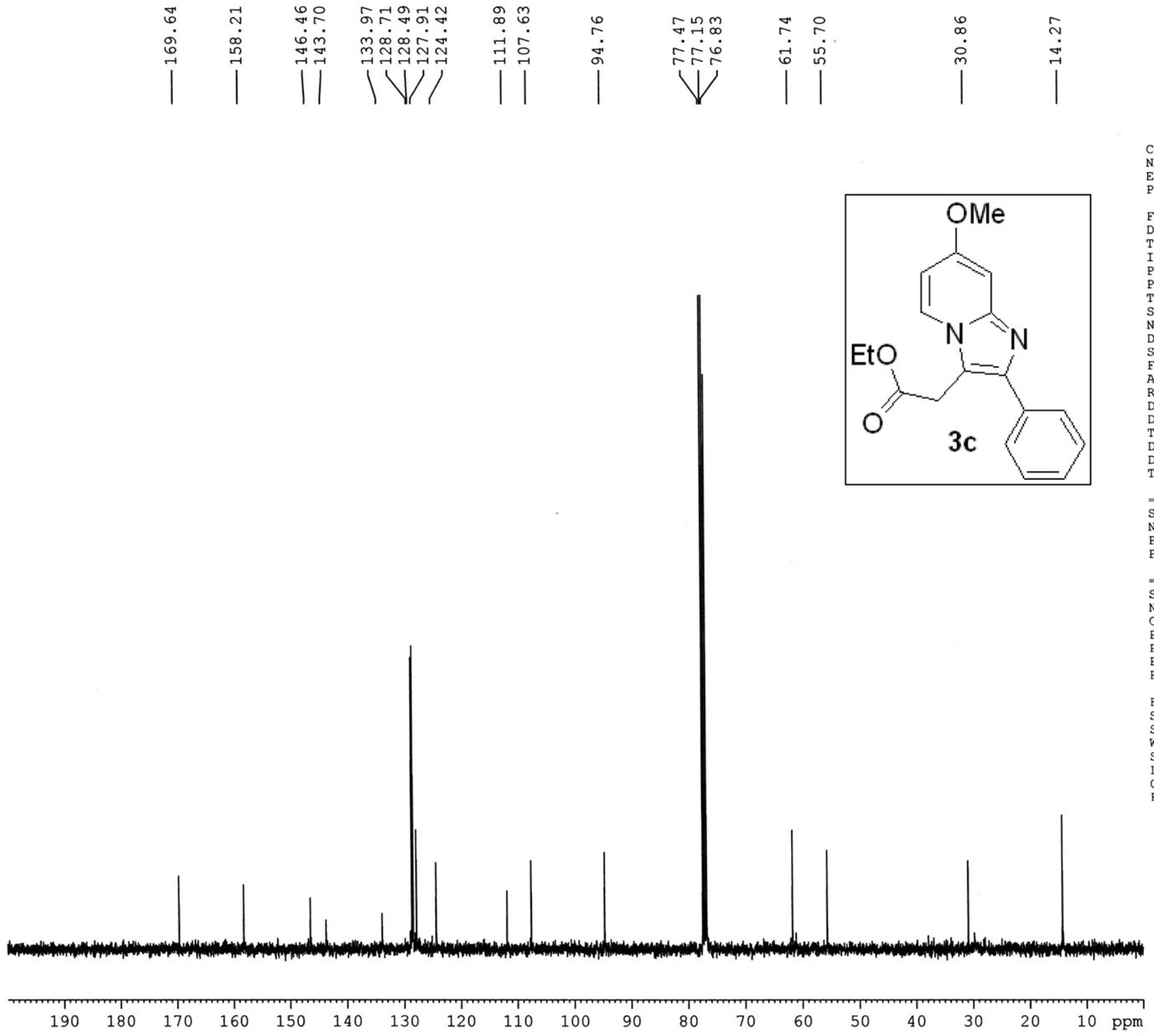


```
Current Data Parameters
NAME       Dr. A HAJRA-2020-13C
EXPNO                   67
PROCNO                   1

F2 - Acquisition Parameters
Date_             20200225
Time                 19.37
INSTRUM              spect
PROBHD   5 mm PABBO BB/
PULPROG            zgpg30
TD                  32768
SOLVENT             CDCl3
NS                    200
DS                      2
SWH             24038.461 Hz
FIDRES           0.733596 Hz
AQ              0.6815744 sec
RG                 186.42
DW                 20.800 usec
DE                   6.50 usec
TE                  296.3 K
D1             2.00000000 sec
D11            0.03000000 sec
TD0                     1

======== CHANNEL f1 ========
SFO1          100.6278588 MHz
NUC1                  13C
P1                   8.90 usec
PLW1          54.00000000 W

======== CHANNEL f2 ========
SFO2          400.1516006 MHz
NUC2                   1H
CPDPRG[2          waltz16
PCPD2               90.00 usec
PLW2          12.00000000 W
PLW12          0.32231000 W
PLW13          0.16212000 W

F2 - Processing parameters
SI                  16384
SF            100.6177874 MHz
WDW                    EM
SSB      0
LB                   1.00 Hz
GB       0
PC                   1.40
```

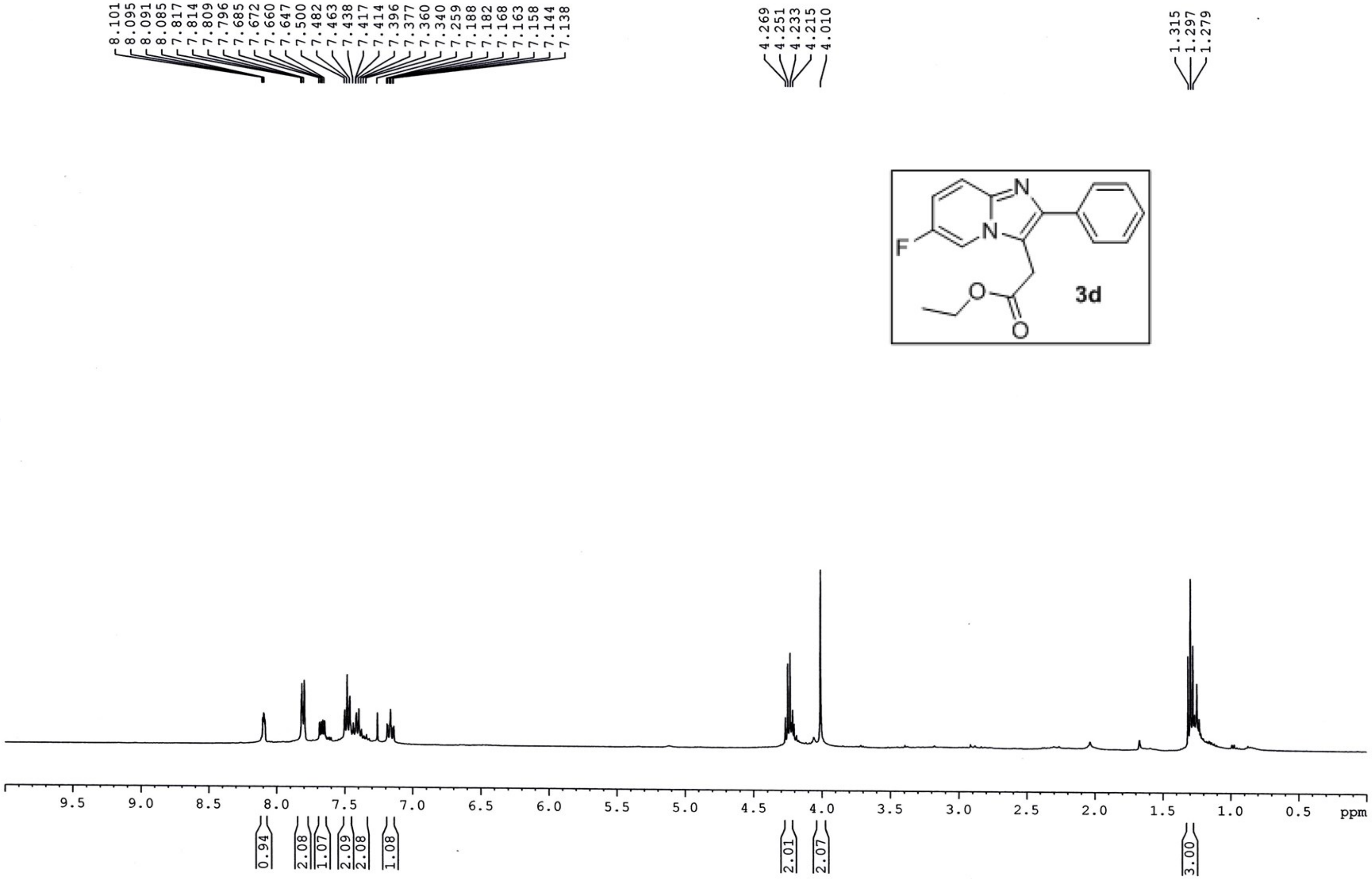
8.101
8.095
8.091
8.085
7.817
7.814
7.809
7.796
7.685
7.672
7.660
7.647
7.500
7.482
7.463
7.438
7.417
7.414
7.396
7.377
7.360
7.340
7.259
7.188
7.182
7.168
7.163
7.158
7.144
7.138
4.269
4.251
4.233
4.215
4.010
1.315
1.297
1.279
N
N
F
O
O
3d
9.5
9.0
8.5
8.0
7.5
7.0
6.5
6.0
5.5
5.0
4.5
4.0
3.5
3.0
2.5
2.0
1.5
1.0
0.5
ppm
0.94
2.08
1.07
2.09
2.08
1.08
2.01
2.07
3.00

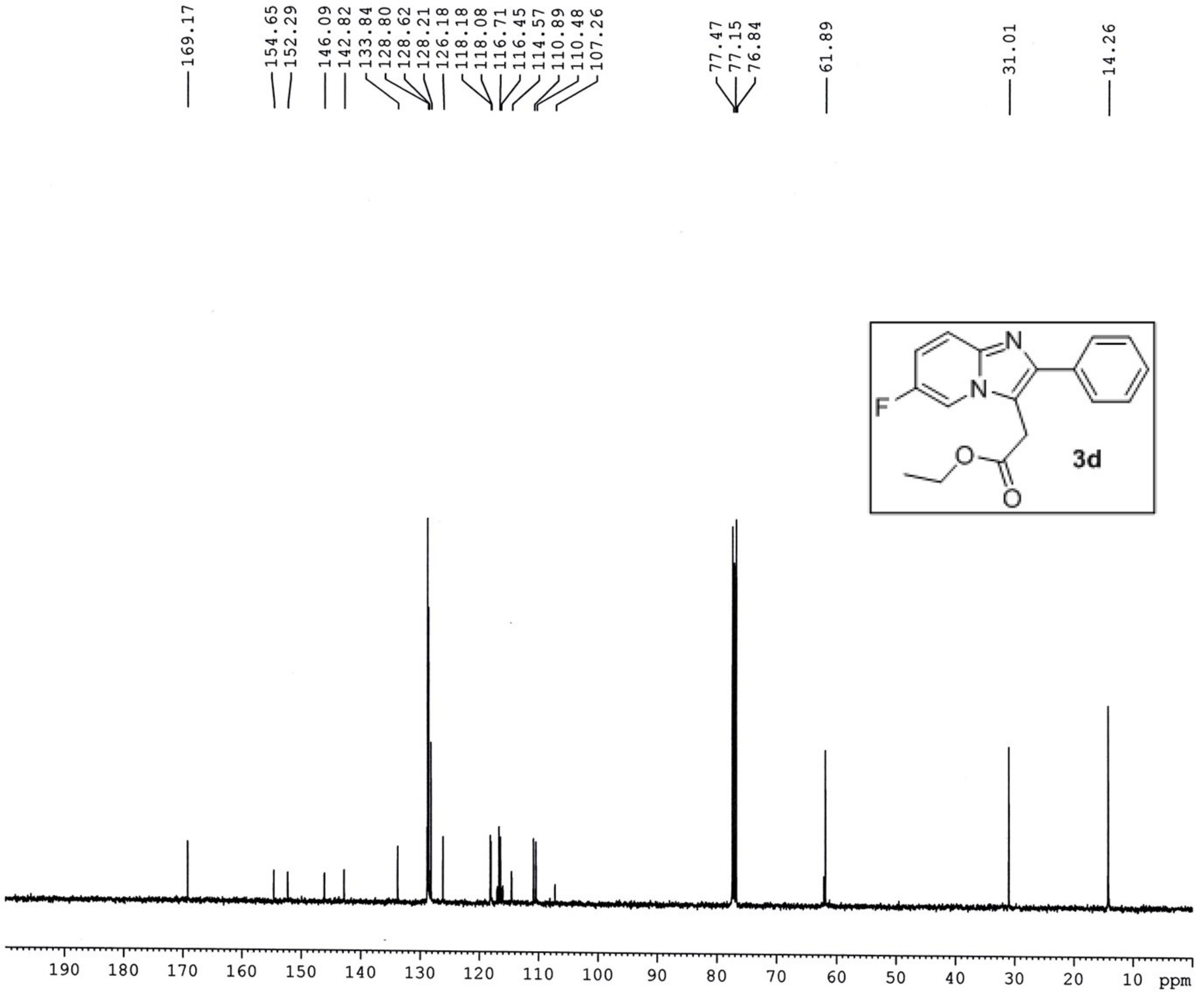
169.17
154.65
152.29
146.09
142.82
133.84
128.80
128.62
128.21
126.18
118.18
118.08
116.71
116.45
114.57
110.89
110.48
107.26
77.47
77.15
76.84
61.89
31.01
14.26
3d
190 180 170 160 150 140 130 120 110 100 90 80 70 60 50 40 30 20 10 ppm

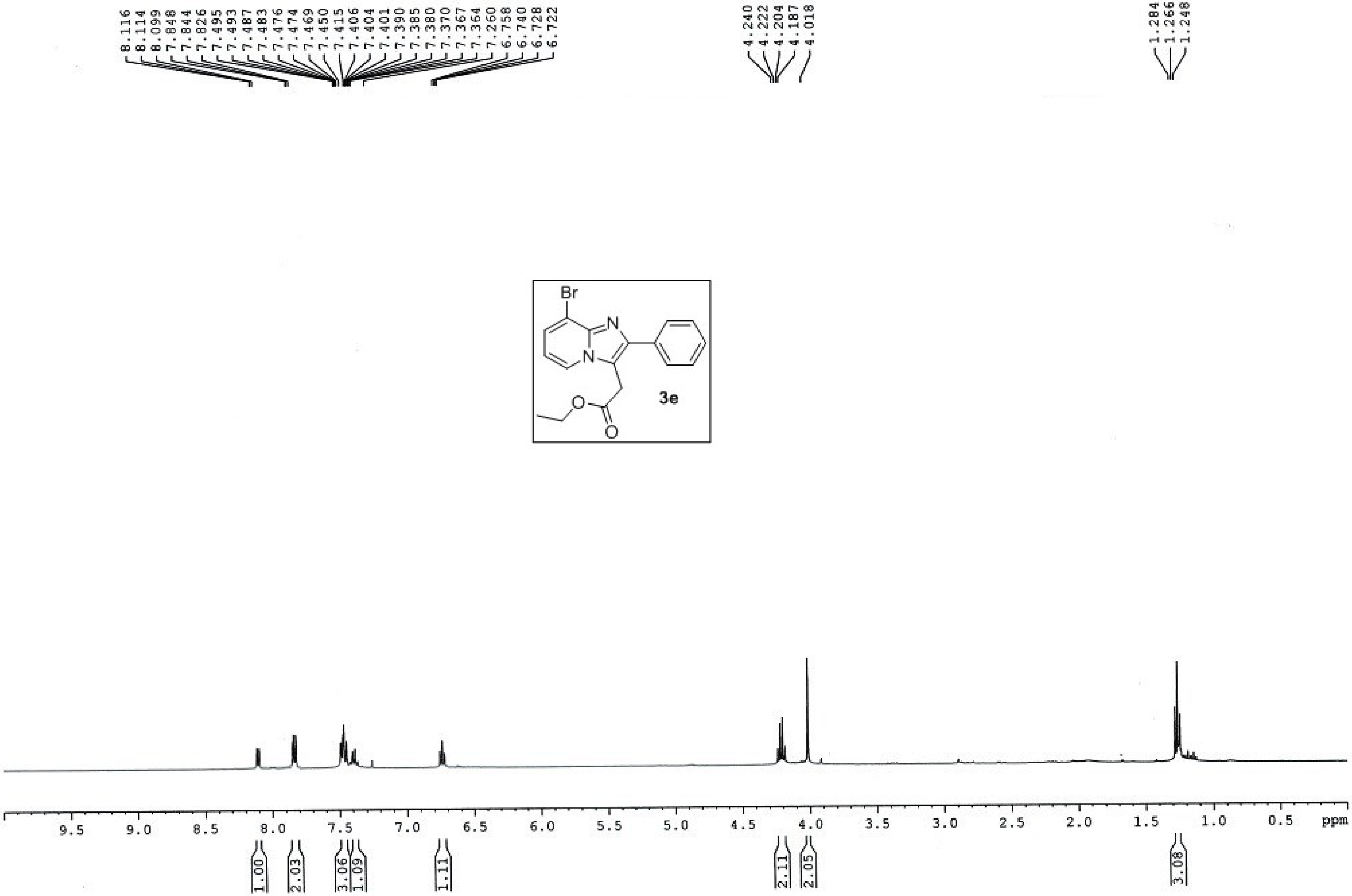
8.116 8.114 8.099 7.848 7.844 7.826 7.495 7.493 7.487 7.483 7.476 7.474 7.469 7.450 7.415 7.406 7.404 7.401 7.390 7.385 7.380 7.370 7.367 7.364 7.260 6.758 6.740 6.728 6.722
4.240 4.222 4.204 4.187 4.018
1.284 1.266 1.248
Br
N
N
O
O
3e
9.5 9.0 8.5 8.0 7.5 7.0 6.5 6.0 5.5 5.0 4.5 4.0 3.5 3.0 2.5 2.0 1.5 1.0 0.5 ppm
1.00 2.03 3.06 1.09 1.11 2.11 2.05 3.08

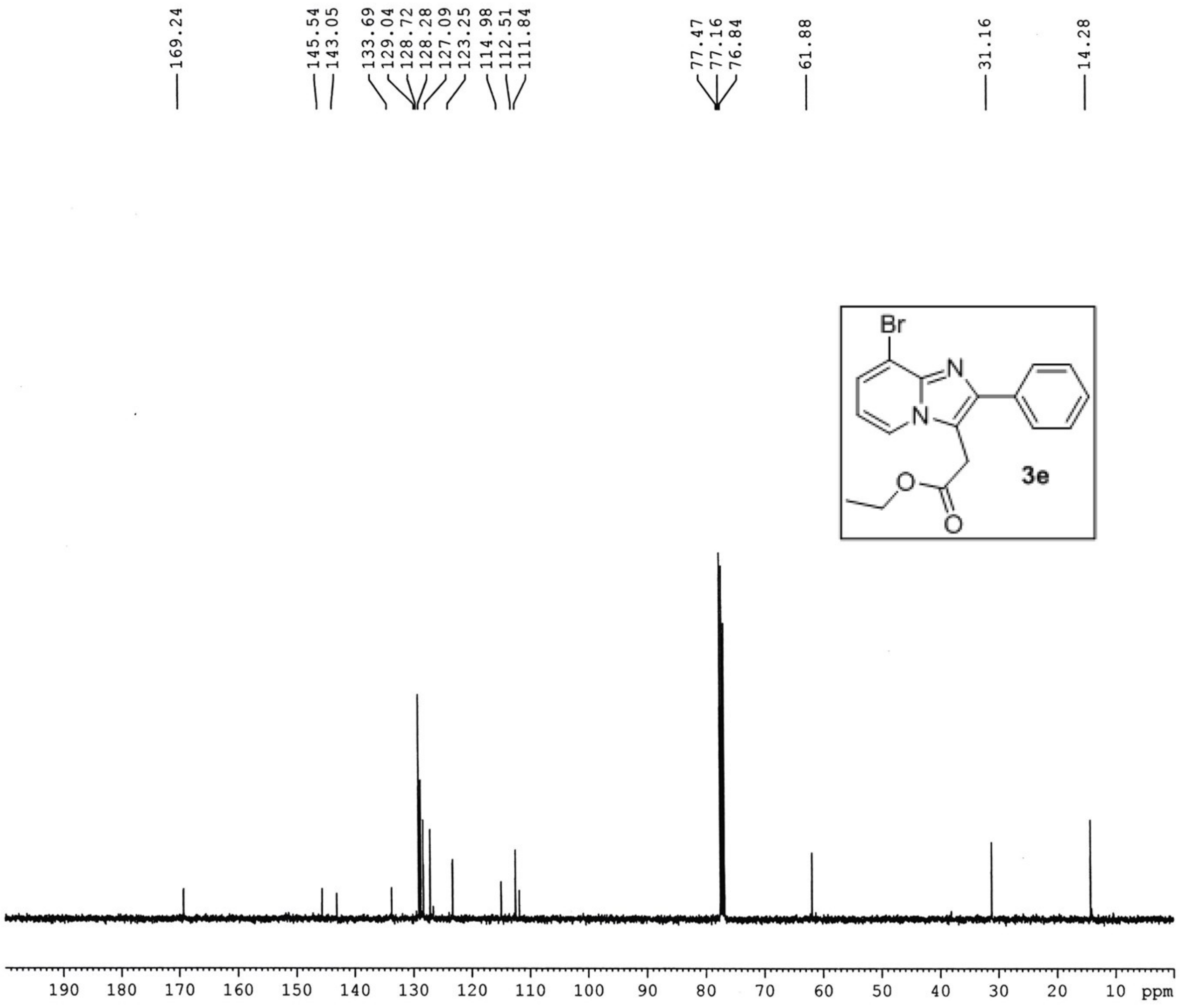
169.24
145.54
143.05
133.69
129.04
128.72
128.28
127.09
123.25
114.98
112.51
111.84
77.47
77.16
76.84
61.88
31.16
14.28
Br
N
N
O
O
3e
190 180 170 160 150 140 130 120 110 100 90 80 70 60 50 40 30 20 10 ppm

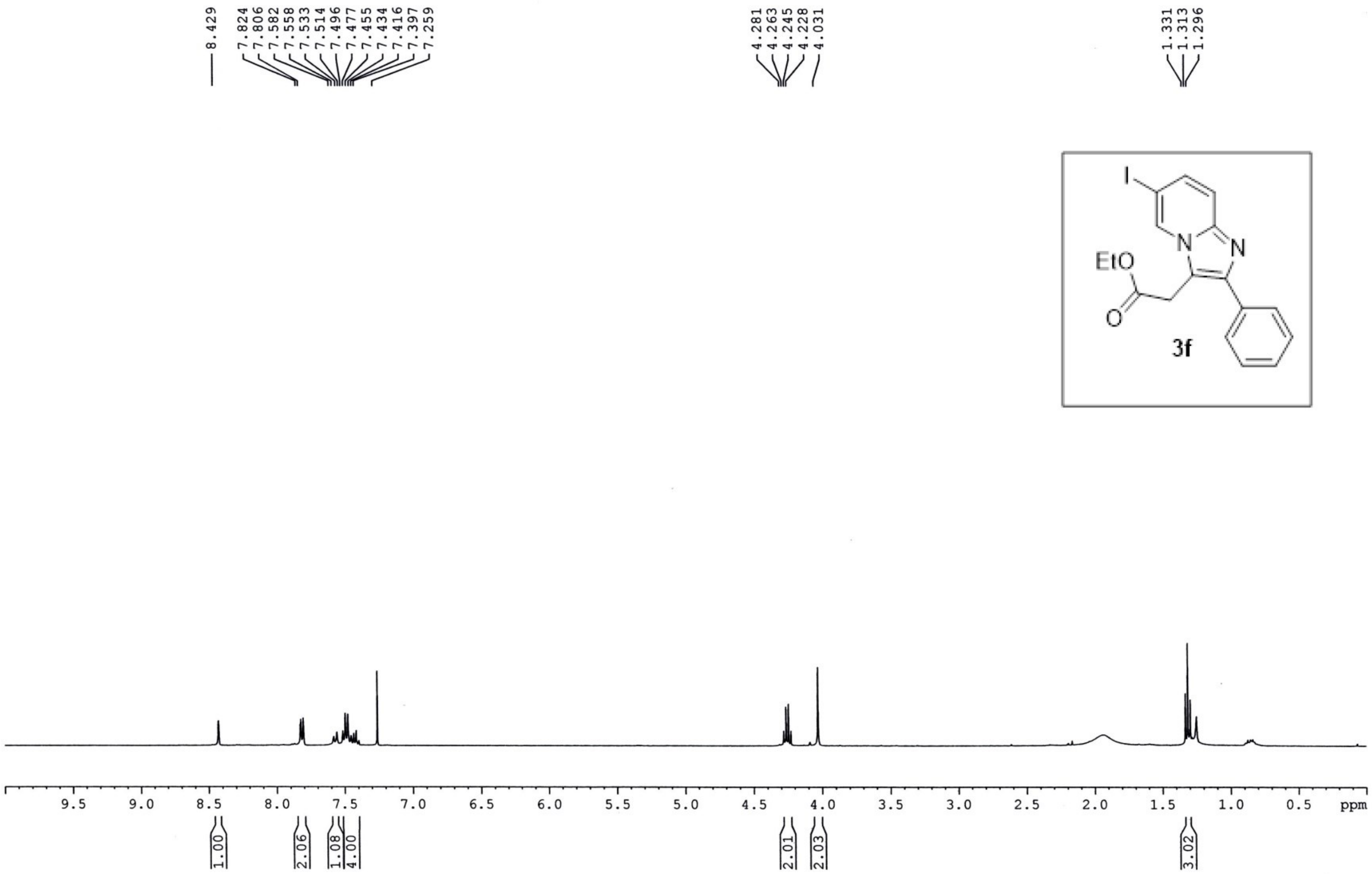
8.429
7.824 7.806 7.582 7.558 7.533 7.514 7.496 7.477 7.455 7.434 7.416 7.397 7.259
4.281 4.263 4.245 4.228 4.031
1.331 1.313 1.296
EtO
O
N
N
I
3f
9.5 9.0 8.5 8.0 7.5 7.0 6.5 6.0 5.5 5.0 4.5 4.0 3.5 3.0 2.5 2.0 1.5 1.0 0.5 ppm
1.00 2.06 1.08 4.00 2.01 2.03 3.02

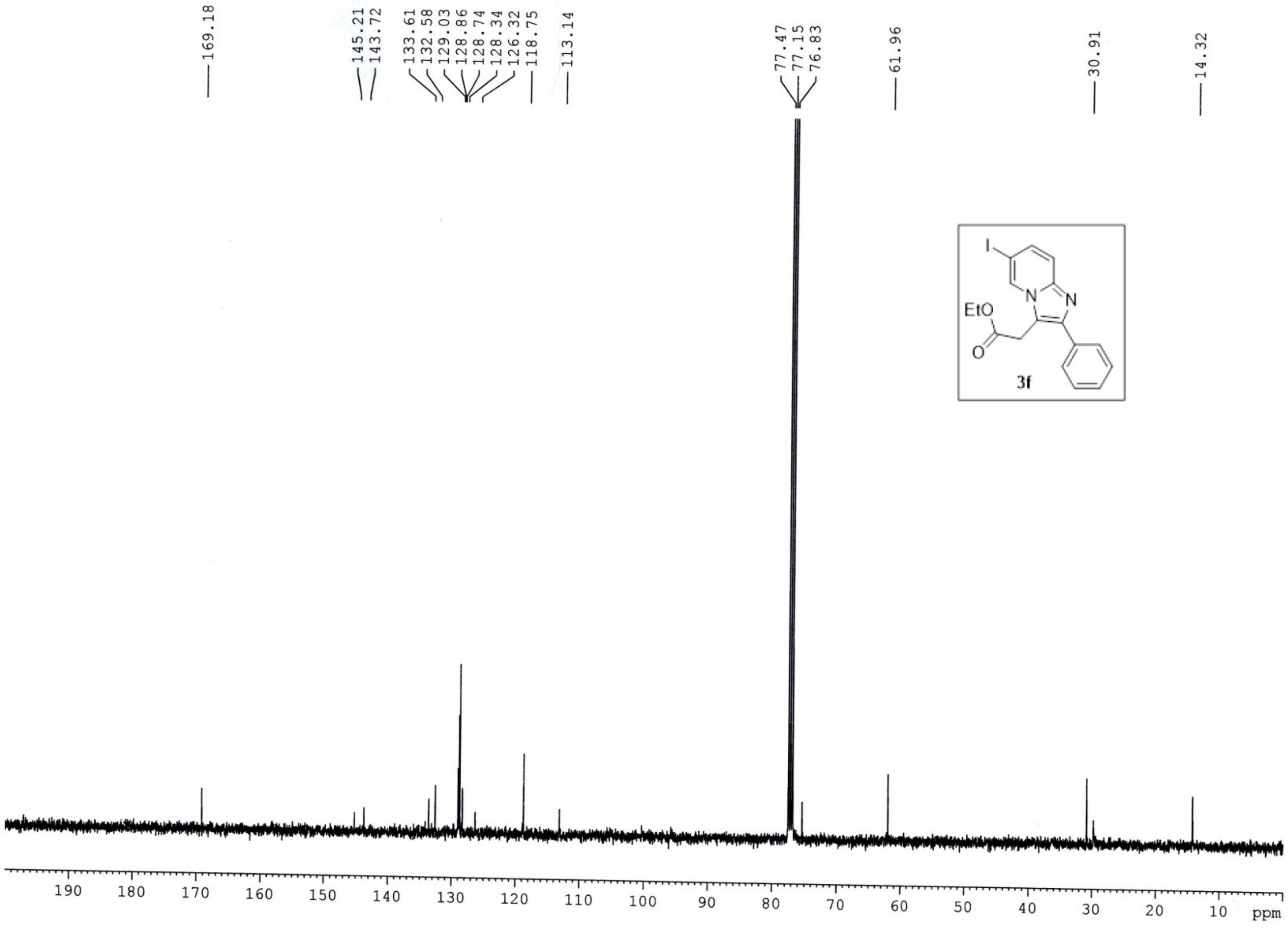
169.18
145.21
143.72
133.61
132.58
129.03
128.86
128.74
128.34
126.32
118.75
113.14
77.47
77.15
76.83
61.96
30.91
14.32
I
N
N
EtO
O
3f
190 180 170 160 150 140 130 120 110 100 90 80 70 60 50 40 30 20 10 ppm

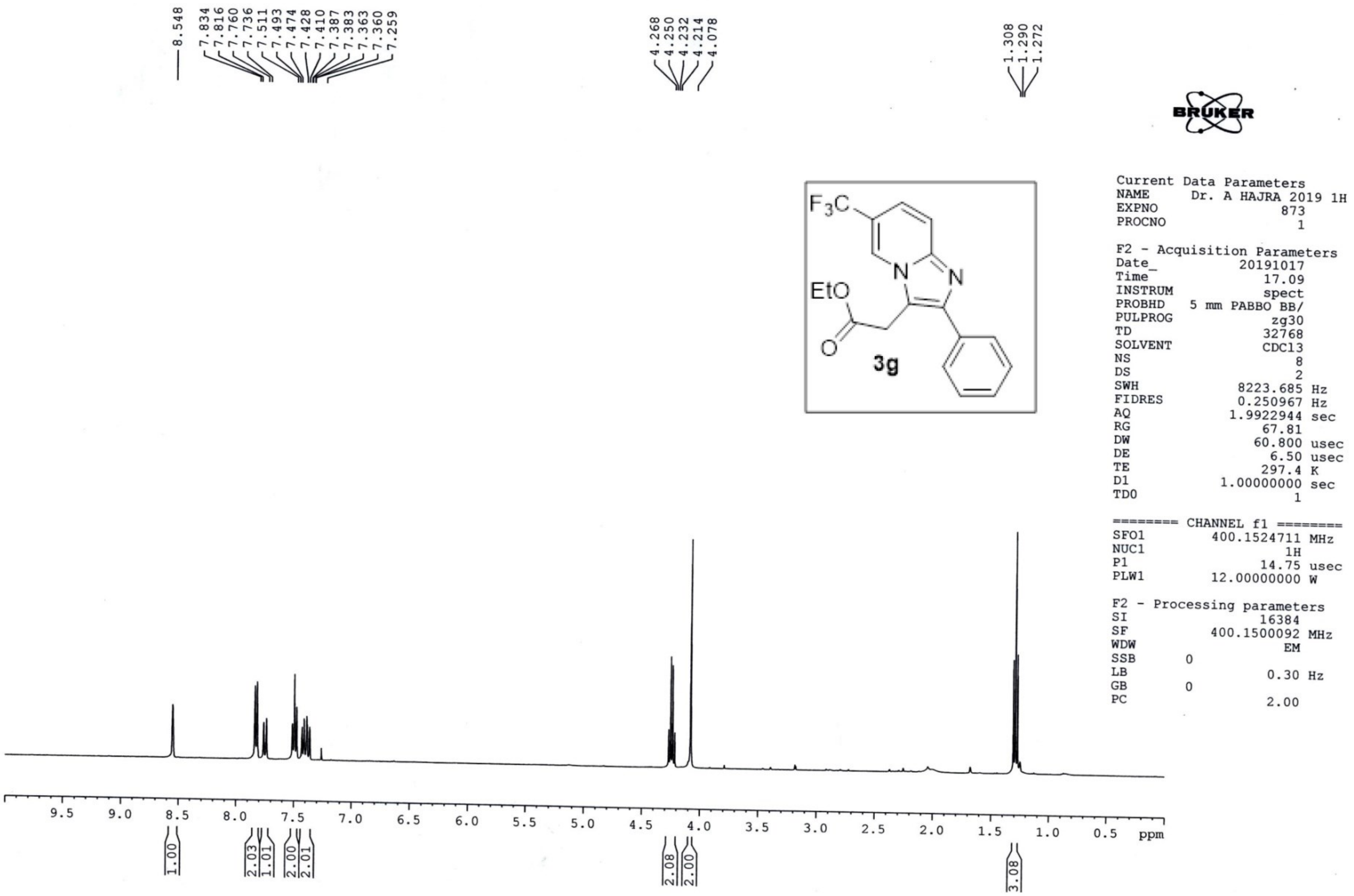
8.548
7.834
7.816
7.760
7.736
7.511
7.493
7.474
7.428
7.410
7.387
7.383
7.363
7.360
7.259
4.268
4.250
4.232
4.214
4.078
1.308
1.290
1.272
F_3C
N
N
EtO
O
3g
BRUKER
Current Data Parameters
NAME Dr. A HAJRA 2019 1H
EXPNO 873
PROCNO 1
F2 - Acquisition Parameters
Date_ 20191017
Time 17.09
INSTRUM spect
PROBHD 5 mm PABBO BB/
PULPROG zg30
TD 32768
SOLVENT CDCl3
NS 8
DS 2
SWH 8223.685 Hz
FIDRES 0.250967 Hz
AQ 1.9922944 sec
RG 67.81
DW 60.800 usec
DE 6.50 usec
TE 297.4 K
D1 1.00000000 sec
TD0 1
======== CHANNEL f1 ========
SFO1 400.1524711 MHz
NUC1 1H
P1 14.75 usec
PLW1 12.00000000 W
F2 - Processing parameters
SI 16384
SF 400.1500092 MHz
WDW EM
SSB 0
LB 0.30 Hz
GB 0
PC 2.00
9.5 9.0 8.5 8.0 7.5 7.0 6.5 6.0 5.5 5.0 4.5 4.0 3.5 3.0 2.5 2.0 1.5 1.0 0.5 ppm
1.00
2.03
1.01
2.00
2.01
2.08
2.00
3.08

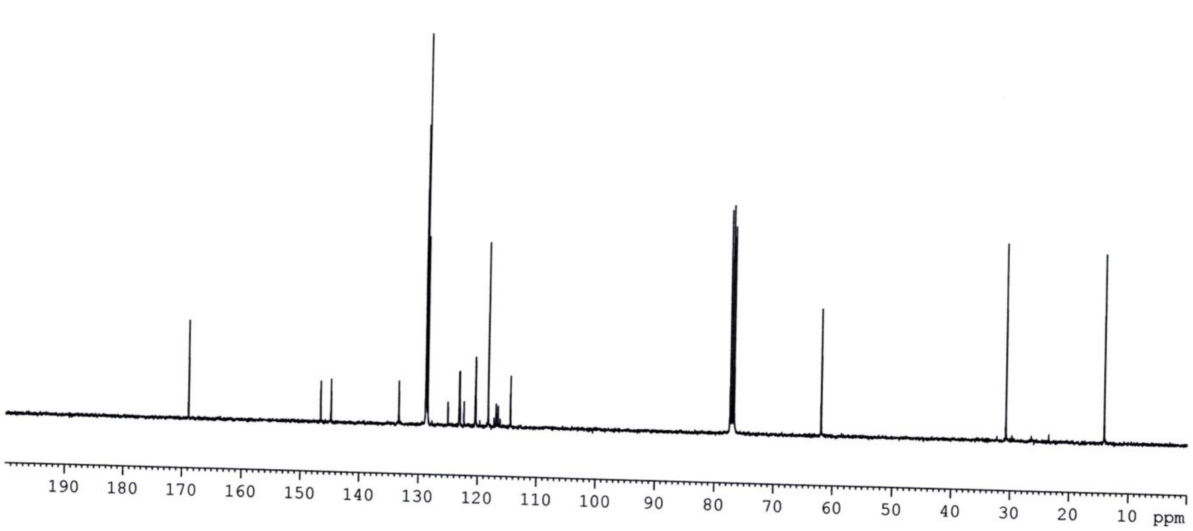

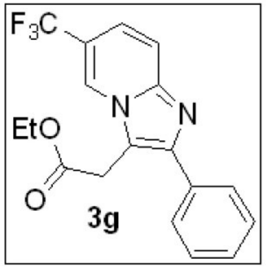

3g

Current Data Parameters
NAME Dr. A HAJRA-2019-13C
EXPNO 416
PROCNO 1

F2 - Acquisition Parameters
Date_ 20191018
Time 9.43
INSTRUM spect
PROBHD 5 mm PABBO BB/
PULPROG zgpg30
TD 32768
SOLVENT CDCl3
NS 960
DS 2
SWH 24038.461 Hz
FIDRES 0.733596 Hz
AQ 0.6815744 sec
RG 186.42
DW 20.800 usec
DE 6.50 usec
TE 302.6 K
D1 2.00000000 sec
D11 0.03000000 sec
TD0 1

======== CHANNEL f1 ========
SFO1 100.6278588 MHz
NUC1 13C
P1 8.90 usec
PLW1 54.00000000 W

======== CHANNEL f2 ========
SFO2 400.1516006 MHz
NUC2 1H
CPDPRG[2 waltz16
PCPD2 90.00 usec
PLW2 12.00000000 W
PLW12 0.32231000 W
PLW13 0.16212000 W

F2 - Processing parameters
SI 16384
SF 100.6177858 MHz
WDW EM
SSB 0
LB 1.00 Hz
GB 0
PC 1.40

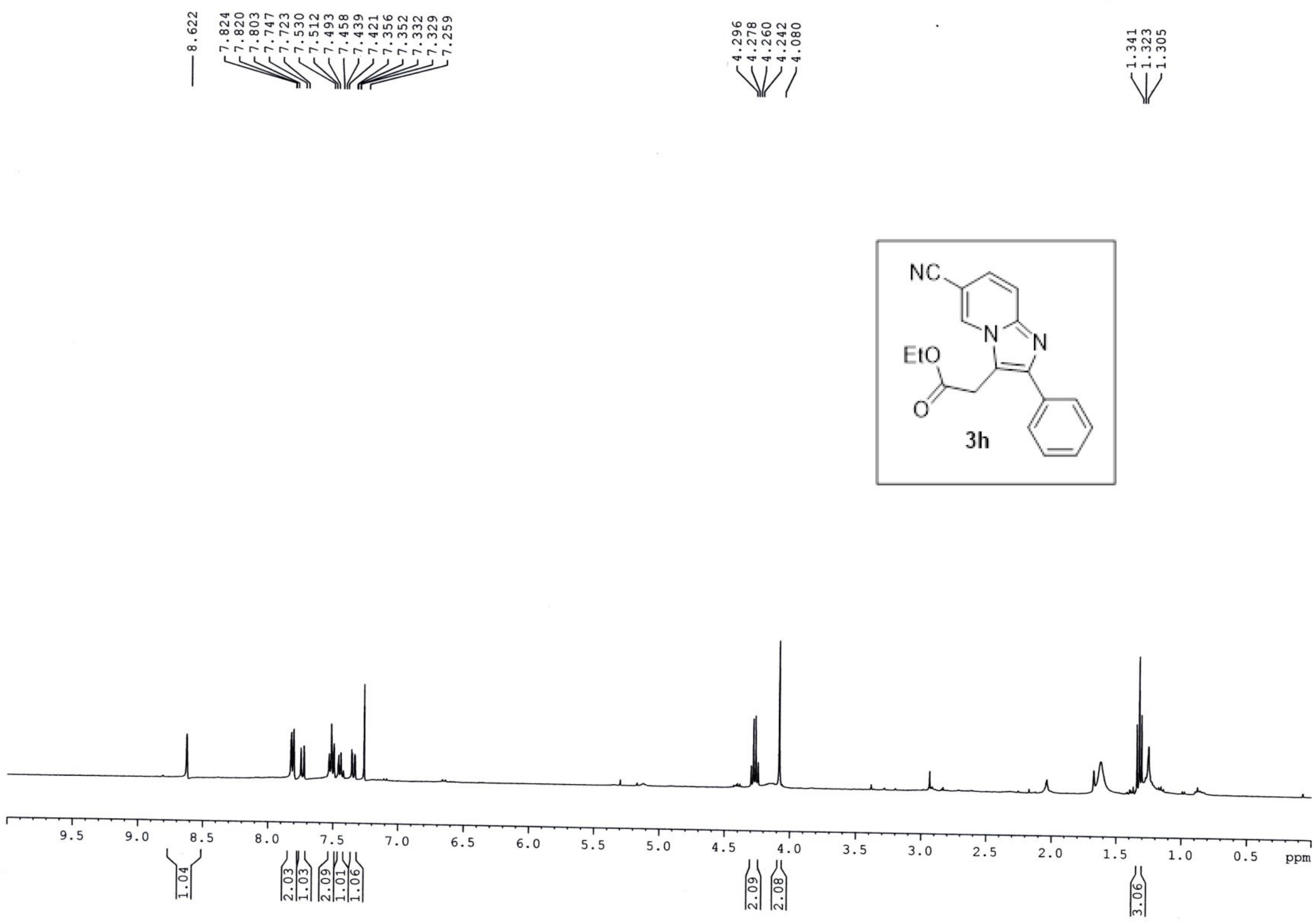
8.622
7.824
7.820
7.803
7.747
7.723
7.530
7.512
7.493
7.458
7.439
7.421
7.356
7.352
7.332
7.329
7.259
4.296
4.278
4.260
4.242
4.080
1.341
1.323
1.305
NC
N
N
EtO
O
3h
9.5 9.0 8.5 8.0 7.5 7.0 6.5 6.0 5.5 5.0 4.5 4.0 3.5 3.0 2.5 2.0 1.5 1.0 0.5 ppm
1.04
2.03
1.03
2.09
1.01
1.06
2.09
2.08
3.06

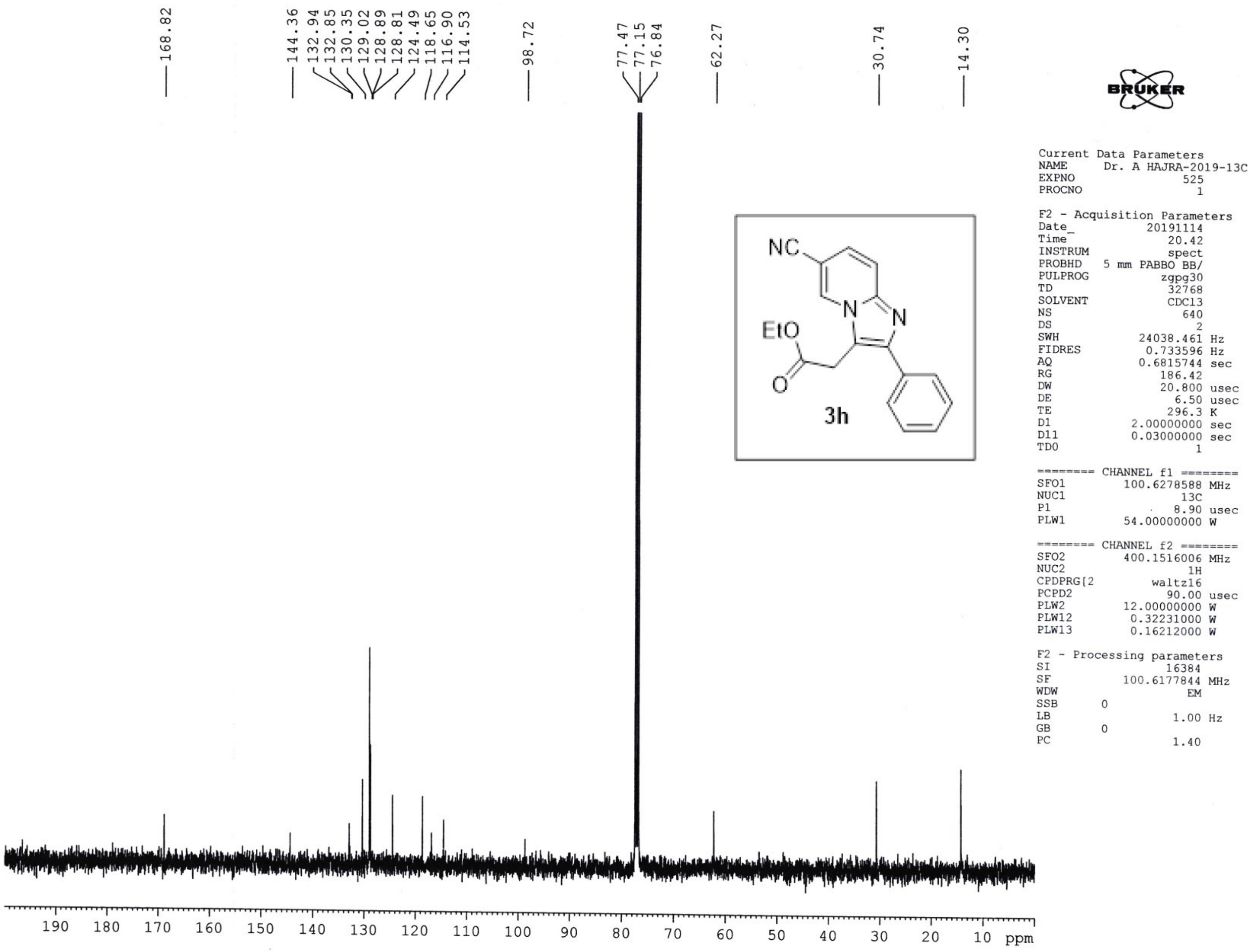
168.82
144.36
132.94
132.85
130.35
129.02
128.89
128.81
124.49
118.65
116.90
114.53
98.72
77.47
77.15
76.84
62.27
30.74
14.30
BRUKER
NC
N
N
EtO
O
3h
Current Data Parameters
NAME Dr. A HAJRA-2019-13C
EXPNO 525
PROCNO 1
F2 - Acquisition Parameters
Date_ 20191114
Time 20.42
INSTRUM spect
PROBHD 5 mm PABBO BB/
PULPROG zgpg30
TD 32768
SOLVENT CDCl3
NS 640
DS 2
SWH 24038.461 Hz
FIDRES 0.733596 Hz
AQ 0.6815744 sec
RG 186.42
DW 20.800 usec
DE 6.50 usec
TE 296.3 K
D1 2.00000000 sec
D11 0.03000000 sec
TD0 1
======== CHANNEL f1 ========
SFO1 100.6278588 MHz
NUC1 13C
P1 8.90 usec
PLW1 54.00000000 W
======== CHANNEL f2 ========
SFO2 400.1516006 MHz
NUC2 1H
CPDPRG[2 waltz16
PCPD2 90.00 usec
PLW2 12.00000000 W
PLW12 0.32231000 W
PLW13 0.16212000 W
F2 - Processing parameters
SI 16384
SF 100.6177844 MHz
WDW EM
SSB 0
LB 1.00 Hz
GB 0
PC 1.40
190 180 170 160 150 140 130 120 110 100 90 80 70 60 50 40 30 20 10 ppm

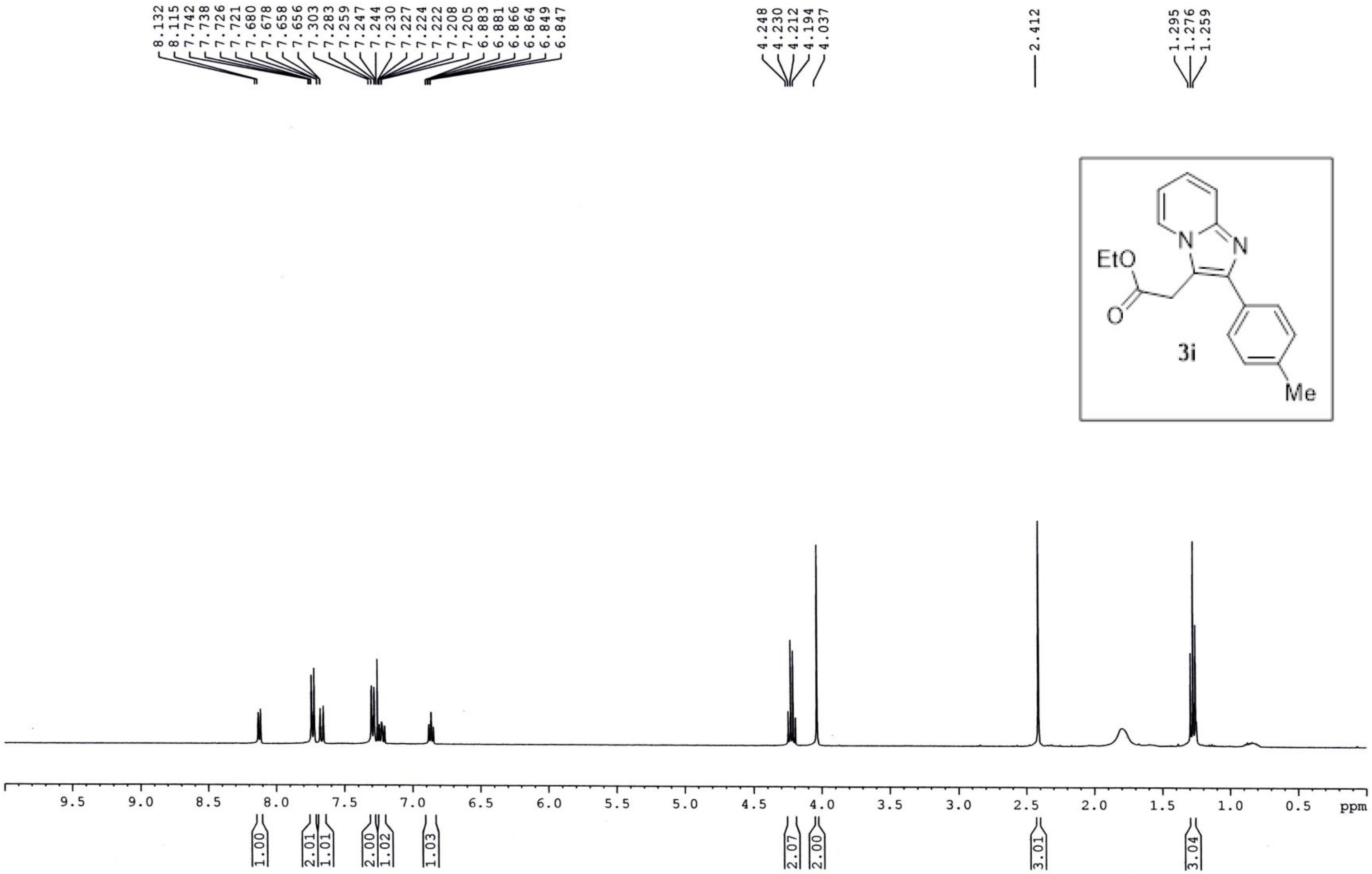

8.132
8.115
7.742
7.738
7.726
7.721
7.680
7.678
7.658
7.656
7.303
7.283
7.259
7.247
7.244
7.230
7.227
7.224
7.222
7.208
7.205
6.883
6.881
6.866
6.864
6.849
6.847
4.248
4.230
4.212
4.194
4.037
2.412
1.295
1.276
1.259
EtO
O
N
N
3i
Me
9.5 9.0 8.5 8.0 7.5 7.0 6.5 6.0 5.5 5.0 4.5 4.0 3.5 3.0 2.5 2.0 1.5 1.0 0.5 ppm
1.00
2.01
1.01
2.00
1.02
1.03
2.07
2.00
3.01
3.04

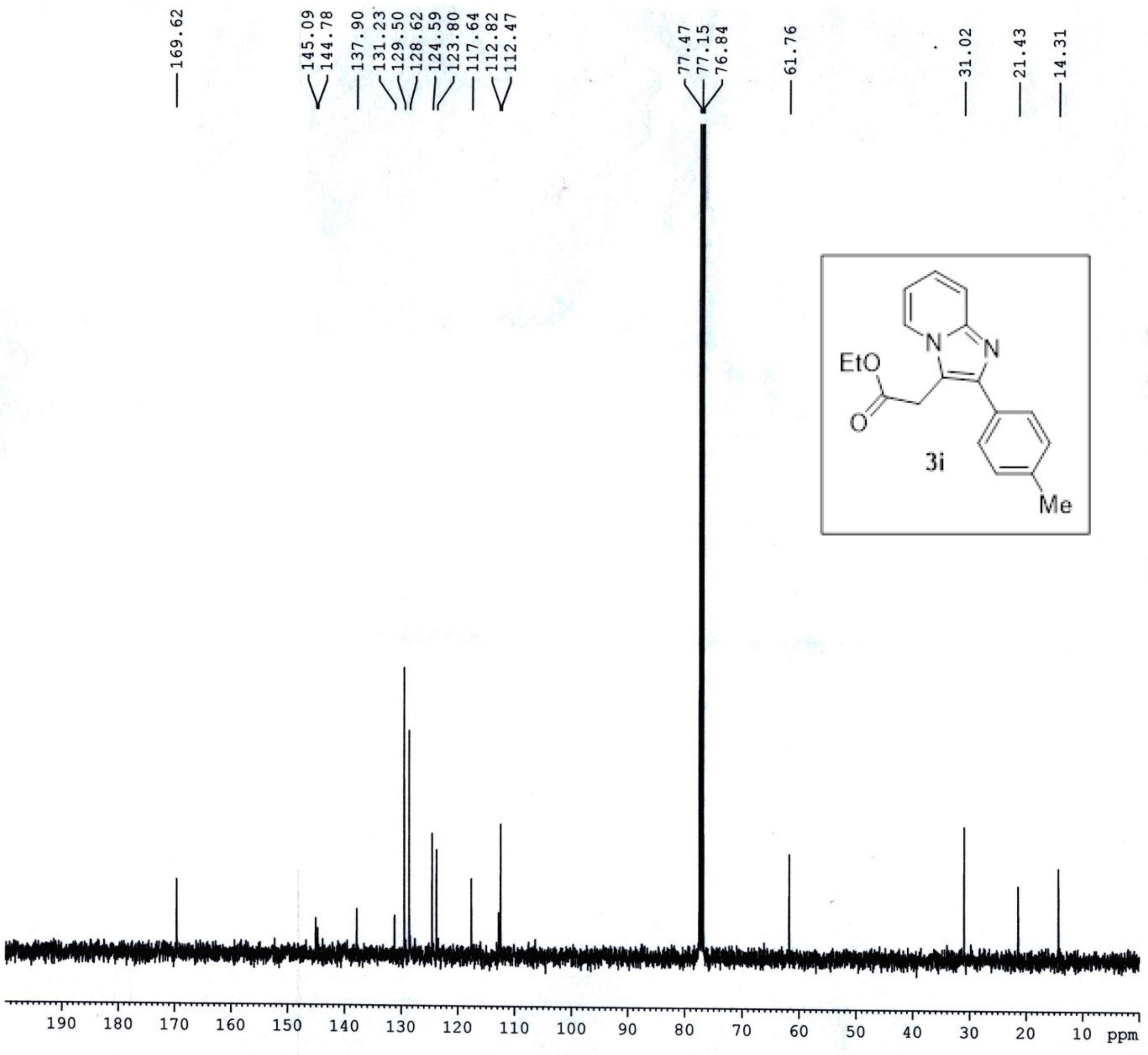

BRUKER

Current Data Parameters
NAME Dr. A HAJRA-2019-13C
EXPNO 342
PROCNO 1

F2 - Acquisition Parameters
Date_ 20190826
Time 13.02
INSTRUM spect
PROBHD 5 mm PABBO BB/
PULPROG zgpg30
TD 32768
SOLVENT CDCl3
NS 410
DS 2
SWH 24038.461 Hz
FIDRES 0.733596 Hz
AQ 0.6815744 sec
RG 148.91
DW 20.800 usec
DE 6.50 usec
TE 298.7 K
D1 2.00000000 sec
D11 0.03000000 sec
TD0 1

======== CHANNEL f1 ========
SFO1 100.6278588 MHz
NUC1 13C
P1 8.90 usec
PLW1 54.00000000 W

======== CHANNEL f2 ========
SFO2 400.1516006 MHz
NUC2 1H
CPDPRG[2 waltz16
PCPD2 90.00 usec
PLW2 12.00000000 W
PLW12 0.32231000 W
PLW13 0.16212000 W

F2 - Processing parameters
SI 16384
SF 100.6177841 MHz
WDW EM
SSB 0
LB 1.00 Hz
GB 0
PC 1.40

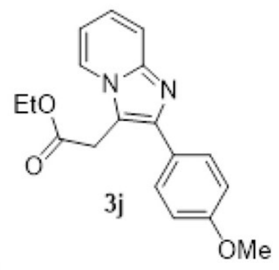
EtO
O
N
N
3j
OMe

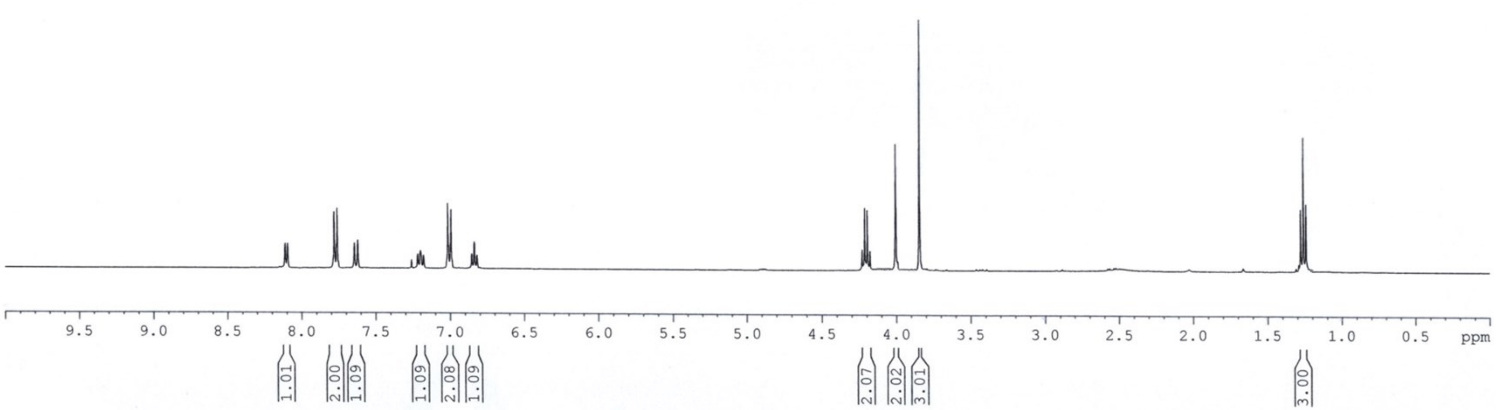
8.110
8.093
7.779
7.757
7.642
7.620
7.219
7.217
7.202
7.199
7.197
7.194
7.180
7.015
6.993
6.855
6.854
6.838
6.836
6.822
6.820
4.230
4.213
4.195
4.177
4.003
3.842
1.279
1.261
1.244
9.5
9.0
8.5
8.0
7.5
7.0
6.5
6.0
5.5
5.0
4.5
4.0
3.5
3.0
2.5
2.0
1.5
1.0
0.5
ppm
1.01
2.00
1.09
1.09
2.08
1.09
2.07
2.02
3.01
3.00

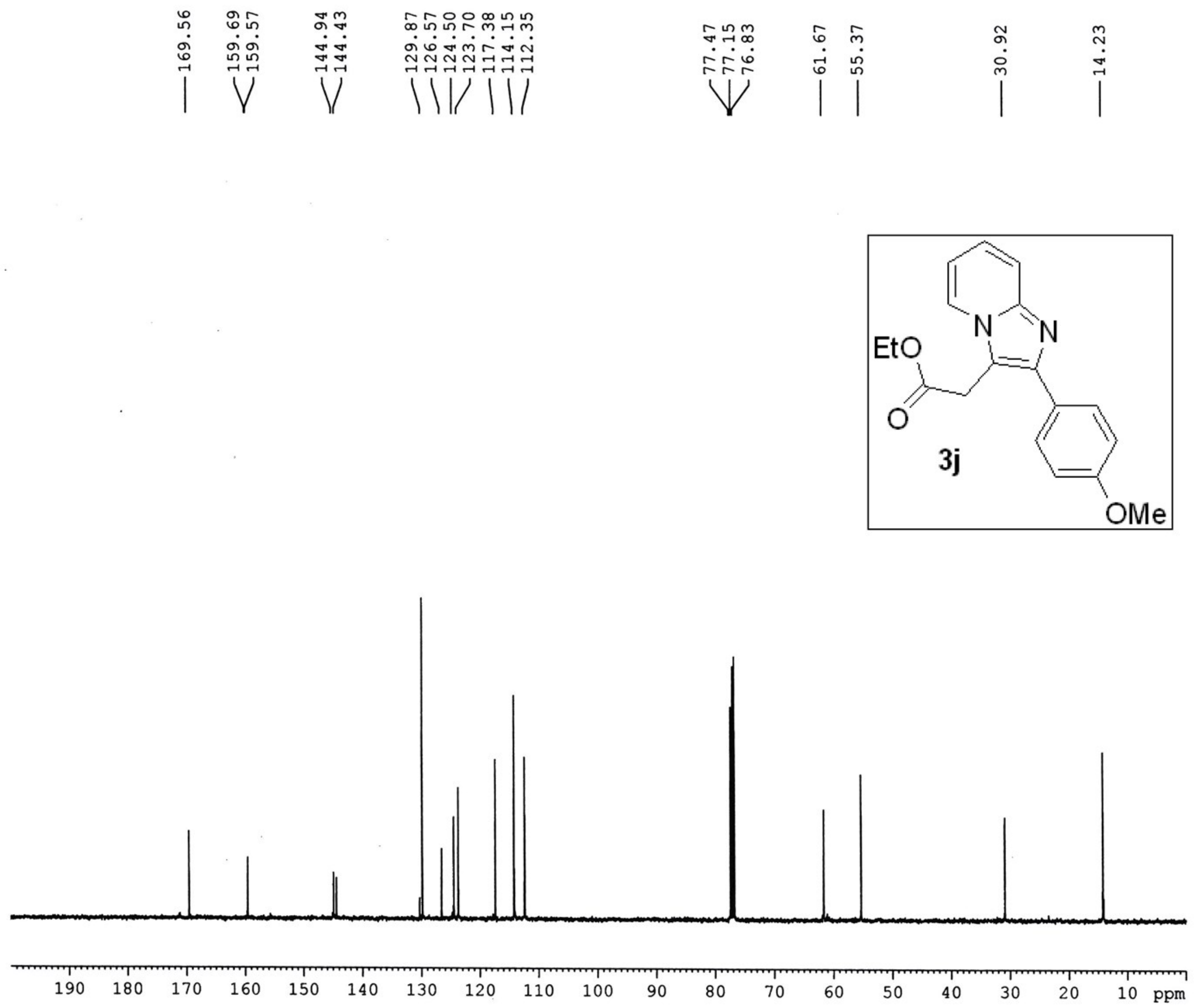
169.56
159.69
159.57
144.94
144.43
129.87
126.57
124.50
123.70
117.38
114.15
112.35
77.47
77.15
76.83
61.67
55.37
30.92
14.23
EtO
O
N
N
3j
OMe
190 180 170 160 150 140 130 120 110 100 90 80 70 60 50 40 30 20 10 ppm

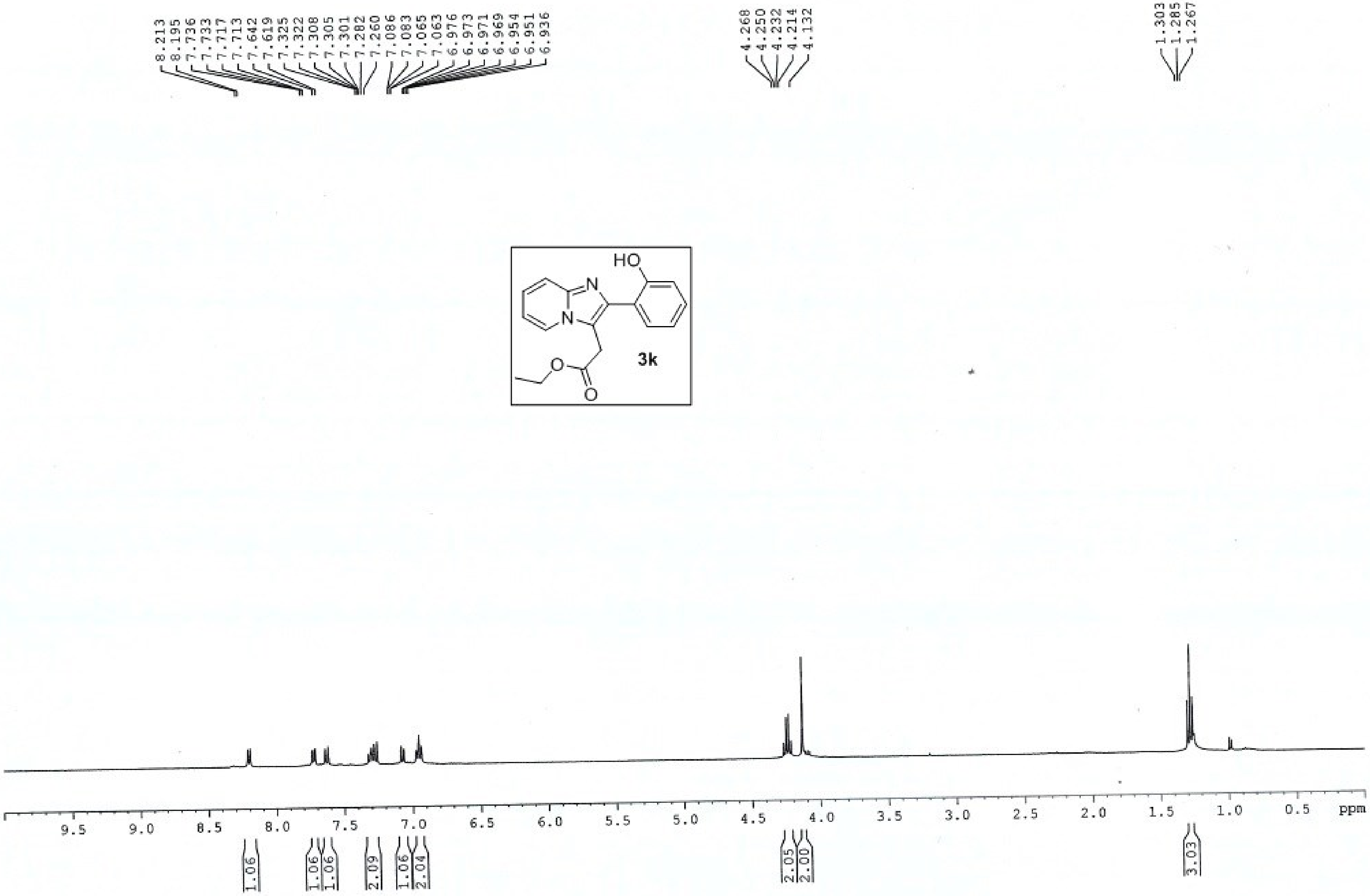
8.213
8.195
7.736
7.733
7.717
7.713
7.642
7.619
7.325
7.322
7.308
7.305
7.301
7.282
7.260
7.086
7.083
7.065
7.063
6.976
6.973
6.971
6.969
6.954
6.951
6.936
4.268
4.250
4.232
4.214
4.132
1.303
1.285
1.267
HO
N
N
O
O
3k
9.5 9.0 8.5 8.0 7.5 7.0 6.5 6.0 5.5 5.0 4.5 4.0 3.5 3.0 2.5 2.0 1.5 1.0 0.5 ppm
1.06
1.06
1.06
2.09
1.06
2.04
2.05
2.00
3.03

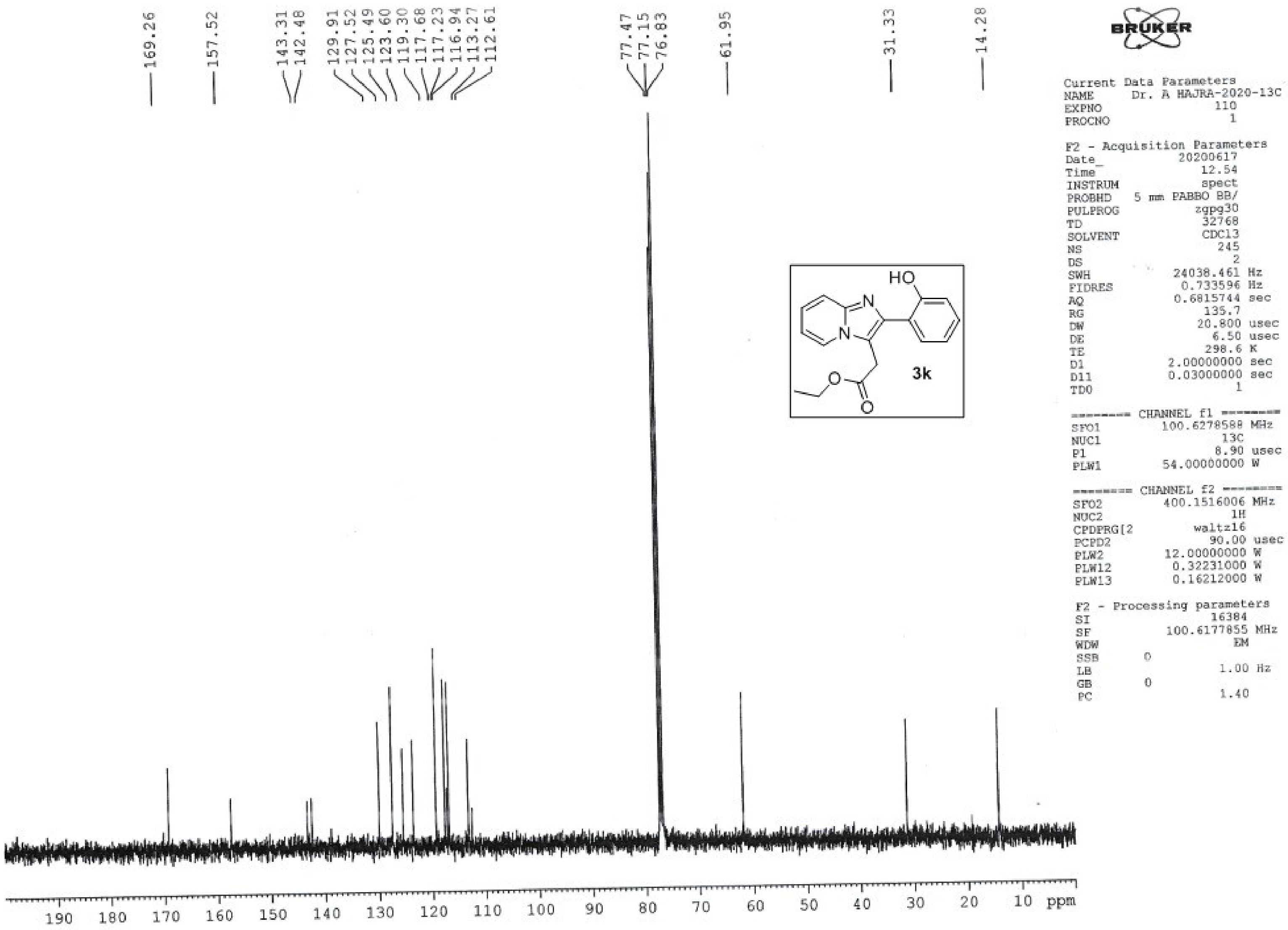
169.26
157.52
143.31
142.48
129.91
127.52
125.49
123.60
119.30
117.68
117.23
116.94
113.27
112.61
77.47
77.15
76.83
61.95
31.33
14.28
HO
N
N
O
O
3k
BRUKER
Current Data Parameters
NAME Dr. A HAJRA-2020-13C
EXPNO 110
PROCNO 1
F2 - Acquisition Parameters
Date_ 20200617
Time 12.54
INSTRUM spect
PROBHD 5 mm PABBO BB/
PULPROG zgpg30
TD 32768
SOLVENT CDCl3
NS 245
DS 2
SWH 24038.461 Hz
FIDRES 0.733596 Hz
AQ 0.6815744 sec
RG 135.7
DW 20.800 usec
DE 6.50 usec
TE 298.6 K
D1 2.00000000 sec
D11 0.03000000 sec
TD0 1
======== CHANNEL f1 ========
SFO1 100.6278588 MHz
NUC1 13C
P1 8.90 usec
PLW1 54.00000000 W
======== CHANNEL f2 ========
SFO2 400.1516006 MHz
NUC2 1H
CPDPRG[2 waltz16
PCPD2 90.00 usec
PLW2 12.00000000 W
PLW12 0.32231000 W
PLW13 0.16212000 W
F2 - Processing parameters
SI 16384
SF 100.6177855 MHz
WDW EM
SSB 0
LB 1.00 Hz
GB 0
PC 1.40
190 180 170 160 150 140 130 120 110 100 90 80 70 60 50 40 30 20 10 ppm

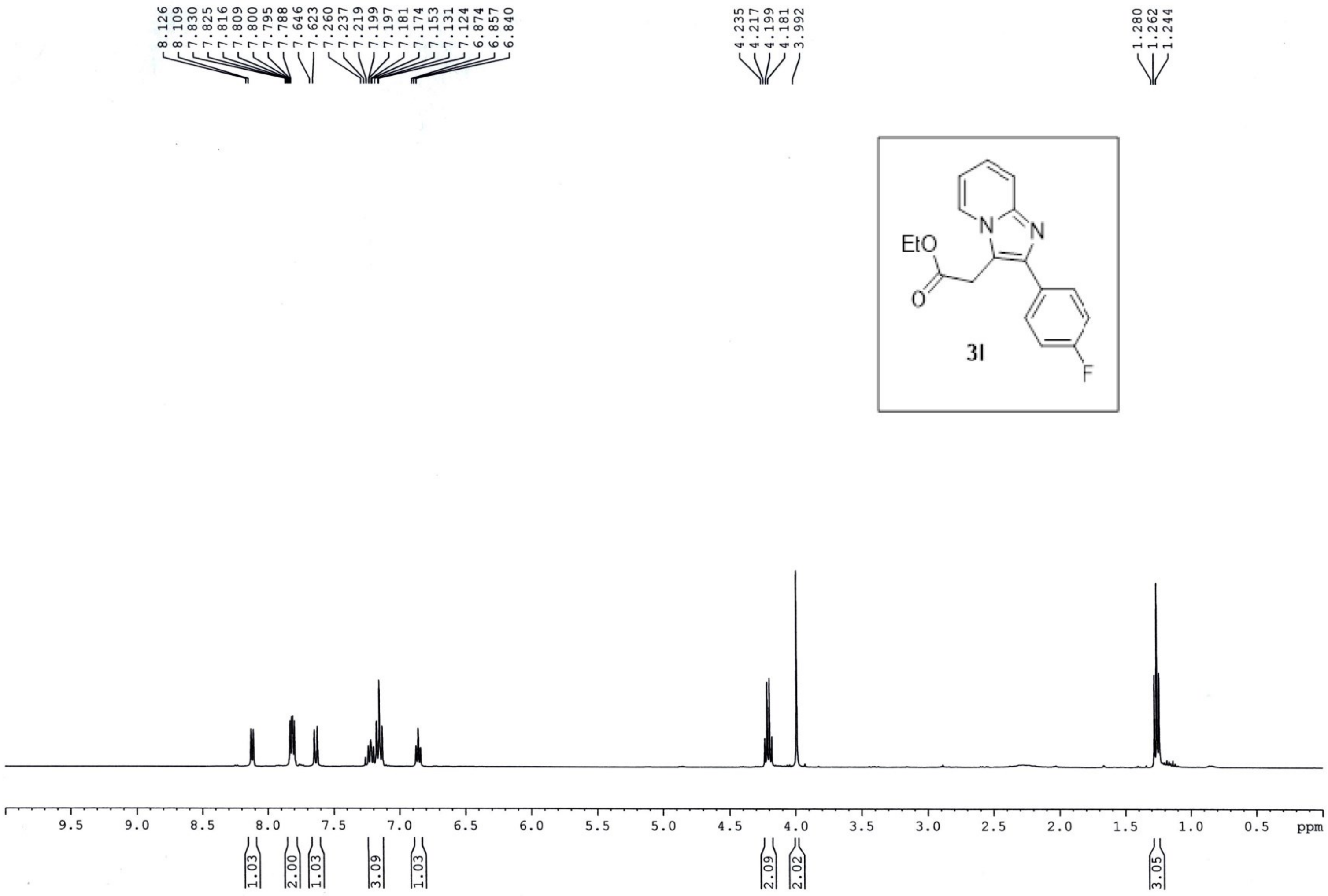
8.126 8.109 7.830 7.825 7.816 7.809 7.800 7.795 7.788 7.646 7.623 7.260 7.237 7.219 7.199 7.197 7.181 7.174 7.153 7.131 7.124 6.874 6.857 6.840
4.235 4.217 4.199 4.181 3.992
1.280 1.262 1.244
EtO
N
N
O
F
3l
9.5 9.0 8.5 8.0 7.5 7.0 6.5 6.0 5.5 5.0 4.5 4.0 3.5 3.0 2.5 2.0 1.5 1.0 0.5 ppm
1.03 2.00 1.03 3.09 1.03 2.09 2.02 3.05

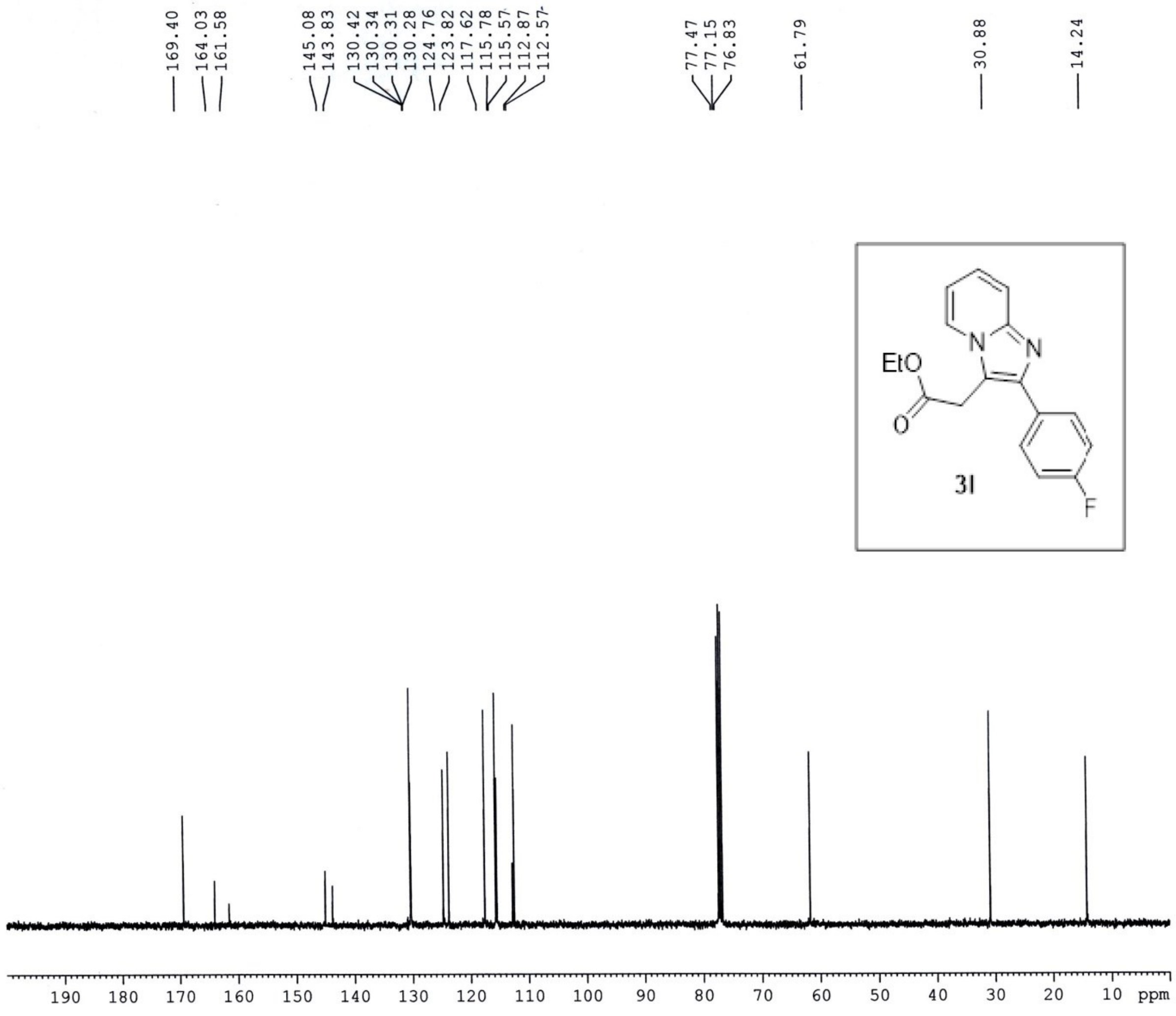

Current Data Parameters
NAME Dr. A HAJRA-2019-13C
EXPNO 376
PROCNO 1

F2 - Acquisition Parameters
Date_ 20190921
Time 20.17
INSTRUM spect
PROBHD 5 mm PABBO BB/
PULPROG zgpg30
TD 32768
SOLVENT CDCl3
NS 200
DS 2
SWH 24038.461 Hz
FIDRES 0.733596 Hz
AQ 0.6815744 sec
RG 62.69
DW 20.800 usec
DE 6.50 usec
TE 299.1 K
D1 2.00000000 sec
D11 0.03000000 sec
TD0 1

======== CHANNEL f1 ========
SFO1 100.6278588 MHz
NUC1 13C
P1 8.90 usec
PLW1 54.00000000 W

======== CHANNEL f2 ========
SFO2 400.1516006 MHz
NUC2 1H
CPDPRG[2 waltz16
PCPD2 90.00 usec
PLW2 12.00000000 W
PLW12 0.32231000 W
PLW13 0.16212000 W

F2 - Processing parameters
SI 16384
SF 100.6177887 MHz
WDW EM
SSB 0
LB 1.00 Hz
GB 0
PC 1.40

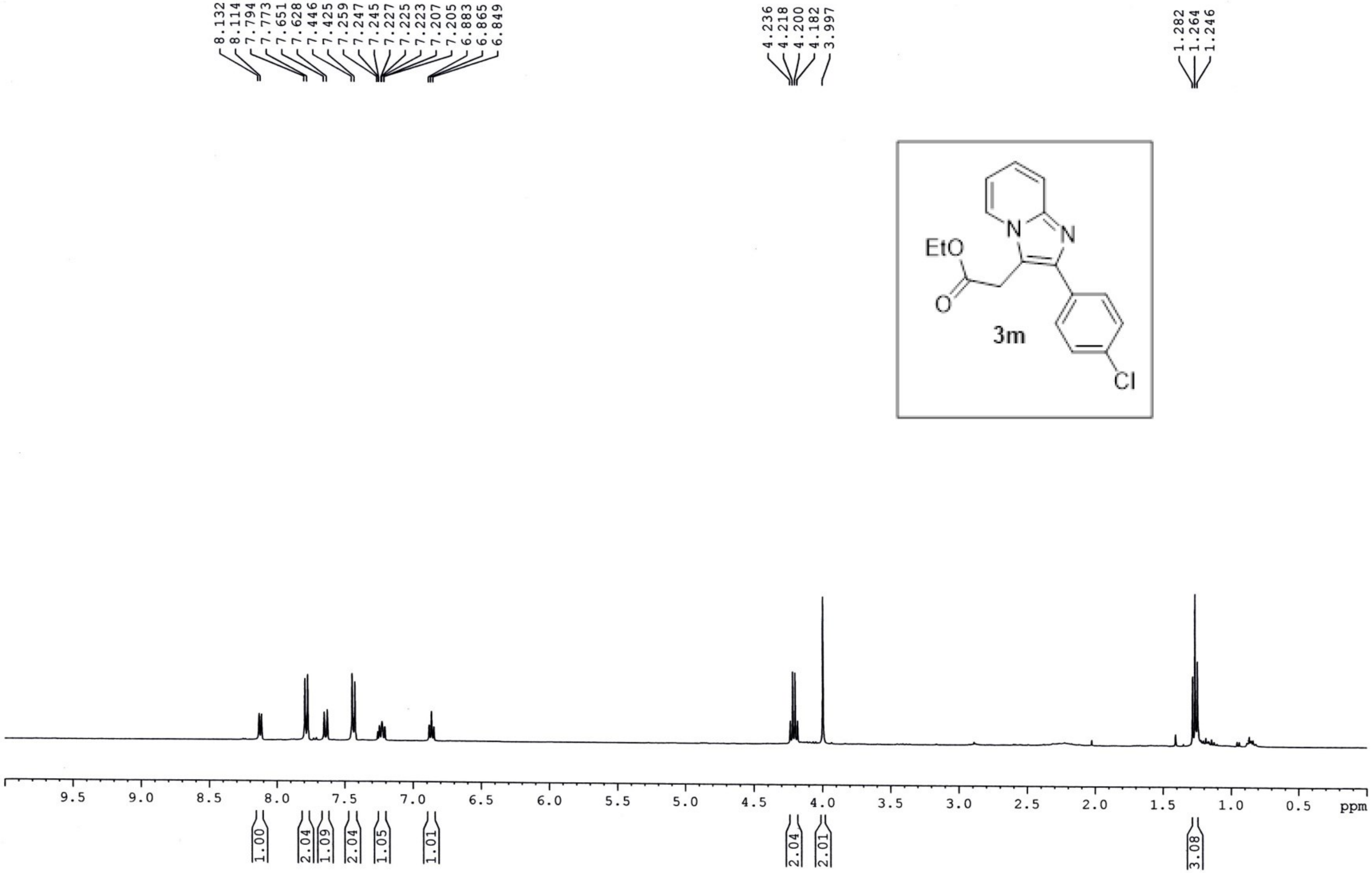
8.132
8.114
7.794
7.773
7.651
7.628
7.446
7.425
7.259
7.247
7.245
7.227
7.225
7.223
7.207
7.205
6.883
6.865
6.849
4.236
4.218
4.200
4.182
3.997
1.282
1.264
1.246
EtO
N
O
3m
Cl
9.5
9.0
8.5
8.0
7.5
7.0
6.5
6.0
5.5
5.0
4.5
4.0
3.5
3.0
2.5
2.0
1.5
1.0
0.5
ppm
1.00
2.04
1.09
2.04
1.05
1.01
2.04
2.01
3.08

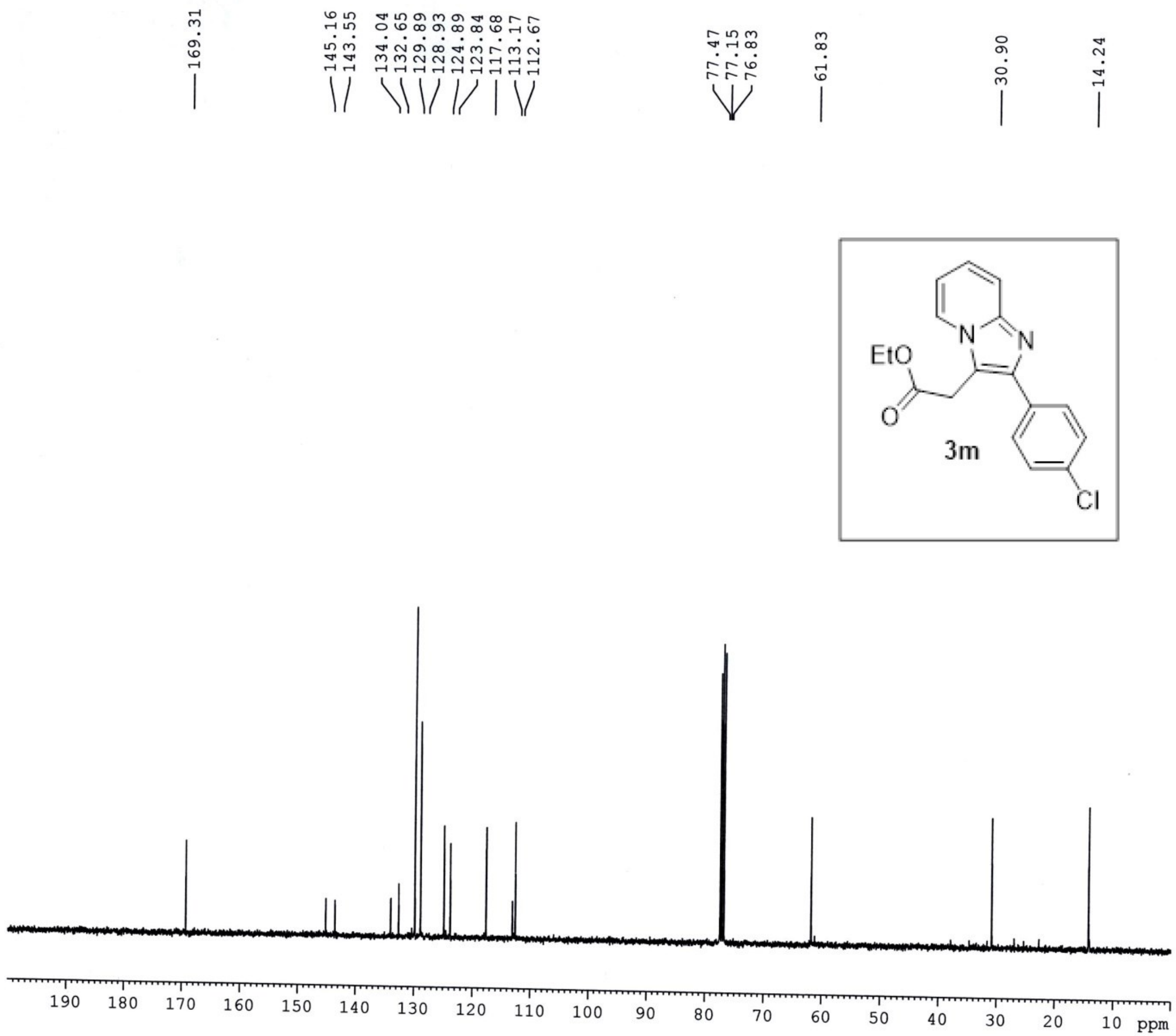

Current Data Parameters
NAME Dr. A HAJRA-2019-13C
EXPNO 384
PROCNO 1

F2 - Acquisition Parameters
Date_ 20190924
Time 11.29
INSTRUM spect
PROBHD 5 mm PABBO BB/
PULPROG zgpg30
TD 32768
SOLVENT CDCl3
NS 200
DS 2
SWH 24038.461 Hz
FIDRES 0.733596 Hz
AQ 0.6815744 sec
RG 62.69
DW 20.800 usec
DE 6.50 usec
TE 298.3 K
D1 2.00000000 sec
D11 0.03000000 sec
TD0 1

======== CHANNEL f1 ========
SFO1 100.6278588 MHz
NUC1 13C
P1 8.90 usec
PLW1 54.00000000 W

======== CHANNEL f2 ========
SFO2 400.1516006 MHz
NUC2 1H
CPDPRG[2 waltz16
PCPD2 90.00 usec
PLW2 12.00000000 W
PLW12 0.32231000 W
PLW13 0.16212000 W

F2 - Processing parameters
SI 16384
SF 100.6177888 MHz
WDW EM
SSB 0
LB 1.00 Hz
GB 0
PC 1.40

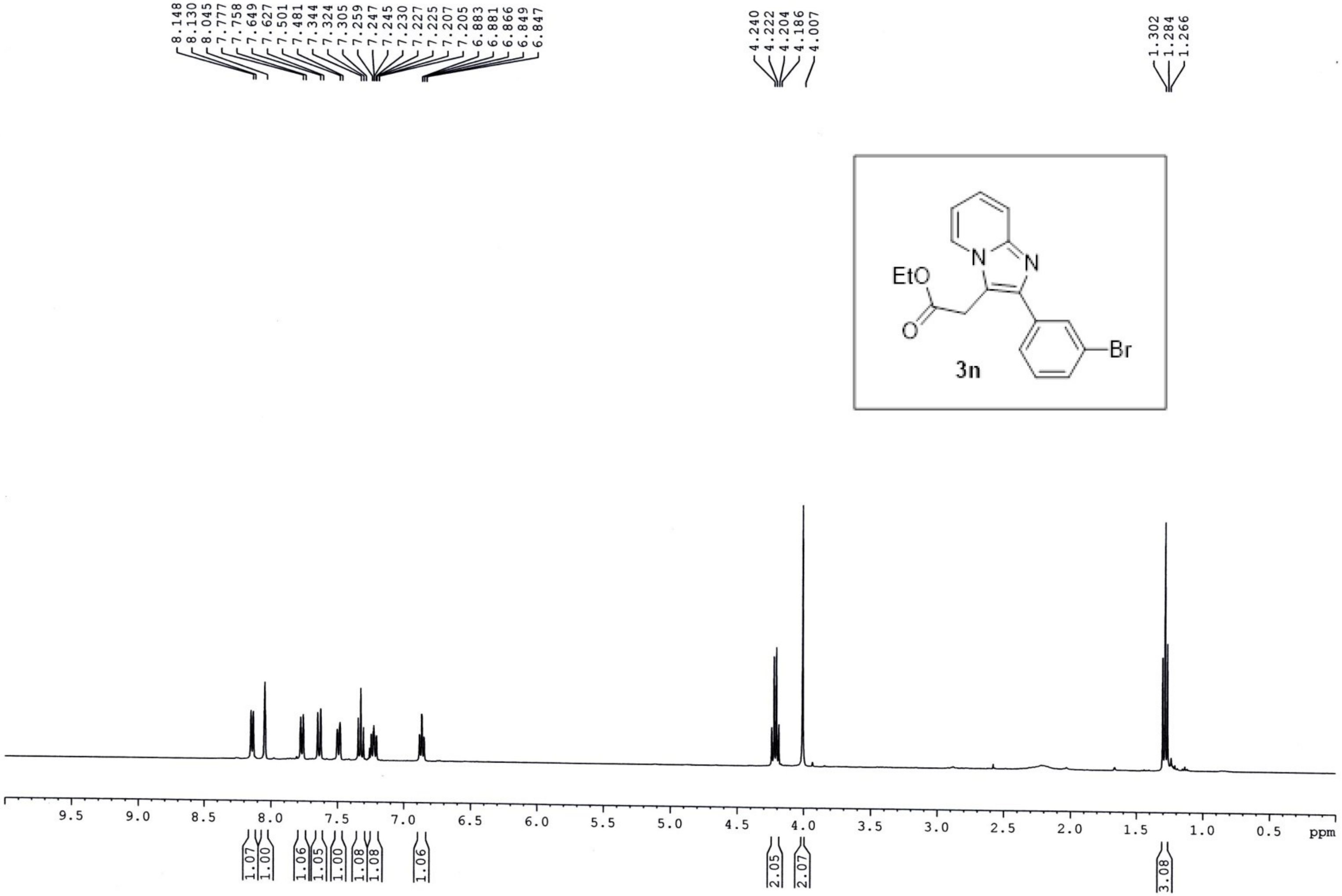
EtO
N
N
O
Br
3n

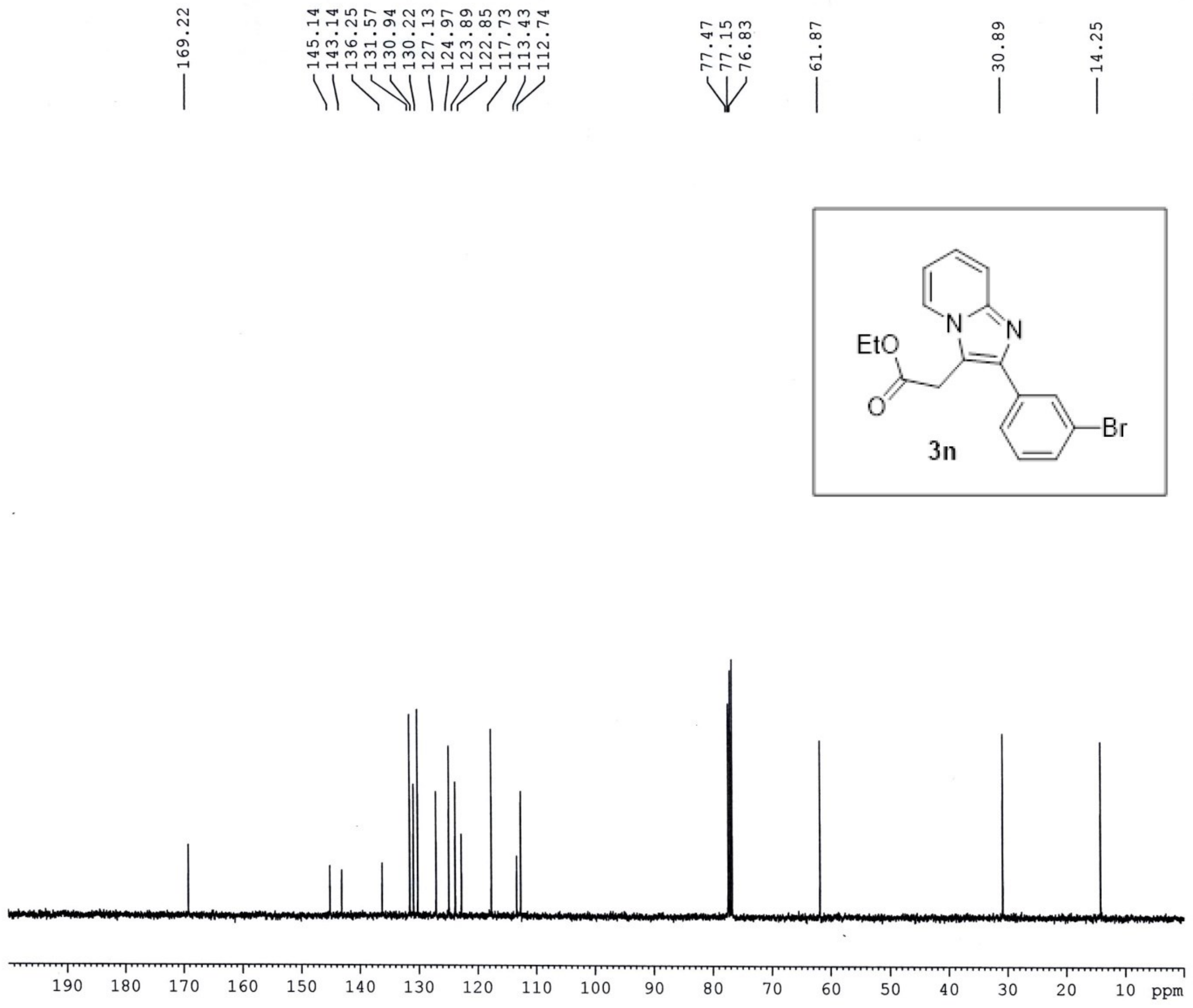

3n

```
Current Data Parameters
NAME       Dr. A HAJRA-2019-13C
EXPNO                   397
PROCNO                    1

F2 - Acquisition Parameters
Date_              20191002
Time                  10.54
INSTRUM               spect
PROBHD   5 mm PABBO BB/
PULPROG              zgpg30
TD                    32768
SOLVENT               CDCl3
NS                      120
DS                        2
SWH               24038.461 Hz
FIDRES             0.733596 Hz
AQ                0.6815744 sec
RG                   186.42
DW                   20.800 usec
DE                     6.50 usec
TE                    298.9 K
D1               2.00000000 sec
D11              0.03000000 sec
TD0                       1

======== CHANNEL f1 ========
SFO1            100.6278588 MHz
NUC1                    13C
P1                     8.90 usec
PLW1            54.00000000 W

======== CHANNEL f2 ========
SFO2            400.1516006 MHz
NUC2                     1H
CPDPRG[2             waltz16
PCPD2                 90.00 usec
PLW2            12.00000000 W
PLW12            0.32231000 W
PLW13            0.16212000 W

F2 - Processing parameters
SI                    16384
SF              100.6177902 MHz
WDW                      EM
SSB      0
LB                     1.00 Hz
GB       0
PC                     1.40
```

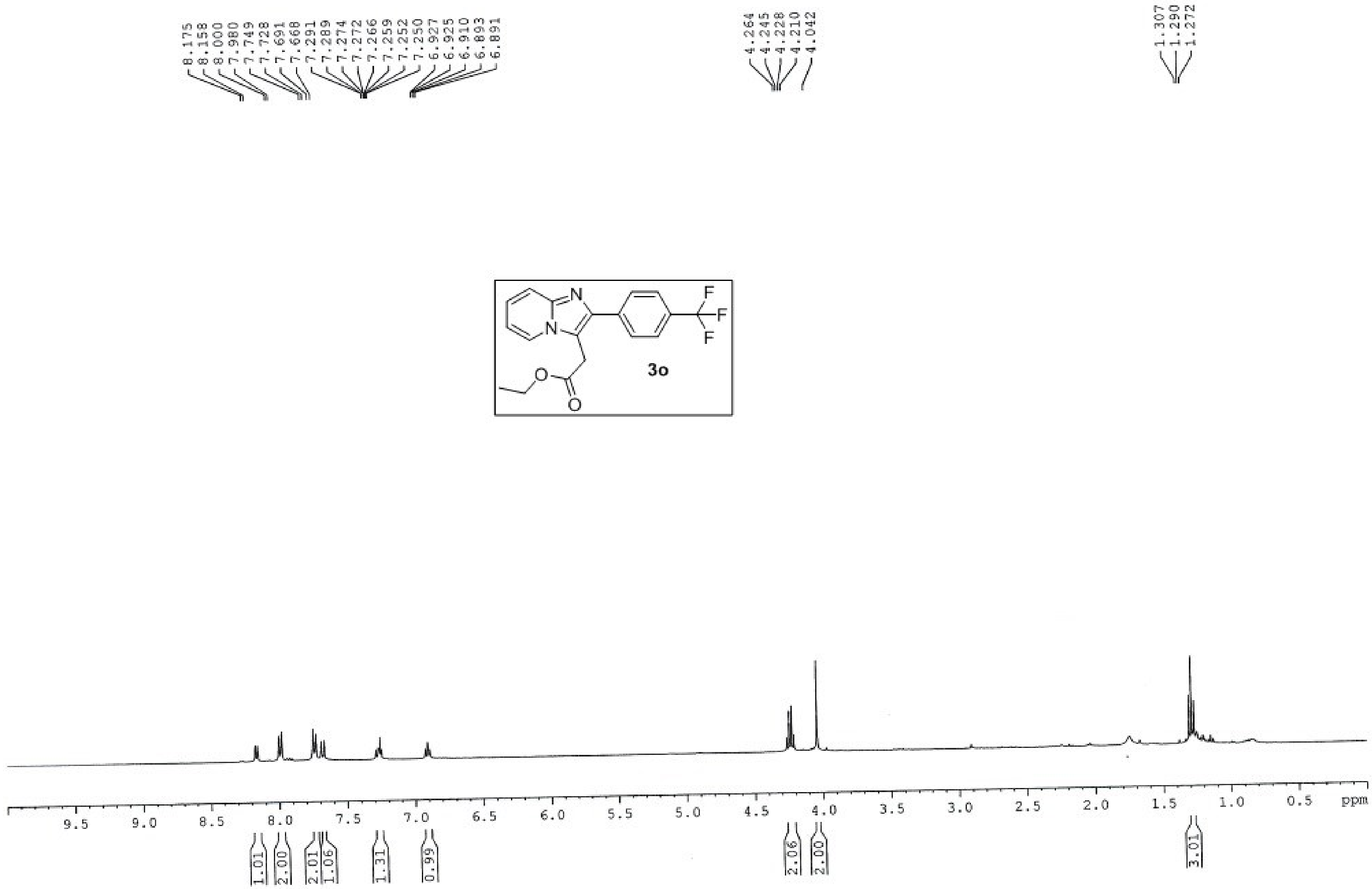
8.175
8.158
8.000
7.980
7.749
7.728
7.691
7.668
7.291
7.289
7.274
7.272
7.266
7.259
7.252
7.250
6.927
6.925
6.910
6.893
6.891
4.264
4.245
4.228
4.210
4.042
1.307
1.290
1.272
F
F
F
N
N
O
O
3o
9.5
9.0
8.5
8.0
7.5
7.0
6.5
6.0
5.5
5.0
4.5
4.0
3.5
3.0
2.5
2.0
1.5
1.0
0.5
ppm
1.01
2.00
2.01
1.06
1.31
0.99
2.06
2.00
3.01

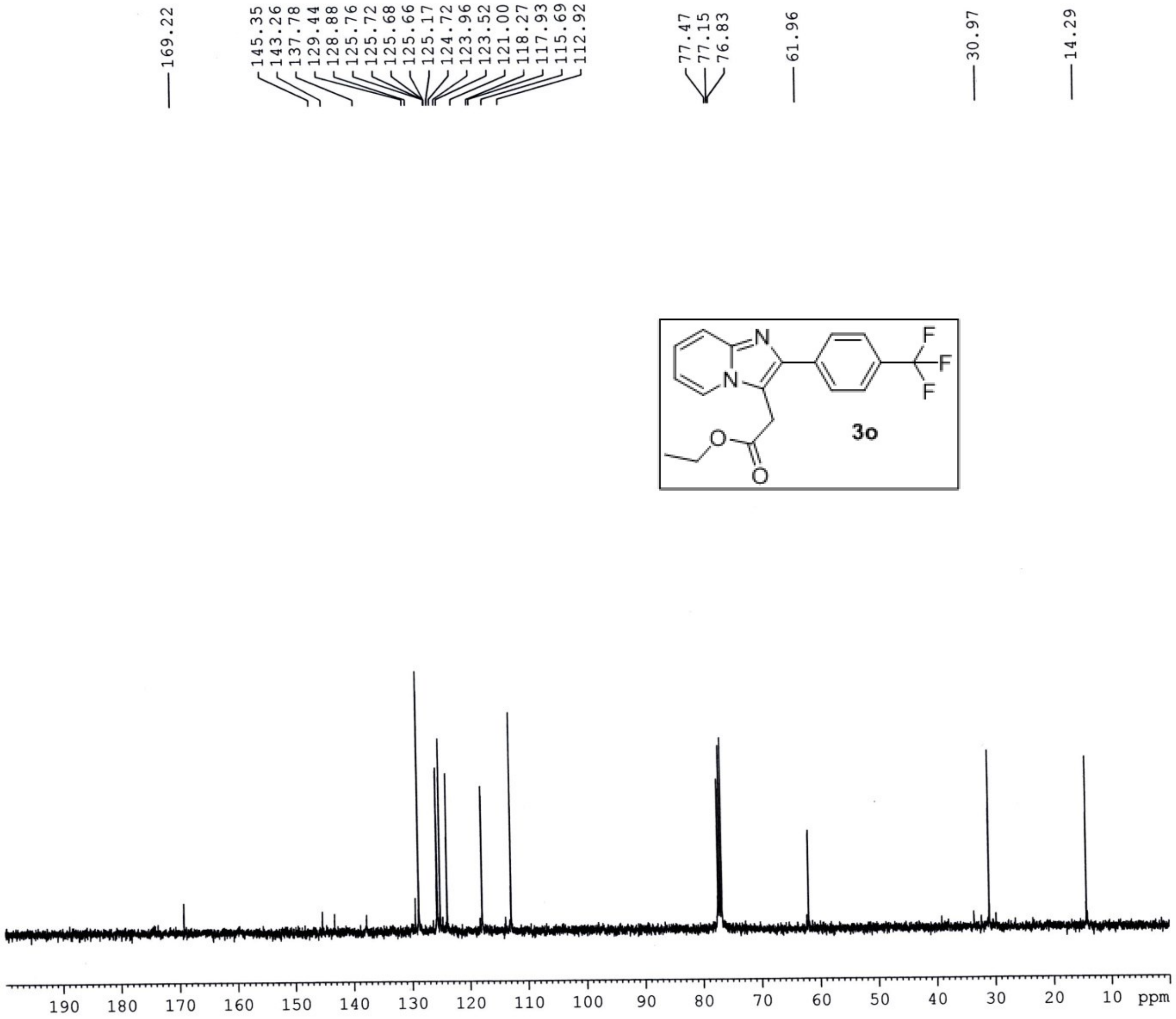
169.22
145.35
143.26
137.78
129.44
128.88
125.76
125.72
125.68
125.66
125.17
124.72
123.96
123.52
121.00
118.27
117.93
115.69
112.92
77.47
77.15
76.83
61.96
30.97
14.29
3o
190 180 170 160 150 140 130 120 110 100 90 80 70 60 50 40 30 20 10 ppm

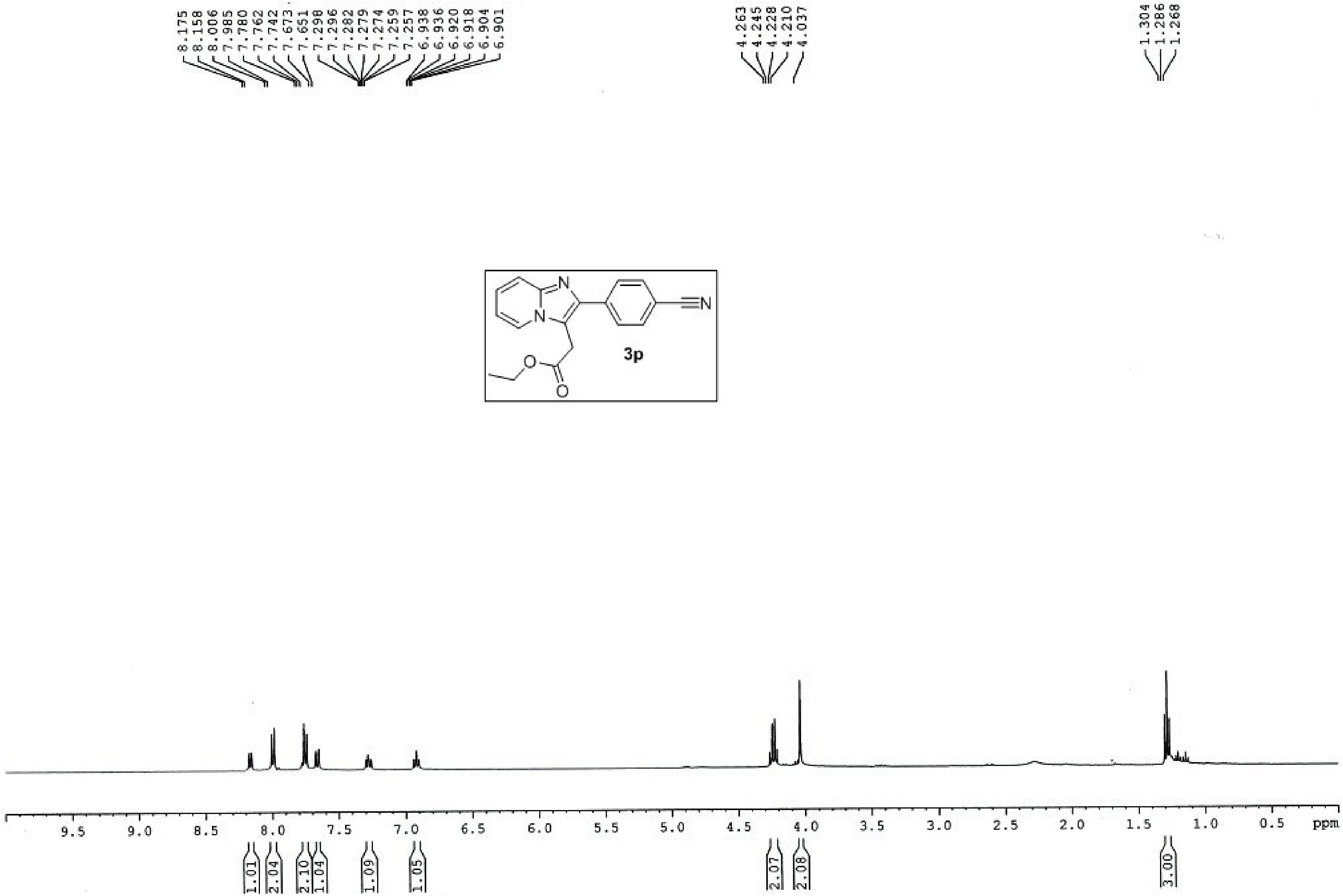
8.175
8.158
8.006
7.985
7.780
7.762
7.742
7.673
7.651
7.298
7.296
7.282
7.279
7.274
7.259
7.257
6.938
6.936
6.920
6.918
6.904
6.901
4.263
4.245
4.228
4.210
4.037
1.304
1.286
1.268
N
N
N
O
O
3p
9.5
9.0
8.5
8.0
7.5
7.0
6.5
6.0
5.5
5.0
4.5
4.0
3.5
3.0
2.5
2.0
1.5
1.0
0.5
ppm
1.01
2.04
2.10
1.04
1.09
1.05
2.07
2.08
3.00

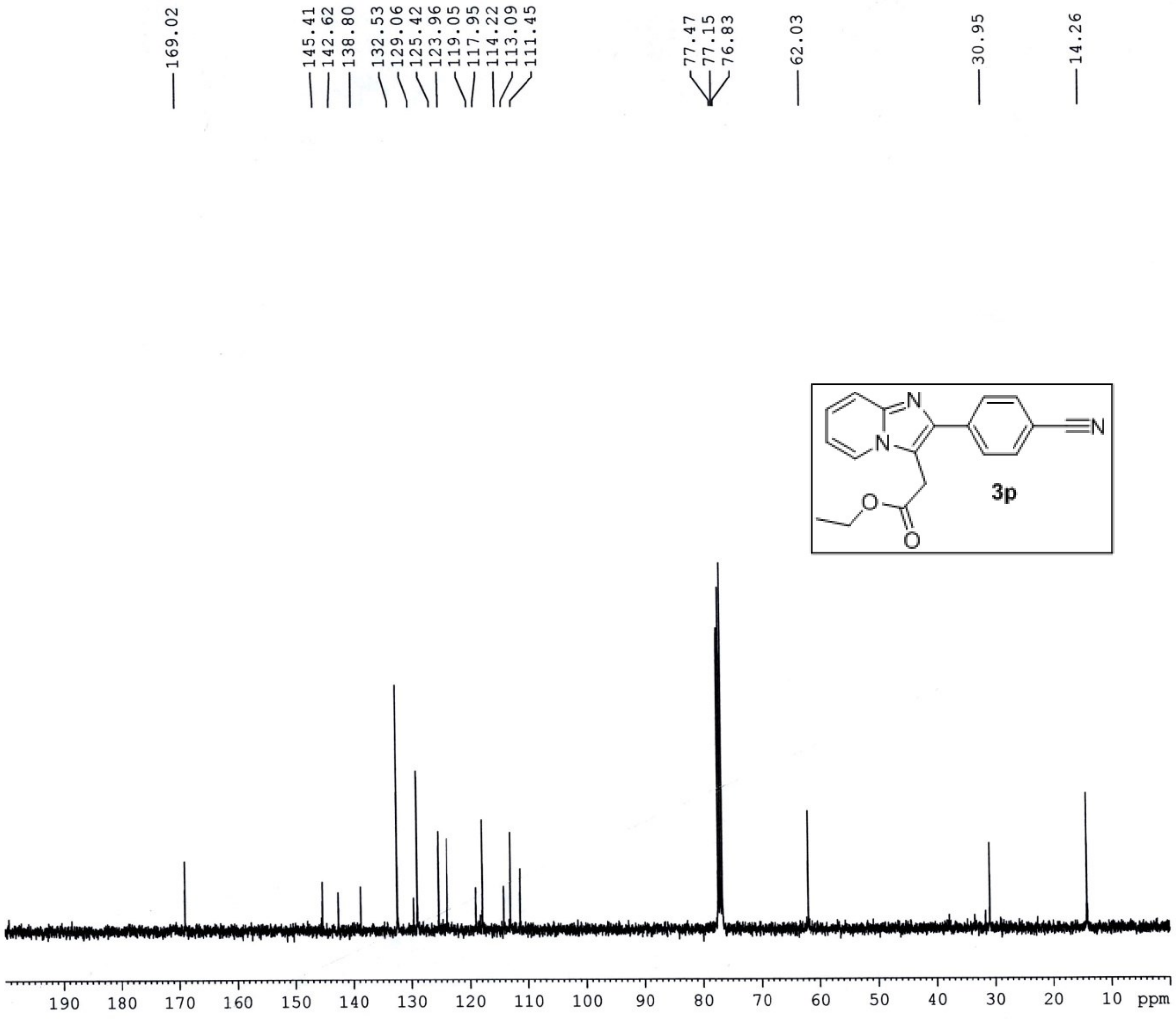
169.02
145.41
142.62
138.80
132.53
129.06
125.42
123.96
119.05
117.95
114.22
113.09
111.45
77.47
77.15
76.83
62.03
30.95
14.26
3p
ppm

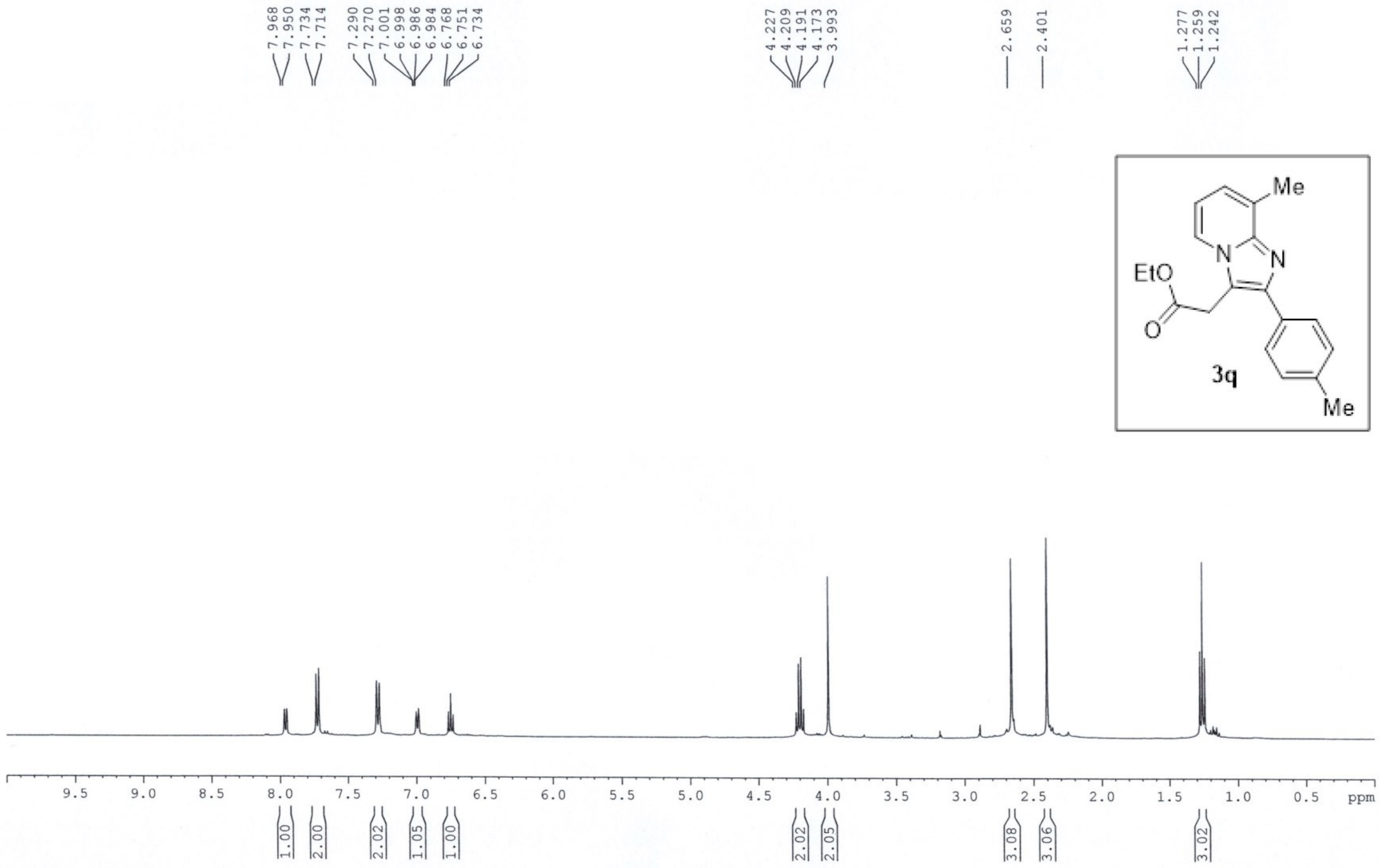
7.968
7.950
7.734
7.714
7.290
7.270
7.001
6.998
6.986
6.984
6.768
6.751
6.734
4.227
4.209
4.191
4.173
3.993
2.659
2.401
1.277
1.259
1.242
Me
N
N
EtO
O
3q
Me
9.5
9.0
8.5
8.0
7.5
7.0
6.5
6.0
5.5
5.0
4.5
4.0
3.5
3.0
2.5
2.0
1.5
1.0
0.5
ppm
1.00
2.00
2.02
1.05
1.00
2.02
2.05
3.08
3.06
3.02

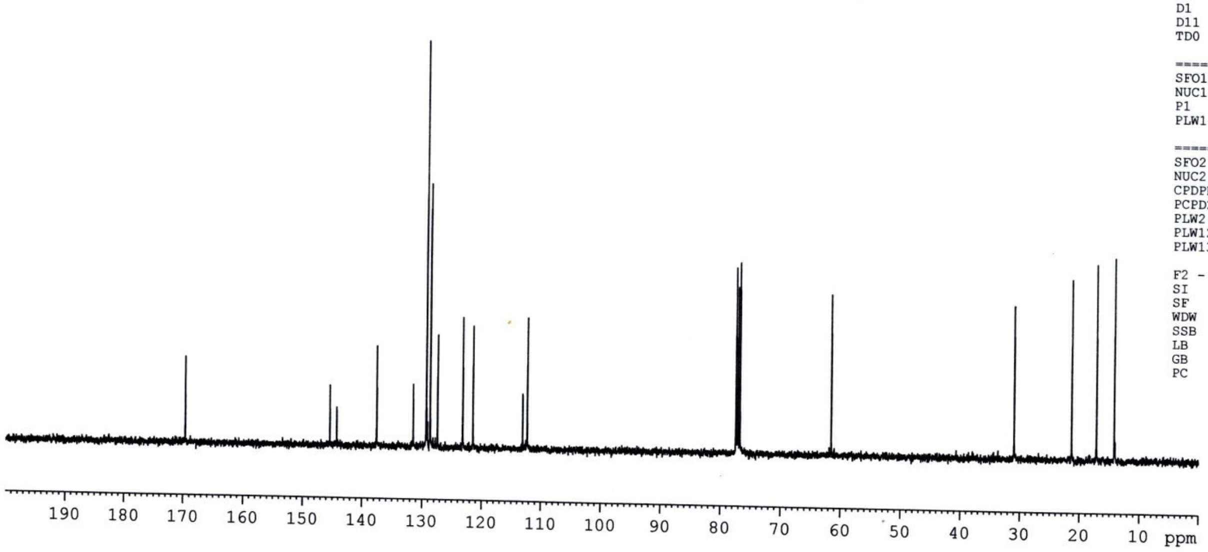

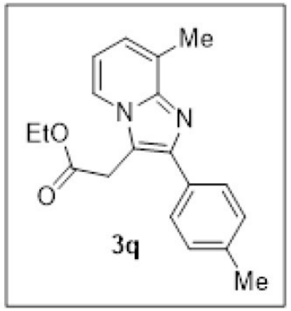

BRUKER

Current Data Parameters
NAME Dr. A HAJRA-2019-13C
EXPNO 428
PROCNO 1

F2 - Acquisition Parameters
Date_ 20191019
Time 10.48
INSTRUM spect
PROBHD 5 mm PABBO BB/
PULPROG zgpg30
TD 32768
SOLVENT CDCl3
NS 120
DS 2
SWH 24038.461 Hz
FIDRES 0.733596 Hz
AQ 0.6815744 sec
RG 186.42
DW 20.800 usec
DE 6.50 usec
TE 298.0 K
D1 2.00000000 sec
D11 0.03000000 sec
TD0 1

======== CHANNEL f1 ========
SFO1 100.6278588 MHz
NUC1 13C
P1 8.90 usec
PLW1 54.00000000 W

======== CHANNEL f2 ========
SFO2 400.1516006 MHz
NUC2 1H
CPDPRG[2 waltz16
PCPD2 90.00 usec
PLW2 12.00000000 W
PLW12 0.32231000 W
PLW13 0.16212000 W

F2 - Processing parameters
SI 16384
SF 100.6177932 MHz
WDW EM
SSB 0
LB 1.00 Hz
GB 0
PC 1.40

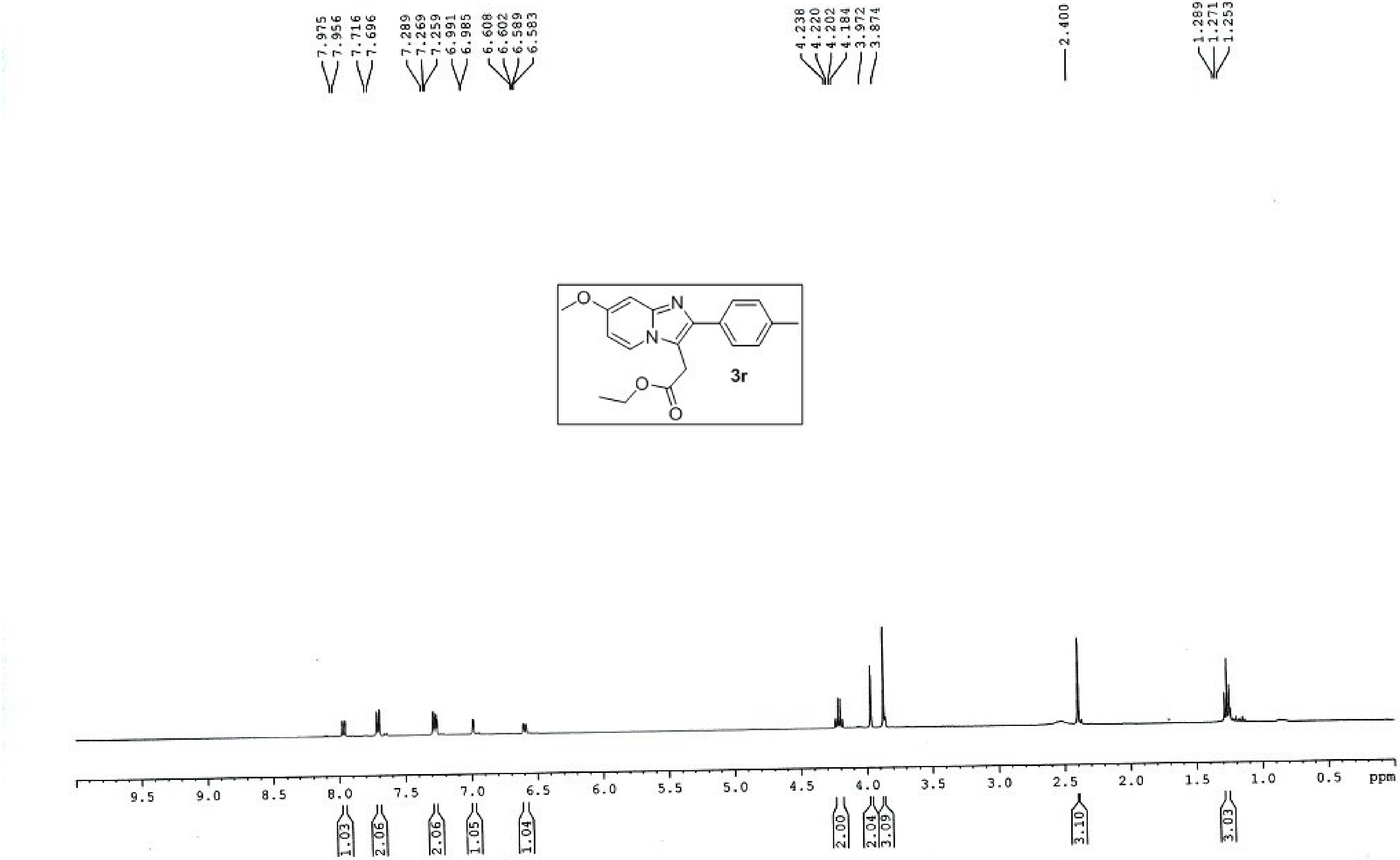
7.975
7.956
7.716
7.696
7.289
7.269
7.259
6.991
6.985
6.608
6.602
6.589
6.583
4.238
4.220
4.202
4.184
3.972
3.874
2.400
1.289
1.271
1.253
N
O
3r
9.5 9.0 8.5 8.0 7.5 7.0 6.5 6.0 5.5 5.0 4.5 4.0 3.5 3.0 2.5 2.0 1.5 1.0 0.5 ppm
1.03
2.06
2.06
1.05
1.04
2.00
2.04
3.09
3.10
3.03

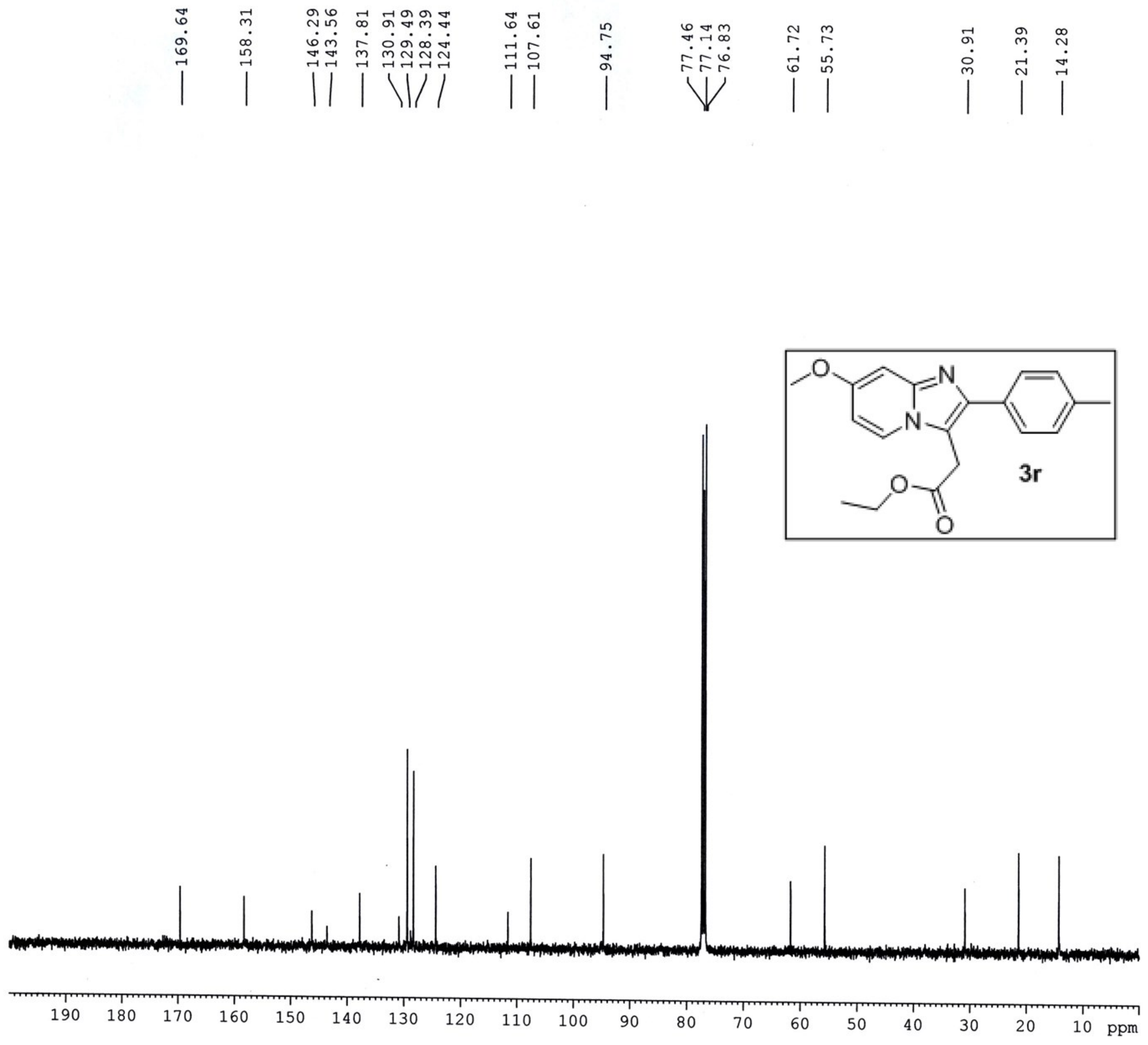
169.64
158.31
146.29
143.56
137.81
130.91
129.49
128.39
124.44
111.64
107.61
94.75
77.46
77.14
76.83
61.72
55.73
30.91
21.39
14.28
3r
190 180 170 160 150 140 130 120 110 100 90 80 70 60 50 40 30 20 10 ppm

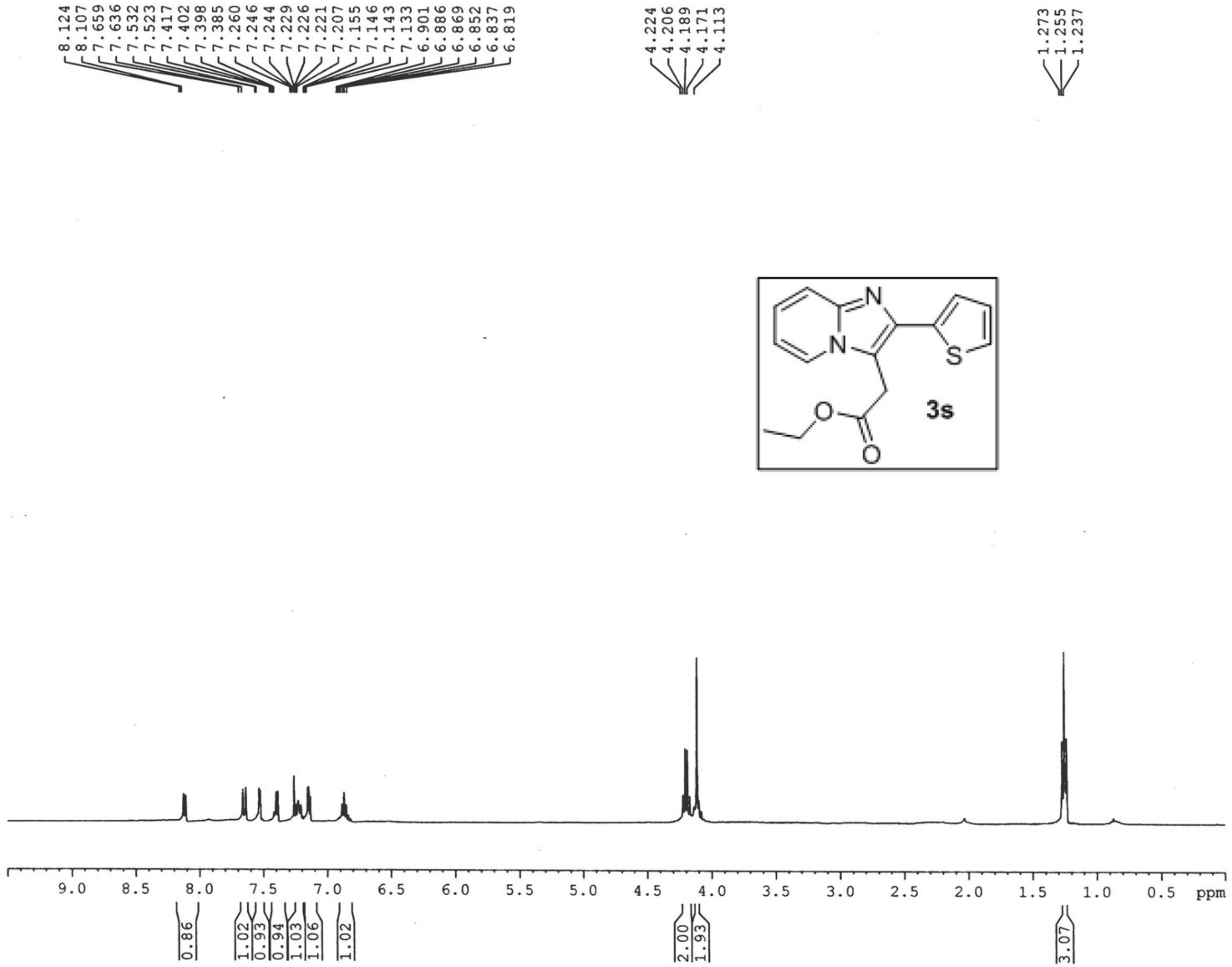
8.124
8.107
7.659
7.636
7.532
7.523
7.417
7.402
7.398
7.385
7.260
7.246
7.244
7.229
7.226
7.221
7.207
7.155
7.146
7.143
7.133
6.901
6.886
6.869
6.852
6.837
6.819
4.224
4.206
4.189
4.171
4.113
1.273
1.255
1.237
3s
9.0 8.5 8.0 7.5 7.0 6.5 6.0 5.5 5.0 4.5 4.0 3.5 3.0 2.5 2.0 1.5 1.0 0.5 ppm
0.86
1.02
0.93
0.94
1.03
1.06
1.02
2.00
1.93
3.07

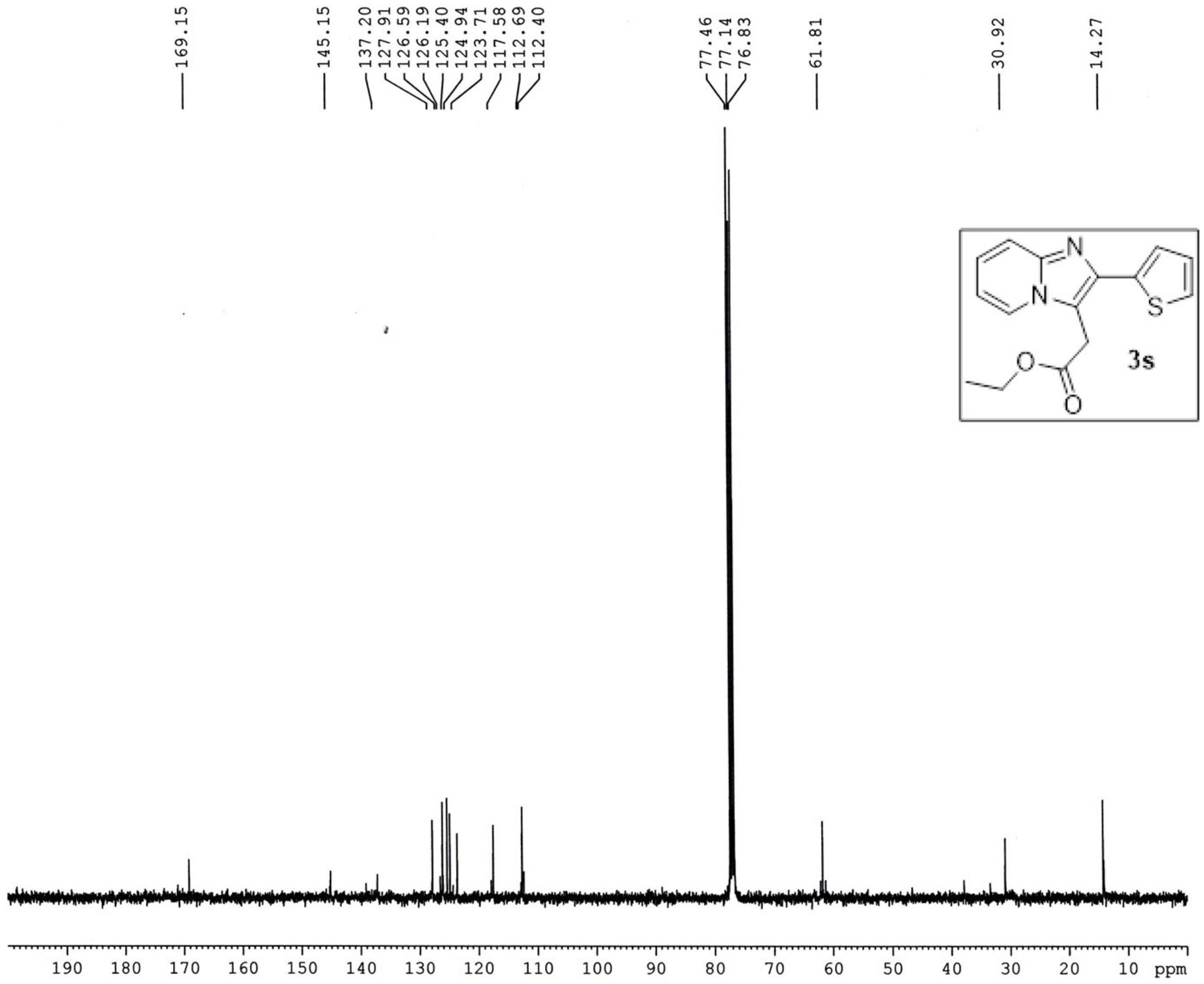
169.15
145.15
137.20
127.91
126.59
126.19
125.40
124.94
123.71
117.58
112.69
112.40
77.46
77.14
76.83
61.81
30.92
14.27
N
N
S
O
O
3s
190 180 170 160 150 140 130 120 110 100 90 80 70 60 50 40 30 20 10 ppm

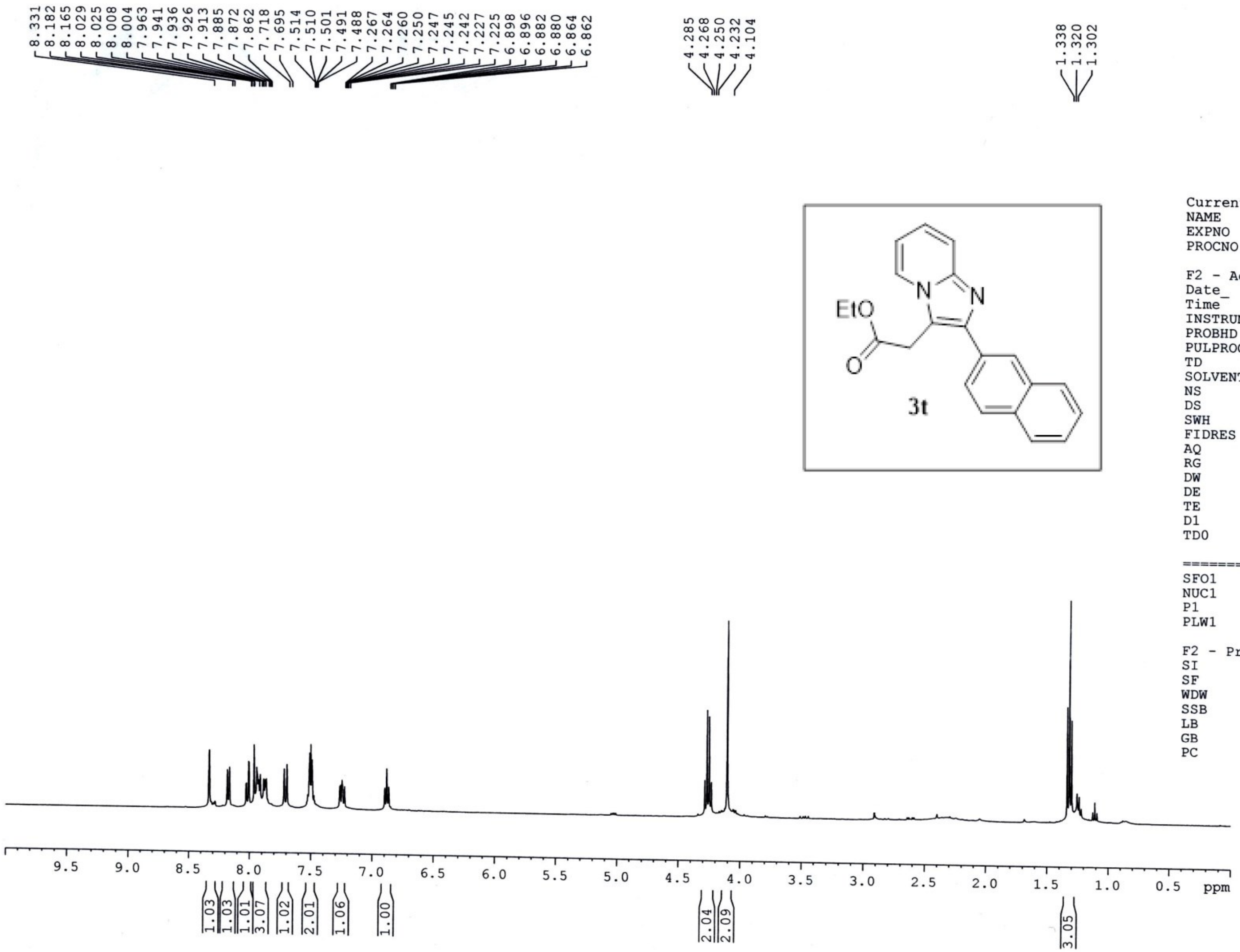

Current Data Parameters
NAME Dr. A HAJRA 2019 1H
EXPNO 874
PROCNO 1

F2 - Acquisition Parameters
Date_ 20191017
Time 17.25
INSTRUM spect
PROBHD 5 mm PABBO BB/
PULPROG zg30
TD 32768
SOLVENT CDCl3
NS 8
DS 2
SWH 8223.685 Hz
FIDRES 0.250967 Hz
AQ 1.9922944 sec
RG 67.81
DW 60.800 usec
DE 6.50 usec
TE 297.1 K
D1 1.00000000 sec
TD0 1

======== CHANNEL f1 ========
SFO1 400.1524711 MHz
NUC1 1H
P1 14.75 usec
PLW1 12.00000000 W

F2 - Processing parameters
SI 16384
SF 400.1500092 MHz
WDW EM
SSB 0
LB 0.30 Hz
GB 0
PC 2.00

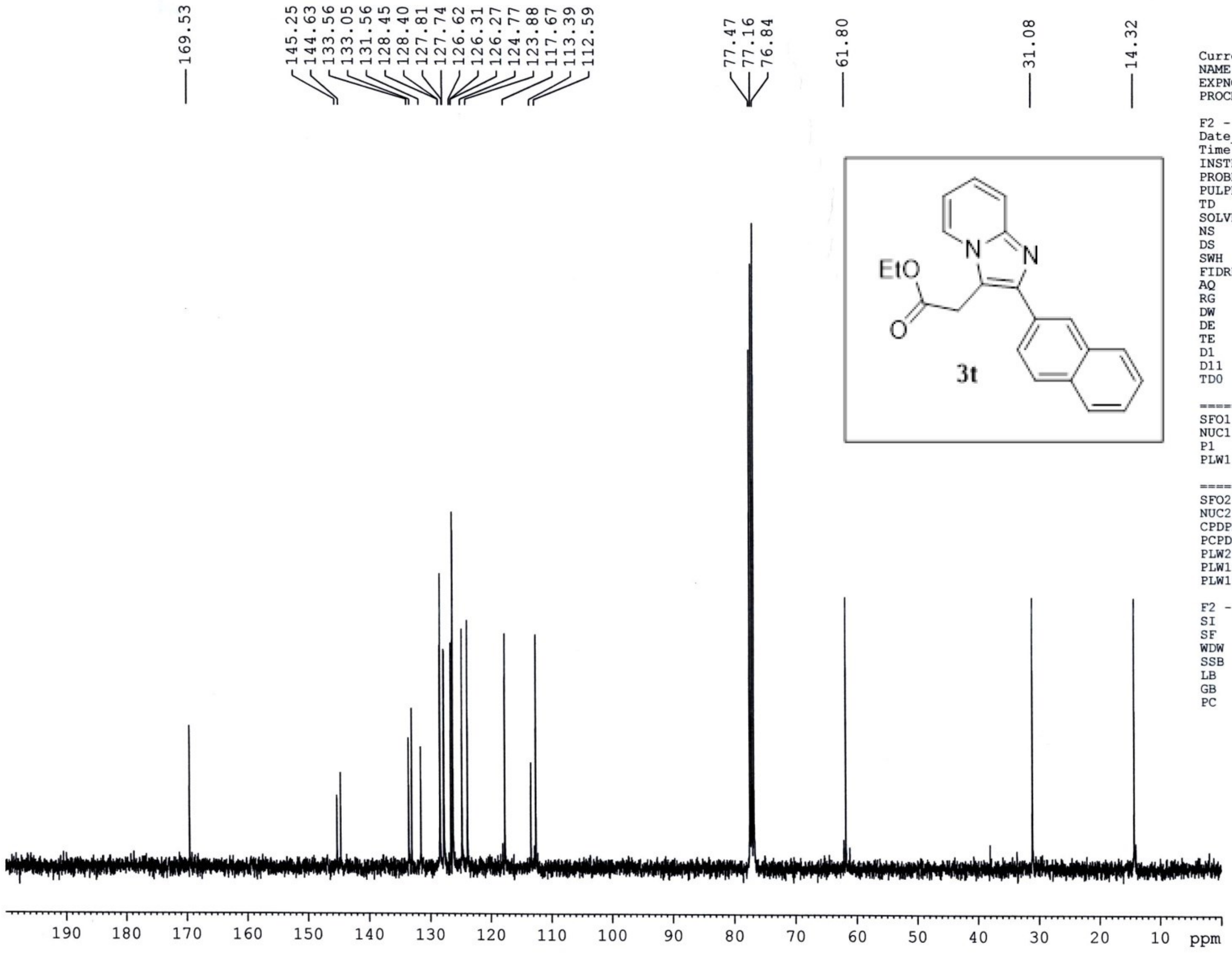


```
Current Data Parameters
NAME      Dr. A HAJRA-2019-13(
EXPNO                   417
PROCNO                    1

F2 - Acquisition Parameters
Date_              20191017
Time                  17.38
INSTRUM               spect
PROBHD     5 mm PABBO BB/
PULPROG              zgpg30
TD                    32768
SOLVENT               CDCl3
NS                      240
DS                        2
SWH               24038.461 Hz
FIDRES             0.733596 Hz
AQ                0.6815744 sec
RG                   186.42
DW                   20.800 usec
DE                     6.50 usec
TE                    297.7 K
D1               2.00000000 sec
D11              0.03000000 sec
TD0                       1

======== CHANNEL f1 ========
SFO1            100.6278588 MHz
NUC1                    13C
P1                     8.90 usec
PLW1            54.00000000 W

======== CHANNEL f2 ========
SFO2            400.1516006 MHz
NUC2                     1H
CPDPRG[2            waltz16
PCPD2                 90.00 usec
PLW2            12.00000000 W
PLW12            0.32231000 W
PLW13            0.16212000 W

F2 - Processing parameters
SI                    16384
SF              100.6177897 MHz
WDW                      EM
SSB       0
LB                     1.00 Hz
GB        0
PC                     1.40
```

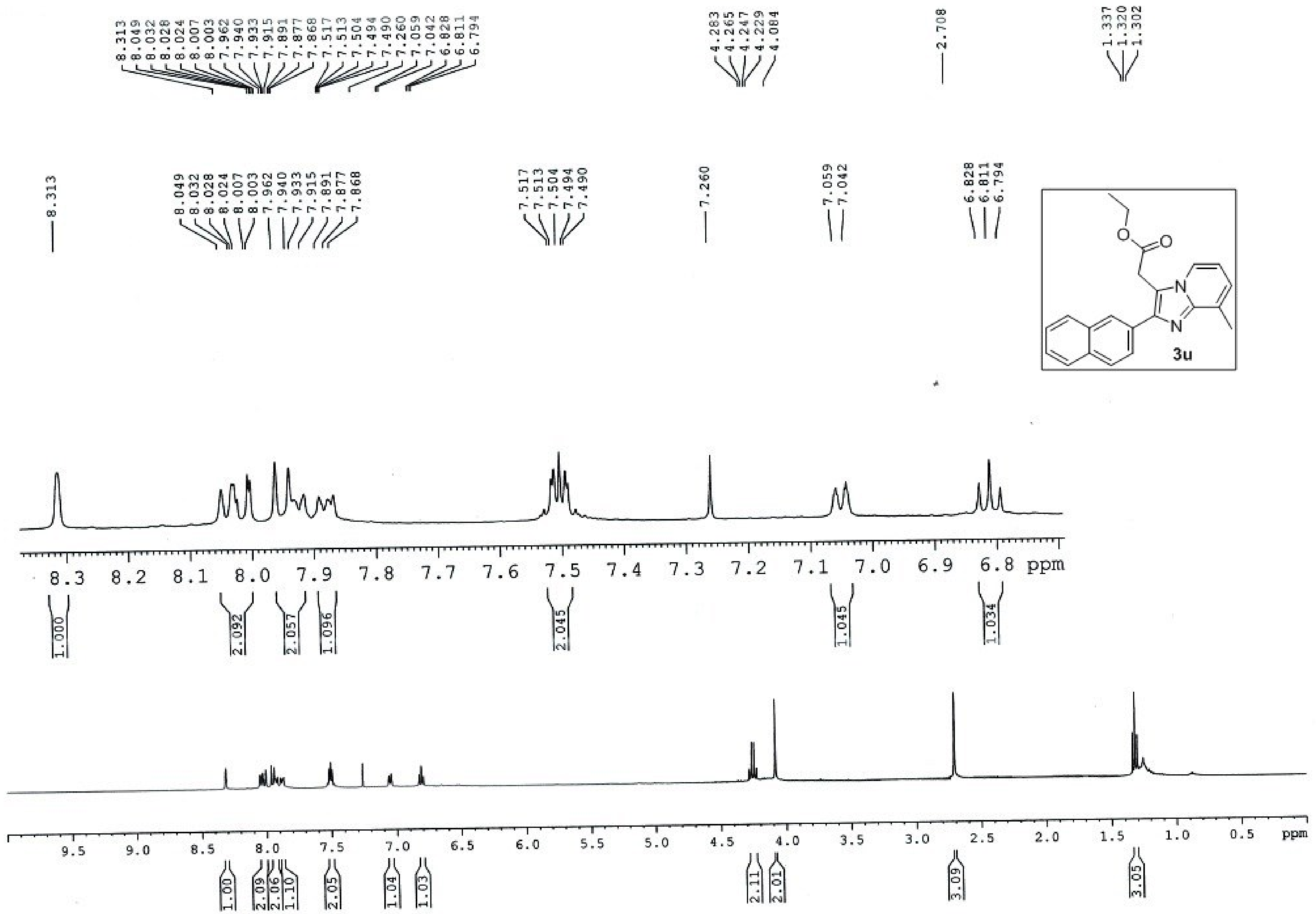
8.313 8.049 8.032 8.028 8.024 8.007 8.003 7.962 7.940 7.933 7.915 7.891 7.877 7.868 7.517 7.513 7.504 7.494 7.490 7.260 7.059 7.042 6.828 6.811 6.794
4.283 4.265 4.247 4.229 4.084
2.708
1.337 1.320 1.302
3u
ppm
1.000 2.092 2.057 1.096 2.045 1.045 1.034
1.00 2.09 2.06 1.10 2.05 1.04 1.03 2.11 2.01 3.09 3.05

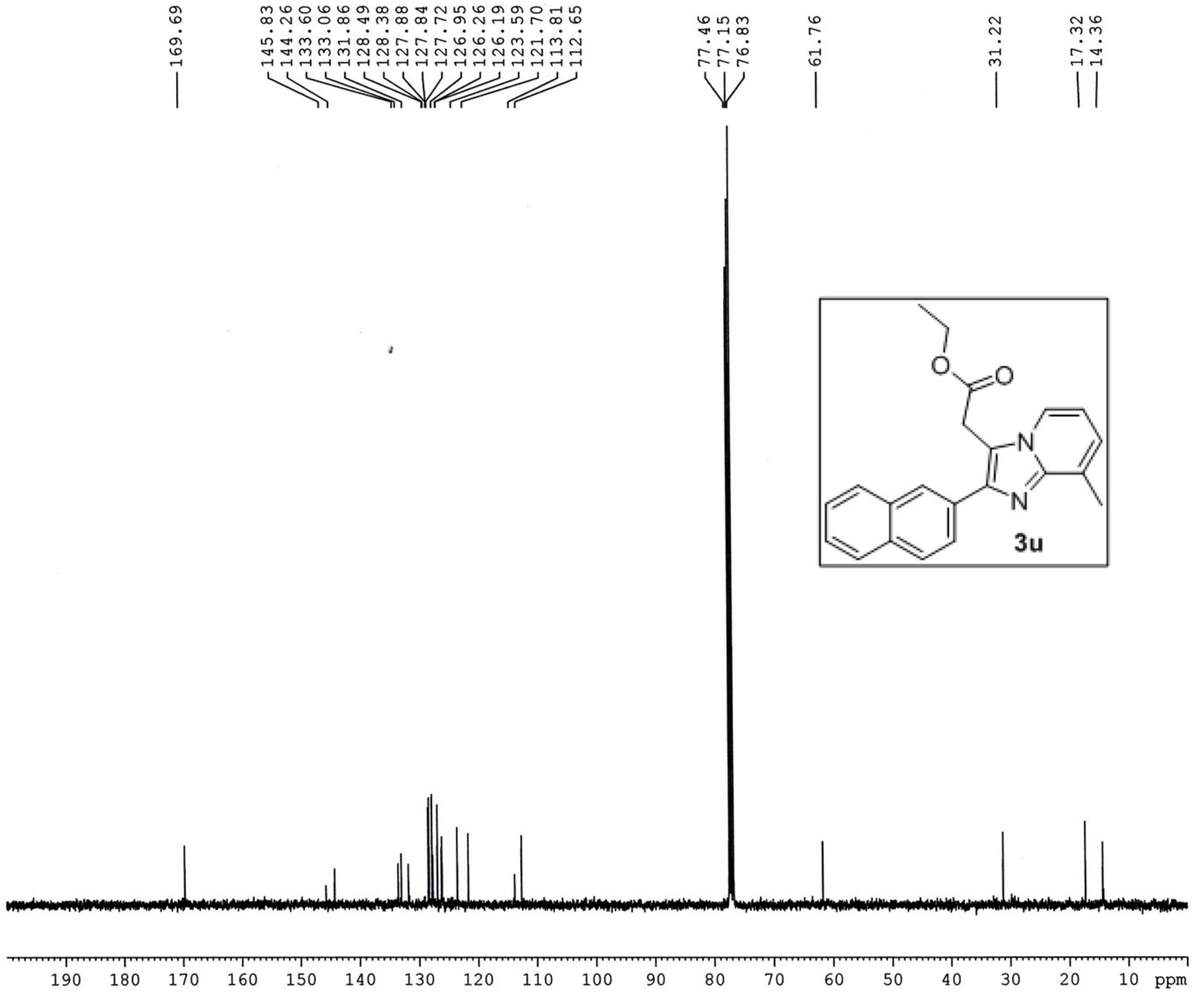
169.69
145.83
144.26
133.60
133.06
131.86
128.49
128.38
127.88
127.84
127.72
126.95
126.26
126.19
123.59
121.70
113.81
112.65
77.46
77.15
76.83
61.76
31.22
17.32
14.36
3u
190 180 170 160 150 140 130 120 110 100 90 80 70 60 50 40 30 20 10 ppm

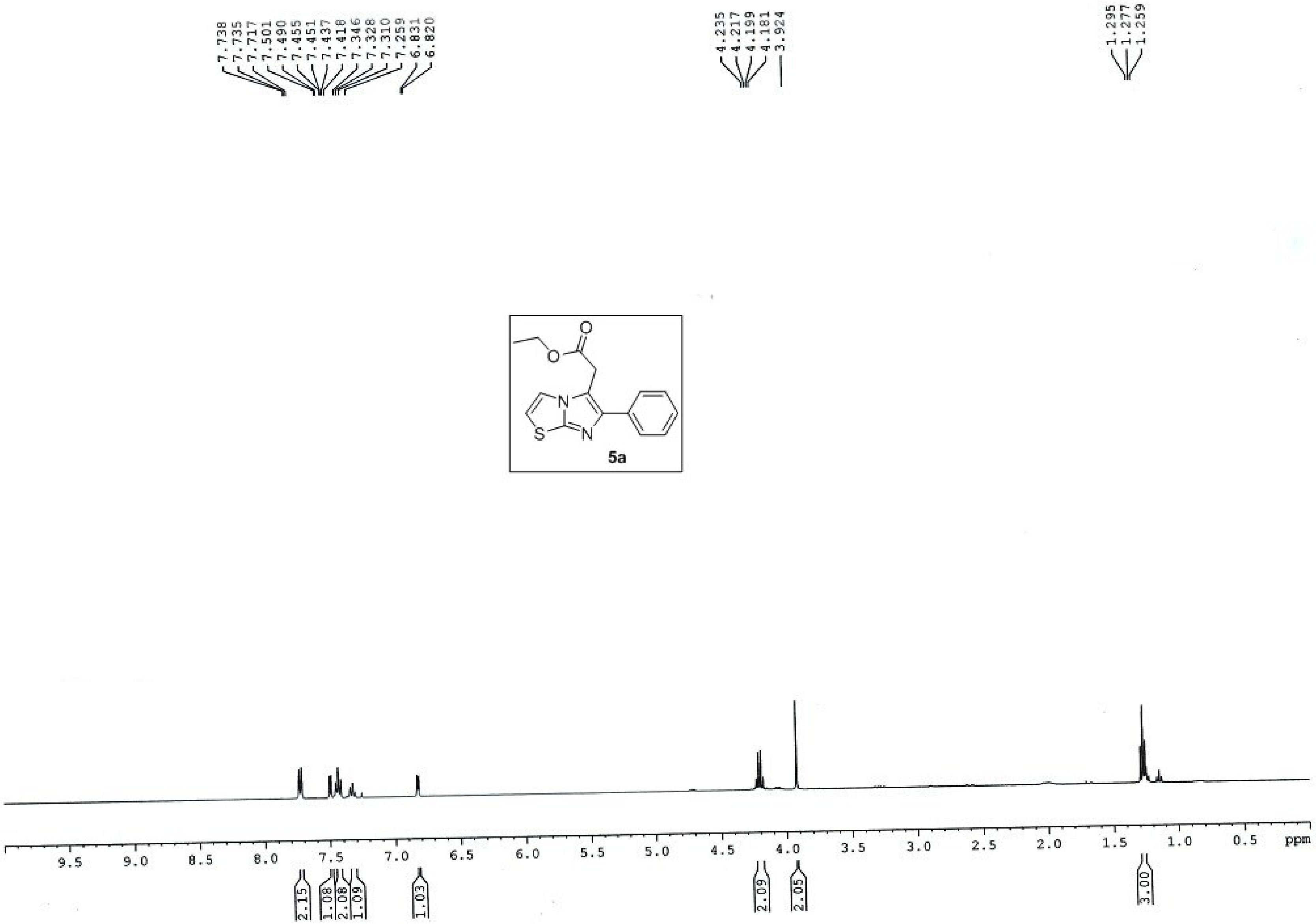
7.738
7.735
7.717
7.501
7.490
7.455
7.451
7.437
7.418
7.346
7.328
7.310
7.259
6.831
6.820
4.235
4.217
4.199
4.181
3.924
1.295
1.277
1.259
5a
9.5 9.0 8.5 8.0 7.5 7.0 6.5 6.0 5.5 5.0 4.5 4.0 3.5 3.0 2.5 2.0 1.5 1.0 0.5 ppm
2.15
1.08
2.08
1.09
1.03
2.09
2.05
3.00

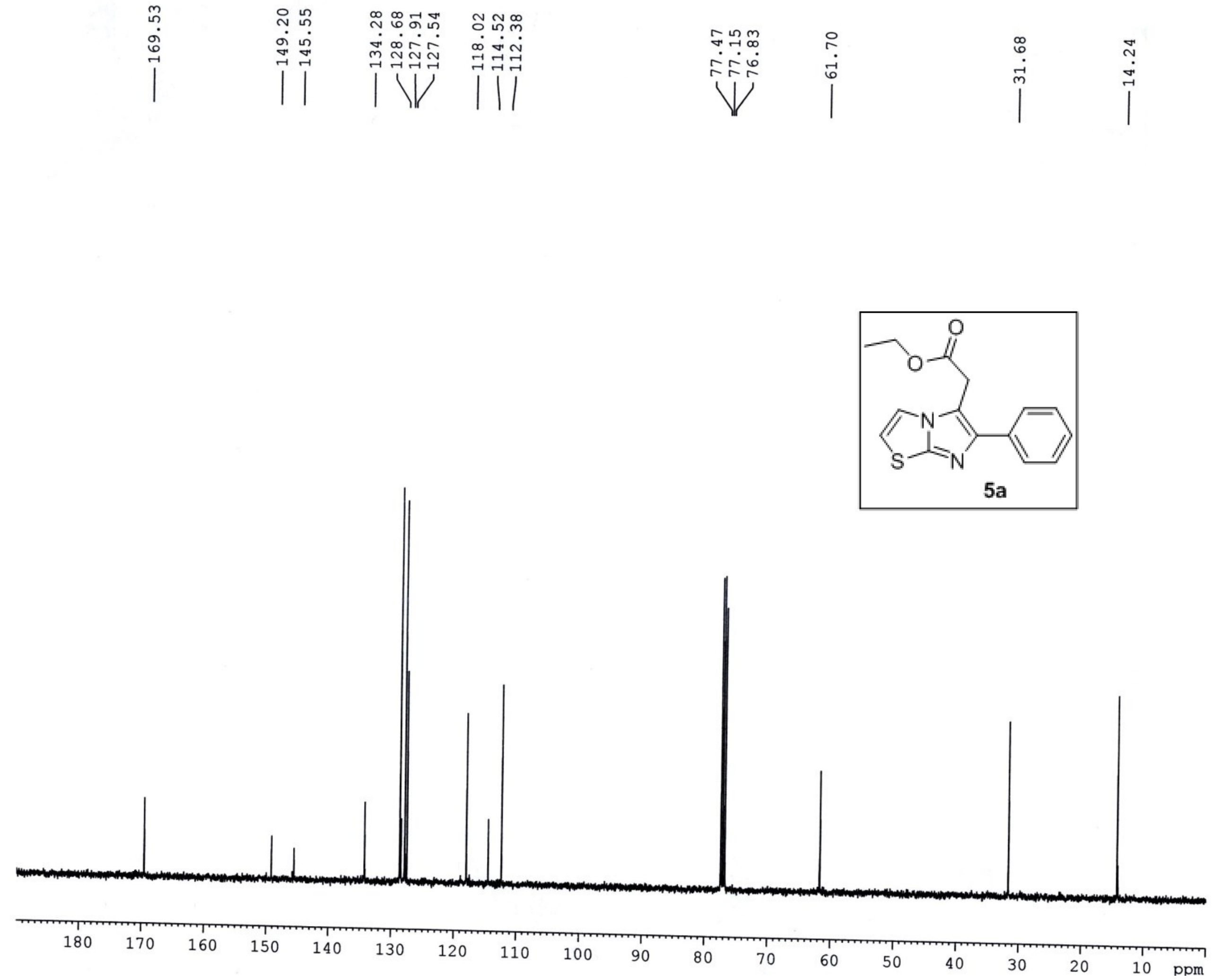
169.53
149.20
145.55
134.28
128.68
127.91
127.54
118.02
114.52
112.38
77.47
77.15
76.83
61.70
31.68
14.24
O
O
N
S
N
5a
180
170
160
150
140
130
120
110
100
90
80
70
60
50
40
30
20
10
ppm

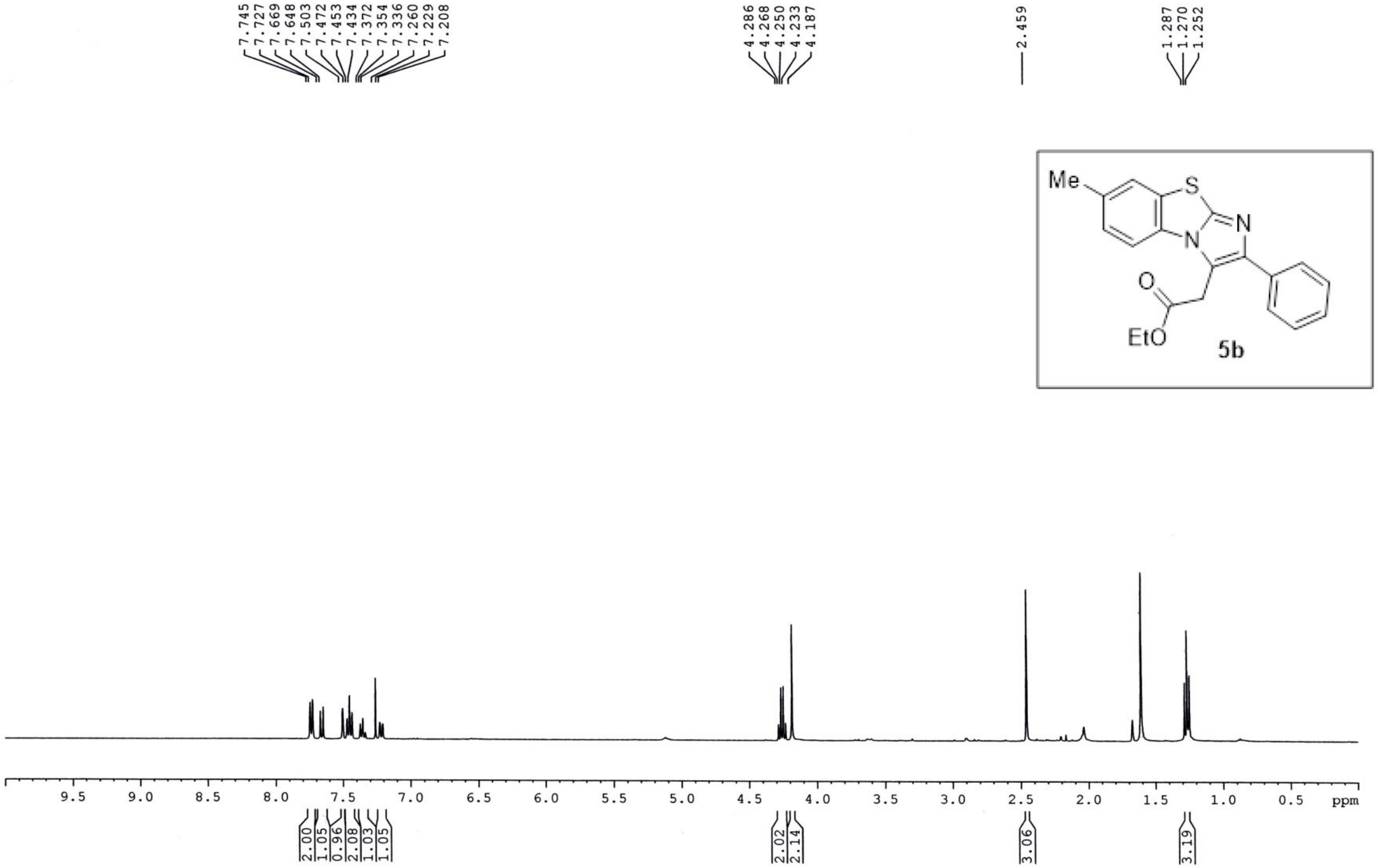
7.745
7.727
7.669
7.648
7.503
7.472
7.453
7.434
7.372
7.354
7.336
7.260
7.229
7.208
4.286
4.268
4.250
4.233
4.187
2.459
1.287
1.270
1.252
Me
S
N
N
O
EtO
5b
9.5
9.0
8.5
8.0
7.5
7.0
6.5
6.0
5.5
5.0
4.5
4.0
3.5
3.0
2.5
2.0
1.5
1.0
0.5
ppm
2.00
1.05
0.96
2.08
1.03
1.05
2.02
2.14
3.06
3.19

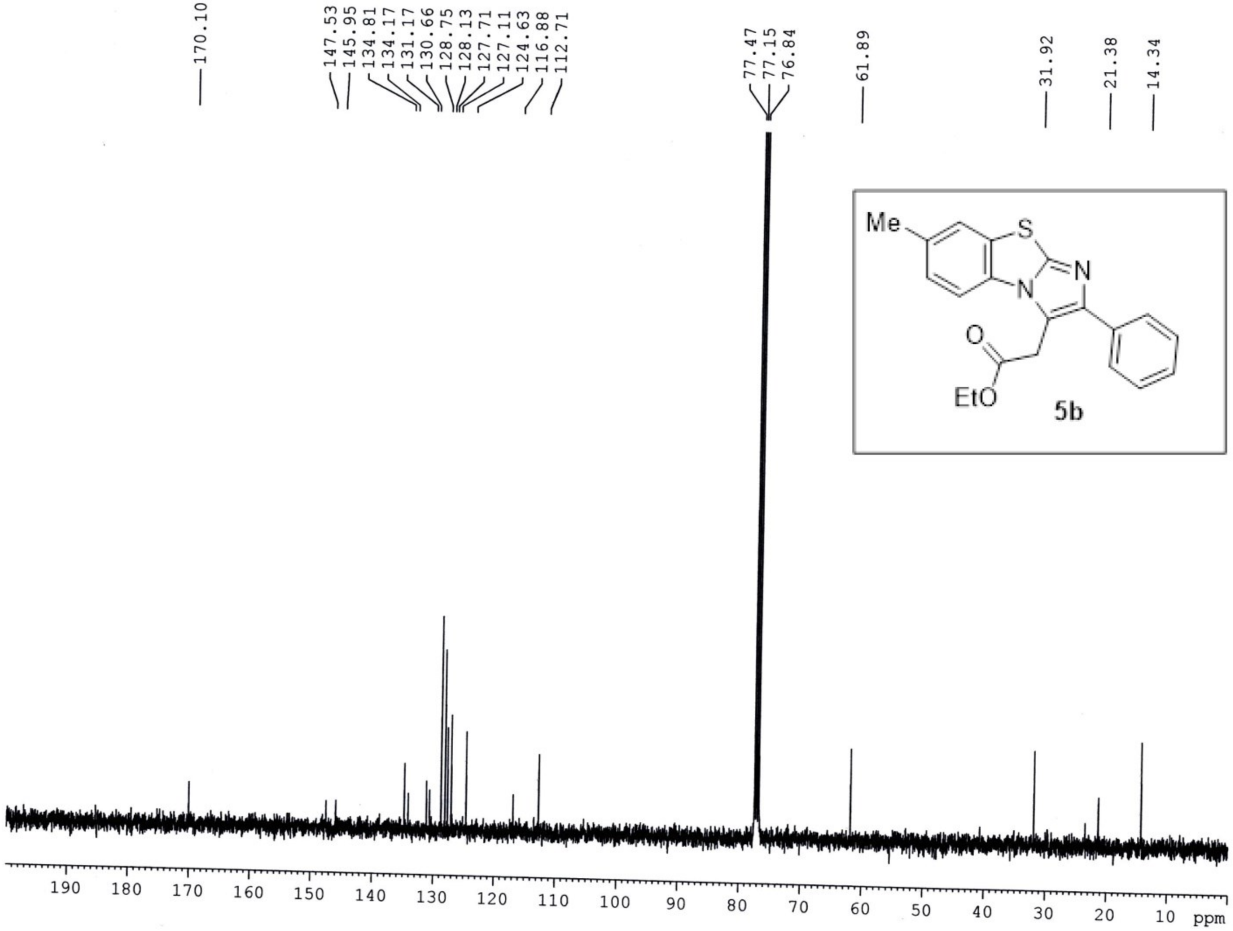

Current Data Parameters
NAME Dr. A HAJRA-2019-13C
EXPNO 484
PROCNO 1

F2 - Acquisition Parameters
Date_ 20191031
Time 21.26
INSTRUM spect
PROBHD 5 mm PABBO BB/
PULPROG zgpg30
TD 32768
SOLVENT CDCl3
NS 640
DS 2
SWH 24038.461 Hz
FIDRES 0.733596 Hz
AQ 0.6815744 sec
RG 186.42
DW 20.800 usec
DE 6.50 usec
TE 297.3 K
D1 2.00000000 sec
D11 0.03000000 sec
TD0 1

======== CHANNEL f1 ========
SFO1 100.6278588 MHz
NUC1 13C
P1 8.90 usec
PLW1 54.00000000 W

======== CHANNEL f2 ========
SFO2 400.1516006 MHz
NUC2 1H
CPDPRG[2 waltz16
PCPD2 90.00 usec
PLW2 12.00000000 W
PLW12 0.32231000 W
PLW13 0.16212000 W

F2 - Processing parameters
SI 16384
SF 100.6177841 MHz
WDW EM
SSB 0
LB 1.00 Hz
GB 0
PC 1.40

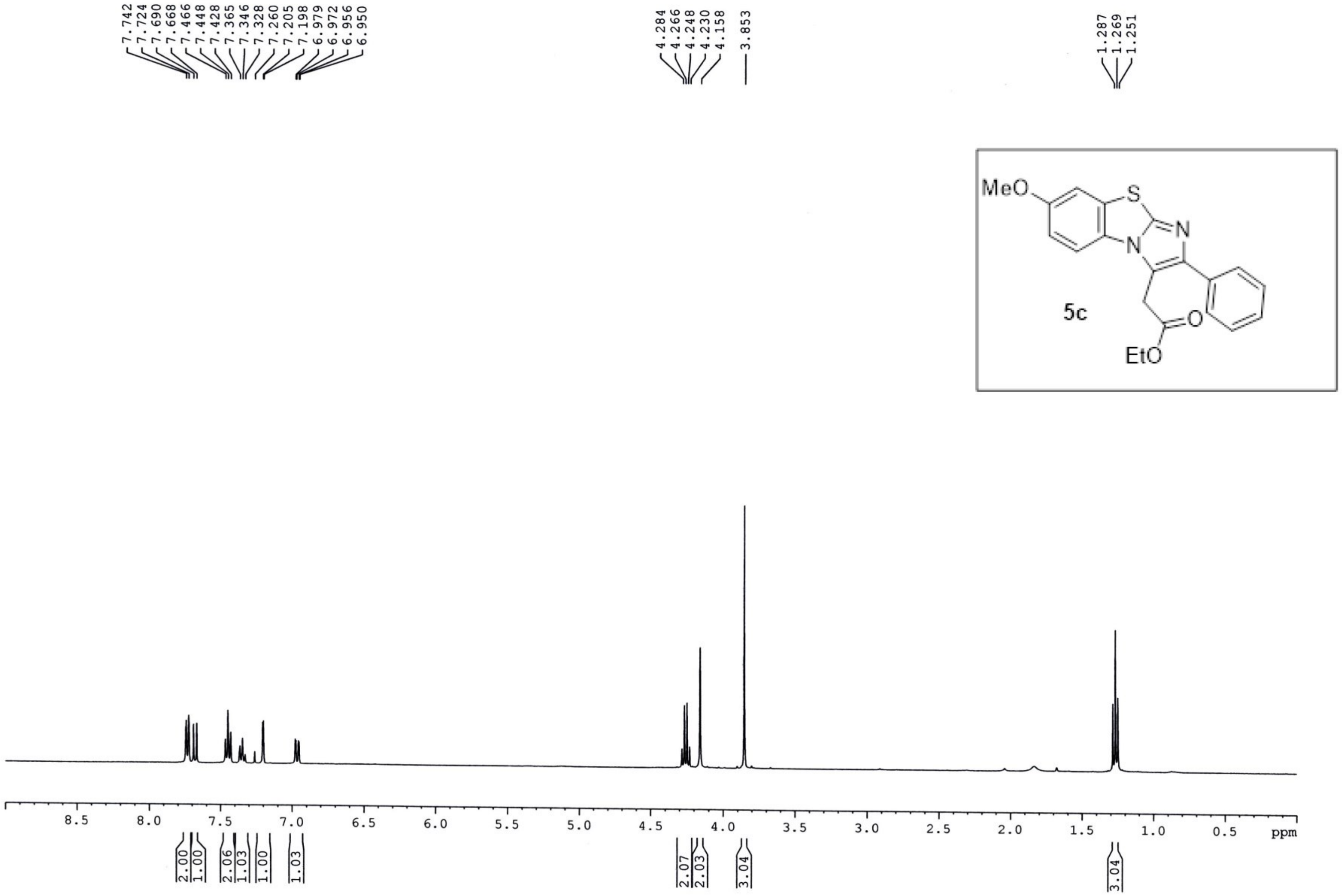
7.742
7.724
7.690
7.668
7.466
7.448
7.428
7.365
7.346
7.328
7.260
7.205
7.198
6.979
6.972
6.956
6.950
4.284
4.266
4.248
4.230
4.158
3.853
1.287
1.269
1.251
MeO
S
N
N
5c
O
EtO
8.5
8.0
7.5
7.0
6.5
6.0
5.5
5.0
4.5
4.0
3.5
3.0
2.5
2.0
1.5
1.0
0.5
ppm
2.00
1.00
2.06
1.03
1.00
1.03
2.07
2.03
3.04
3.04

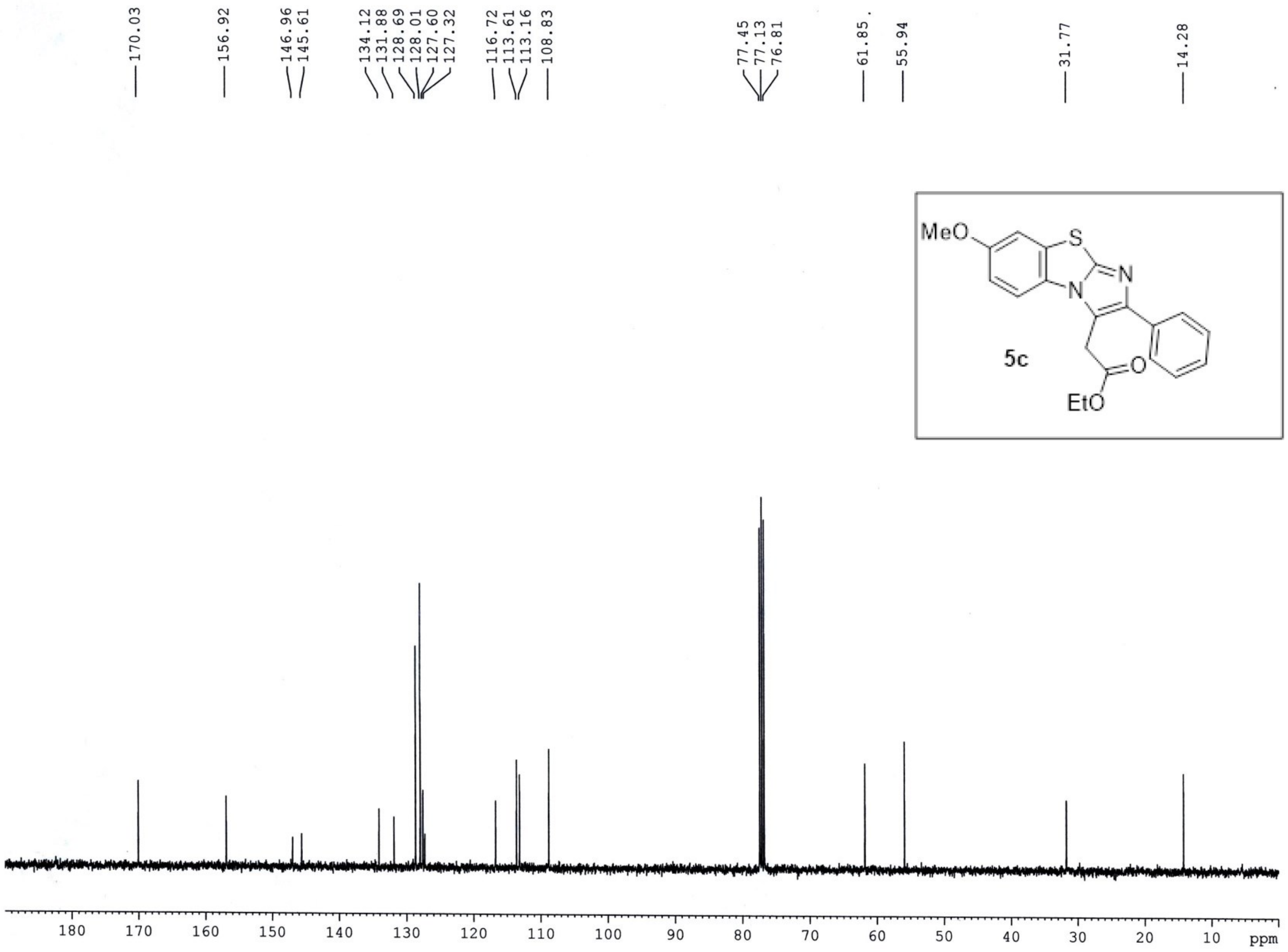

170.03
156.92
146.96
145.61
134.12
131.88
128.69
128.01
127.60
127.32
116.72
113.61
113.16
108.83
77.45
77.13
76.81
61.85
55.94
31.77
14.28
MeO
S
N
N
5c
O
EtO
180
170
160
150
140
130
120
110
100
90
80
70
60
50
40
30
20
10
ppm

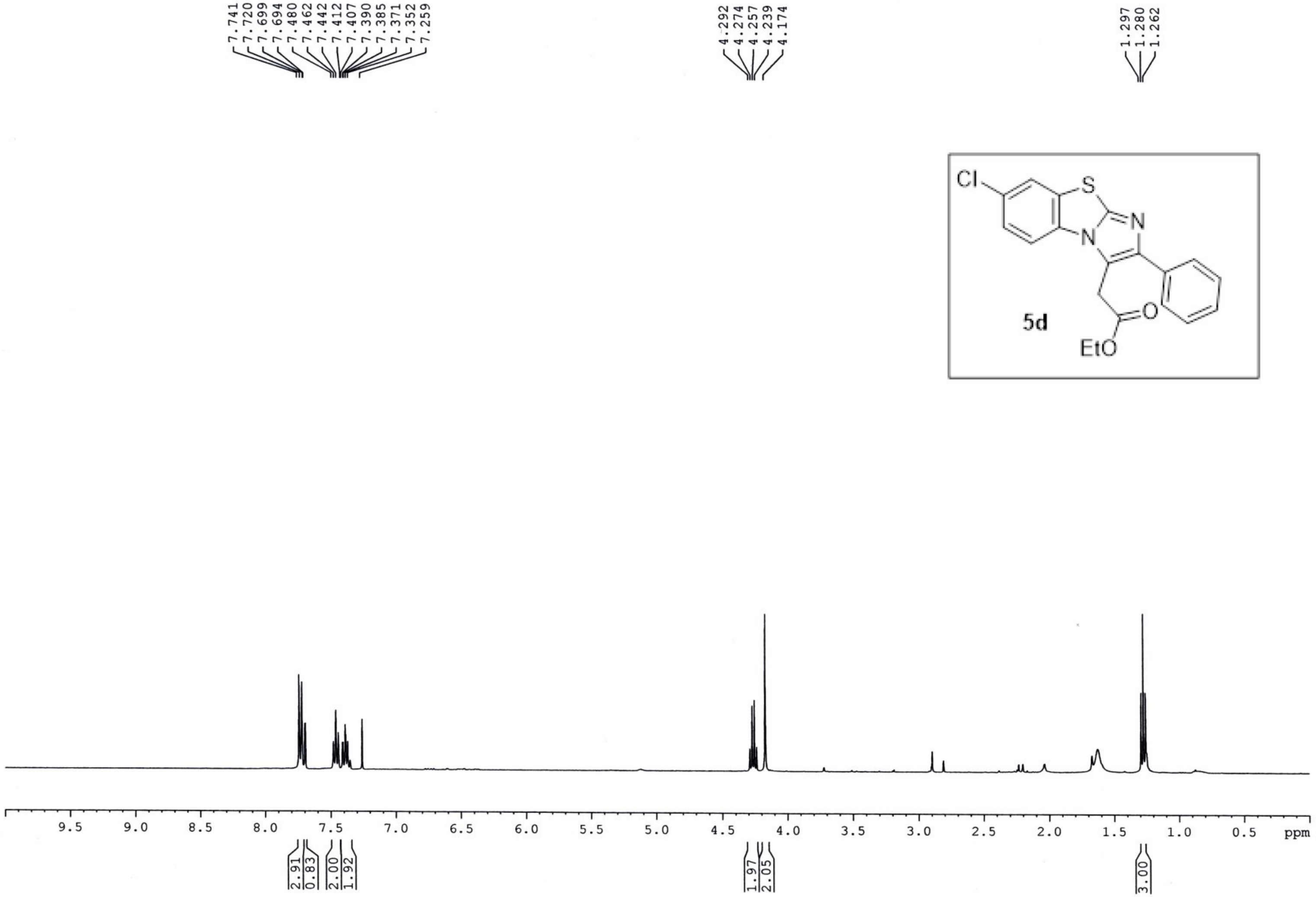
7.741
7.720
7.699
7.694
7.480
7.462
7.442
7.412
7.407
7.390
7.385
7.371
7.352
7.259
4.292
4.274
4.257
4.239
4.174
1.297
1.280
1.262
Cl
S
N
N
O
EtO
5d
9.5 9.0 8.5 8.0 7.5 7.0 6.5 6.0 5.5 5.0 4.5 4.0 3.5 3.0 2.5 2.0 1.5 1.0 0.5 ppm
2.91
0.83
2.00
1.92
1.97
2.05
3.00

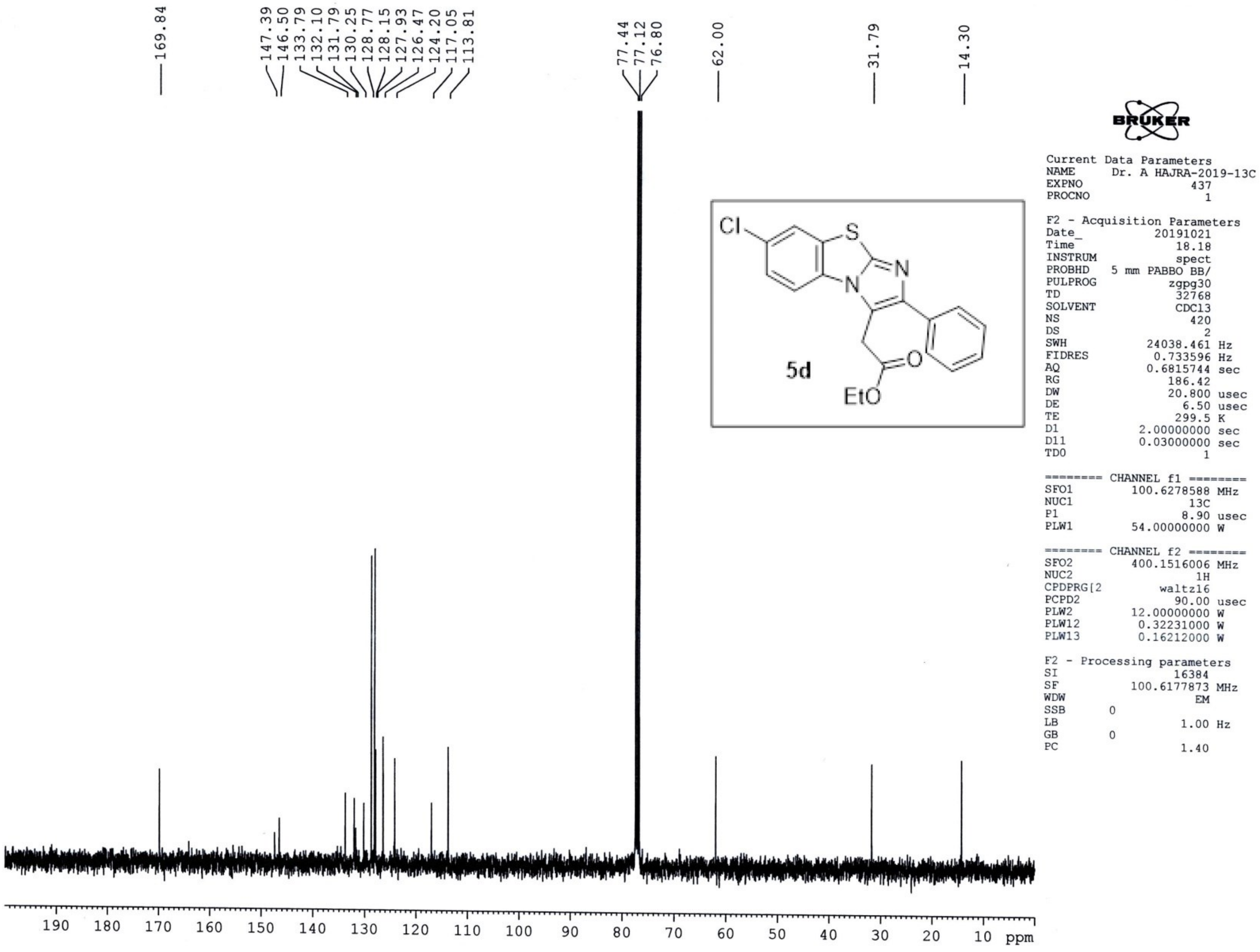
169.84
147.39
146.50
133.79
132.10
131.79
130.25
128.77
128.15
127.93
126.47
124.20
117.05
113.81
77.44
77.12
76.80
62.00
31.79
14.30
Cl
S
N
N
5d
O
EtO
BRUKER
Current Data Parameters
NAME Dr. A HAJRA-2019-13C
EXPNO 437
PROCNO 1
F2 - Acquisition Parameters
Date_ 20191021
Time 18.18
INSTRUM spect
PROBHD 5 mm PABBO BB/
PULPROG zgpg30
TD 32768
SOLVENT CDCl3
NS 420
DS 2
SWH 24038.461 Hz
FIDRES 0.733596 Hz
AQ 0.6815744 sec
RG 186.42
DW 20.800 usec
DE 6.50 usec
TE 299.5 K
D1 2.00000000 sec
D11 0.03000000 sec
TD0 1
======== CHANNEL f1 ========
SFO1 100.6278588 MHz
NUC1 13C
P1 8.90 usec
PLW1 54.00000000 W
======== CHANNEL f2 ========
SFO2 400.1516006 MHz
NUC2 1H
CPDPRG[2 waltz16
PCPD2 90.00 usec
PLW2 12.00000000 W
PLW12 0.32231000 W
PLW13 0.16212000 W
F2 - Processing parameters
SI 16384
SF 100.6177873 MHz
WDW EM
SSB 0
LB 1.00 Hz
GB 0
PC 1.40
190 180 170 160 150 140 130 120 110 100 90 80 70 60 50 40 30 20 10 ppm

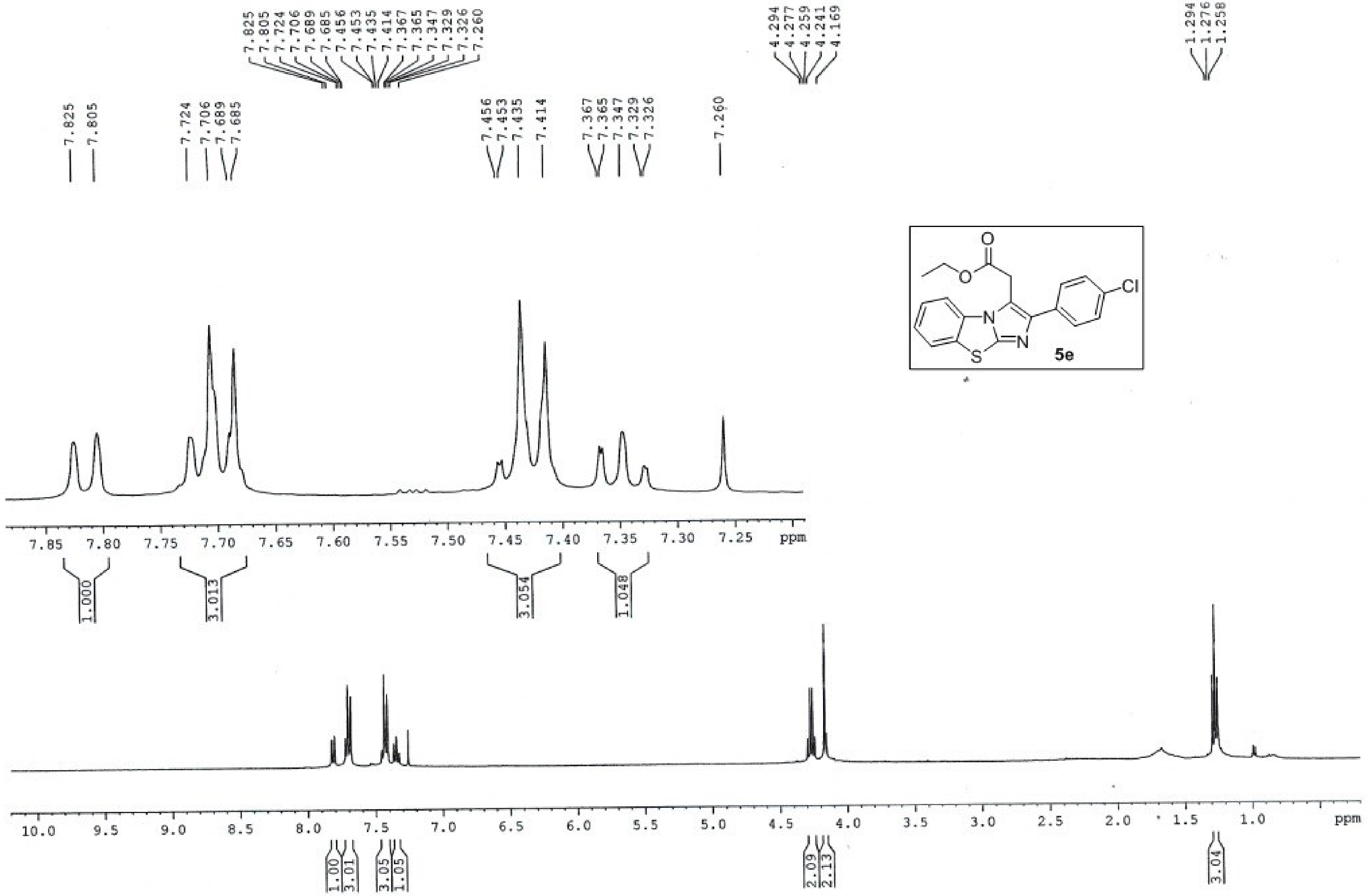
7.825
7.805
7.724
7.706
7.689
7.685
7.456
7.453
7.435
7.414
7.367
7.365
7.347
7.329
7.326
7.260
4.294
4.277
4.259
4.241
4.169
1.294
1.276
1.258
O
O
N
S
N
Cl
5e
1.000
3.013
3.054
1.048
ppm
1.00
3.01
3.05
1.05
2.09
2.13
3.04
ppm

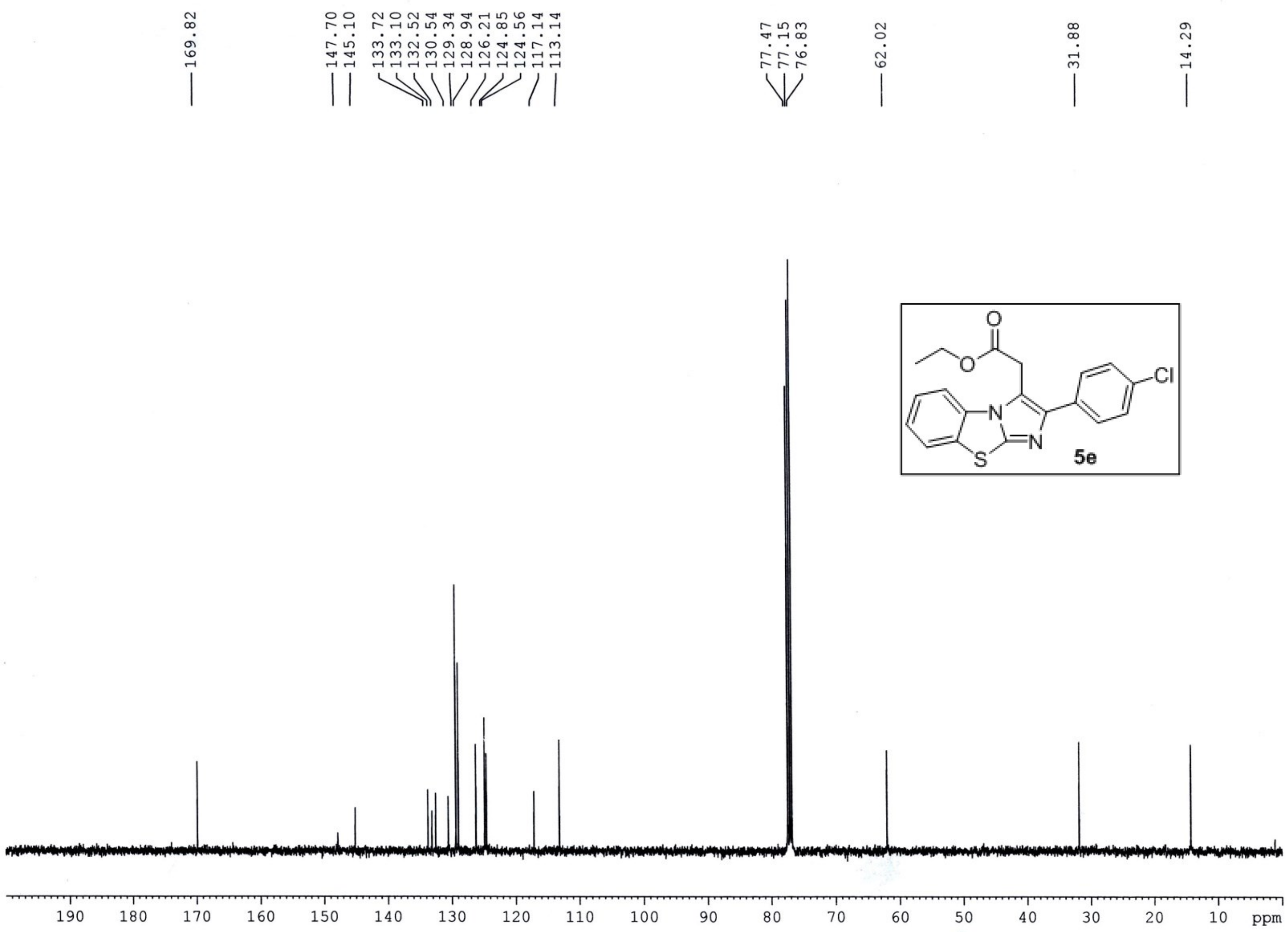
169.82
147.70
145.10
133.72
133.10
132.52
130.54
129.34
128.94
126.21
124.85
124.56
117.14
113.14
77.47
77.15
76.83
62.02
31.88
14.29
O
O
N
Cl
S
N
5e
190
180
170
160
150
140
130
120
110
100
90
80
70
60
50
40
30
20
10
ppm

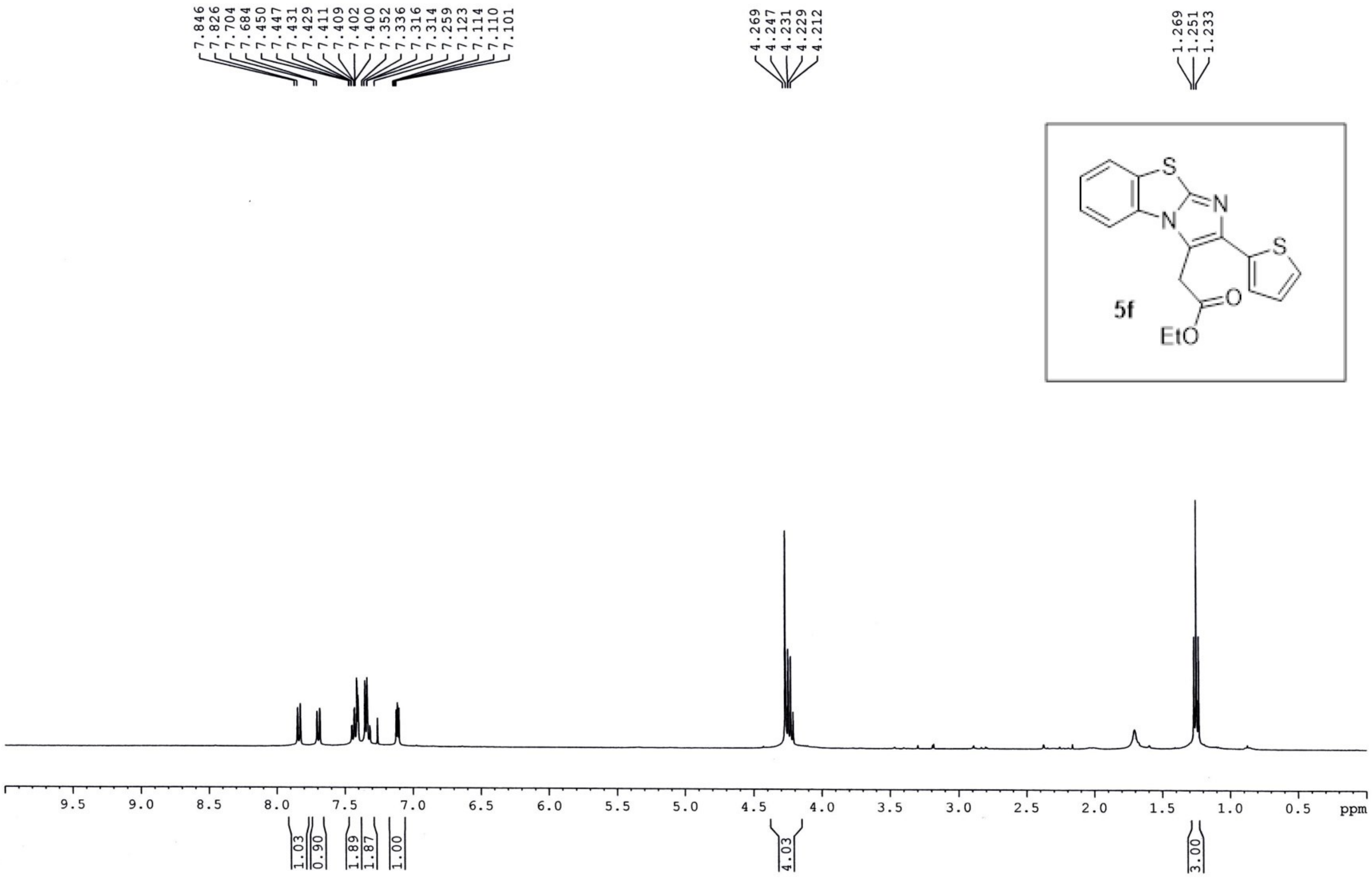
7.846
7.826
7.704
7.684
7.450
7.447
7.431
7.429
7.411
7.409
7.402
7.400
7.352
7.336
7.316
7.314
7.259
7.123
7.114
7.110
7.101
4.269
4.247
4.231
4.229
4.212
1.269
1.251
1.233
5f
EtO
9.5 9.0 8.5 8.0 7.5 7.0 6.5 6.0 5.5 5.0 4.5 4.0 3.5 3.0 2.5 2.0 1.5 1.0 0.5 ppm
1.03
0.90
1.89
1.87
1.00
4.03
3.00

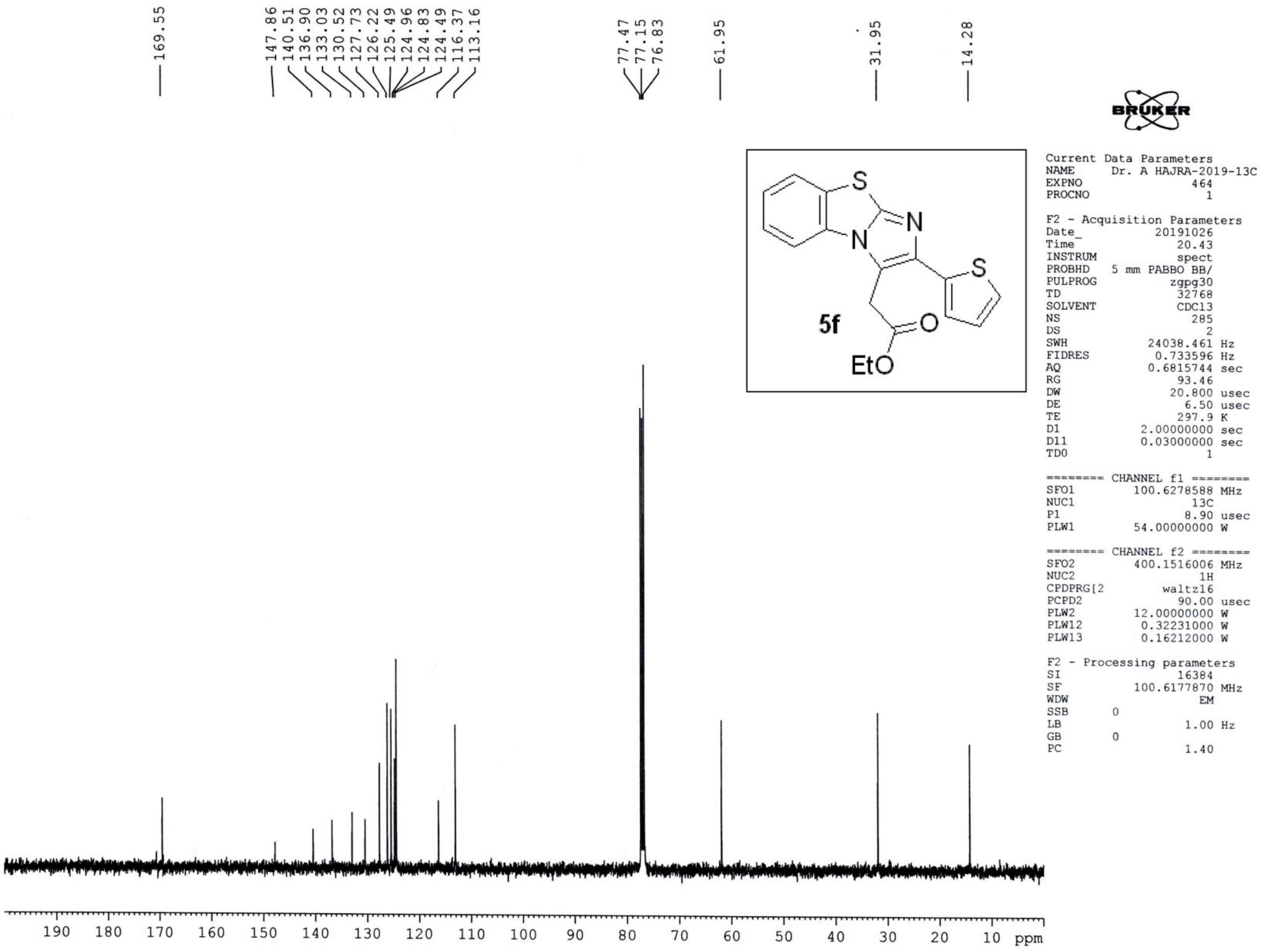
169.55
147.86
140.51
136.90
133.03
130.52
127.73
126.22
125.49
124.96
124.83
124.49
116.37
113.16
77.47
77.15
76.83
61.95
31.95
14.28
S
N
N
S
5f
O
EtO
190 180 170 160 150 140 130 120 110 100 90 80 70 60 50 40 30 20 10 ppm
BRUKER
Current Data Parameters
NAME Dr. A HAJRA-2019-13C
EXPNO 464
PROCNO 1
F2 - Acquisition Parameters
Date_ 20191026
Time_ 20.43
INSTRUM spect
PROBHD 5 mm PABBO BB/
PULPROG zgpg30
TD 32768
SOLVENT CDCl3
NS 285
DS 2
SWH 24038.461 Hz
FIDRES 0.733596 Hz
AQ 0.6815744 sec
RG 93.46
DW 20.800 usec
DE 6.50 usec
TE 297.9 K
D1 2.00000000 sec
D11 0.03000000 sec
TD0 1
======== CHANNEL f1 ========
SFO1 100.6278588 MHz
NUC1 13C
P1 8.90 usec
PLW1 54.00000000 W
======== CHANNEL f2 ========
SFO2 400.1516006 MHz
NUC2 1H
CPDPRG[2 waltz16
PCPD2 90.00 usec
PLW2 12.00000000 W
PLW12 0.32231000 W
PLW13 0.16212000 W
F2 - Processing parameters
SI 16384
SF 100.6177870 MHz
WDW EM
SSB 0
LB 1.00 Hz
GB 0
PC 1.40

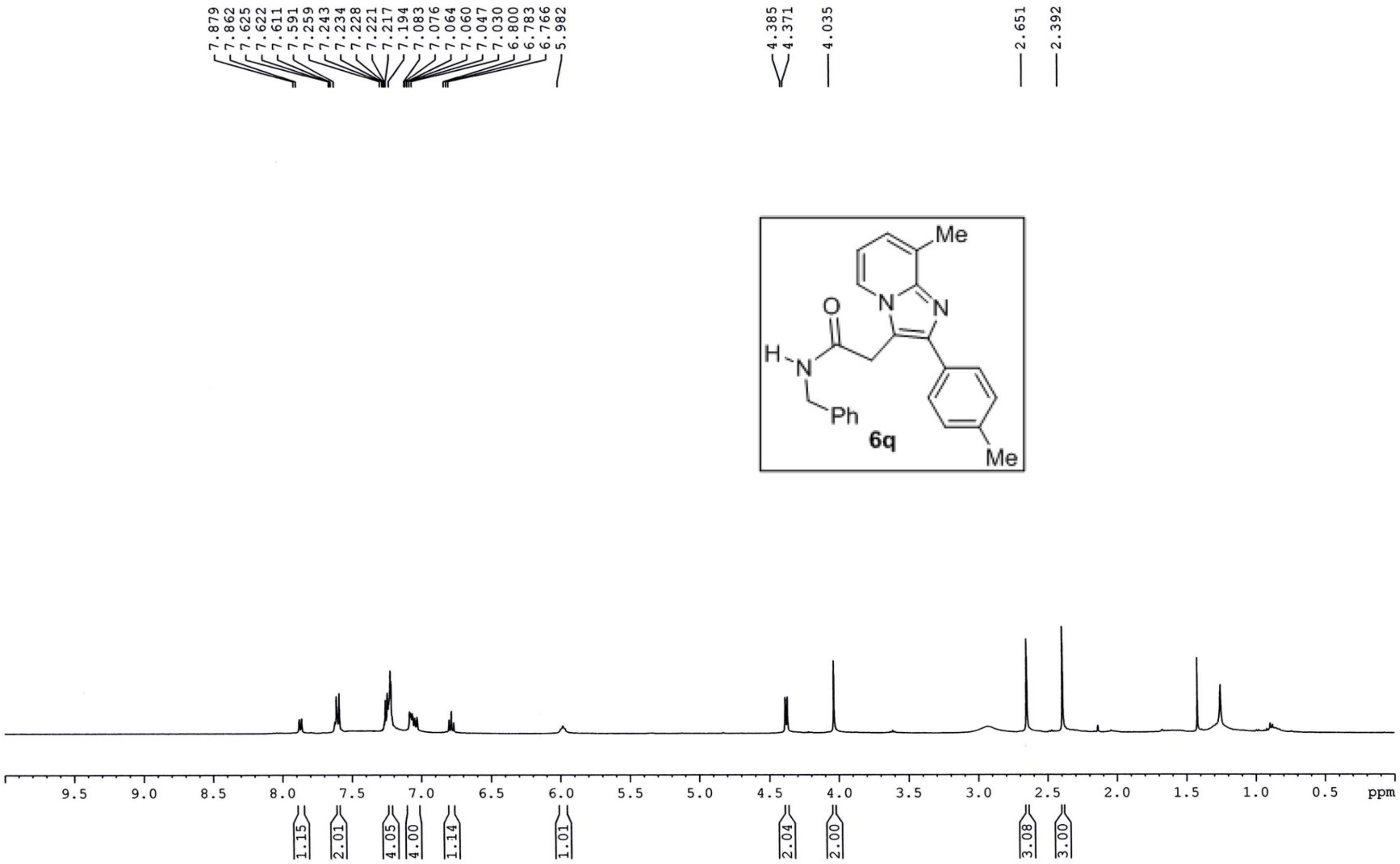
7.879
7.862
7.625
7.622
7.611
7.591
7.259
7.243
7.234
7.228
7.221
7.217
7.194
7.083
7.076
7.064
7.060
7.047
7.030
6.800
6.783
6.766
5.982
4.385
4.371
4.035
2.651
2.392
Me
O
N
N
H
N
Ph
6q
Me
9.5
9.0
8.5
8.0
7.5
7.0
6.5
6.0
5.5
5.0
4.5
4.0
3.5
3.0
2.5
2.0
1.5
1.0
0.5
ppm
1.15
2.01
4.05
4.00
1.14
1.01
2.04
2.00
3.08
3.00

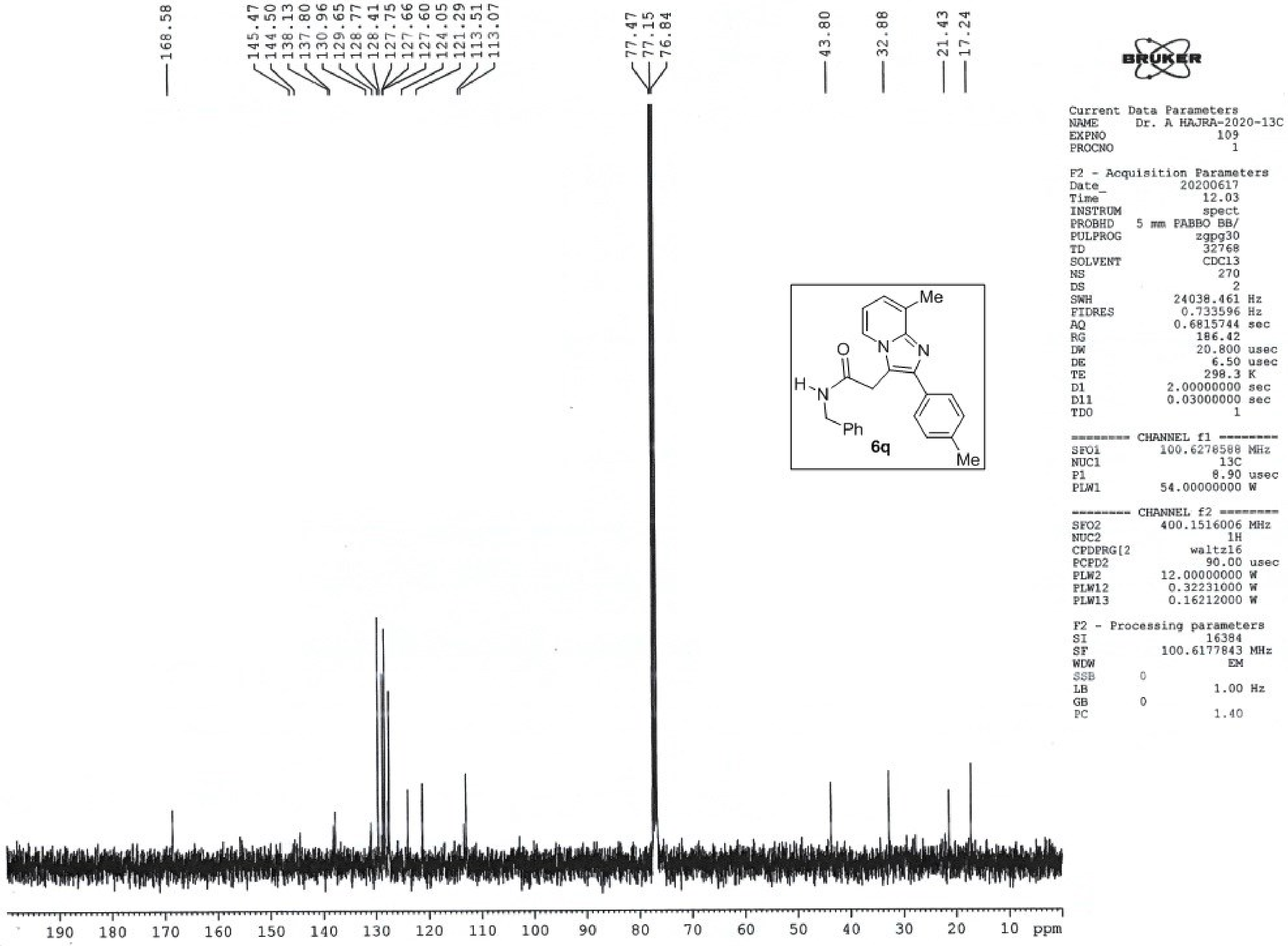

168.58
145.47
144.50
138.13
137.80
130.96
129.65
128.77
128.41
127.75
127.66
127.60
124.05
121.29
113.51
113.07
77.47
77.15
76.84
43.80
32.88
21.43
17.24
BRUKER
Current Data Parameters
NAME Dr. A HAJRA-2020-13C
EXPNO 109
PROCNO 1
F2 - Acquisition Parameters
Date_ 20200617
Time 12.03
INSTRUM spect
PROBHD 5 mm PABBO BB/
PULPROG zgpg30
TD 32768
SOLVENT CDCl3
NS 270
DS 2
SWH 24038.461 Hz
FIDRES 0.733596 Hz
AQ 0.6815744 sec
RG 186.42
DW 20.800 usec
DE 6.50 usec
TE 298.3 K
D1 2.00000000 sec
D11 0.03000000 sec
TD0 1
======== CHANNEL f1 ========
SFO1 100.6278588 MHz
NUC1 13C
P1 8.90 usec
PLW1 54.00000000 W
======== CHANNEL f2 ========
SFO2 400.1516006 MHz
NUC2 1H
CPDPRG[2 waltz16
PCPD2 90.00 usec
PLW2 12.00000000 W
PLW12 0.32231000 W
PLW13 0.16212000 W
F2 - Processing parameters
SI 16384
SF 100.6177843 MHz
WDW EM
SSB 0
LB 1.00 Hz
GB 0
PC 1.40
Me
O
N
N
H
N
Ph
6q
Me
190 180 170 160 150 140 130 120 110 100 90 80 70 60 50 40 30 20 10 ppm

1H of VBSB-22/REV-dwn

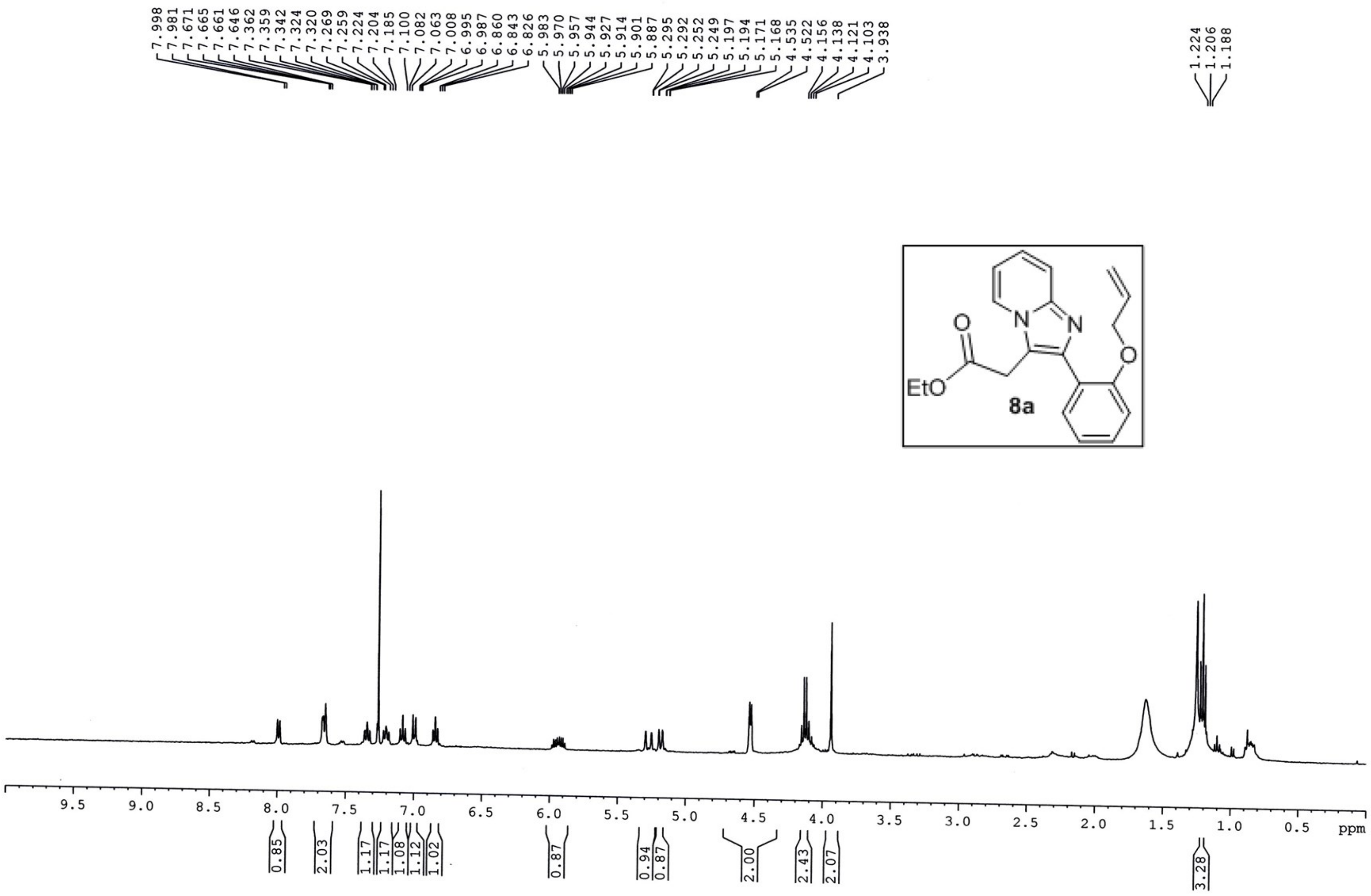

8a

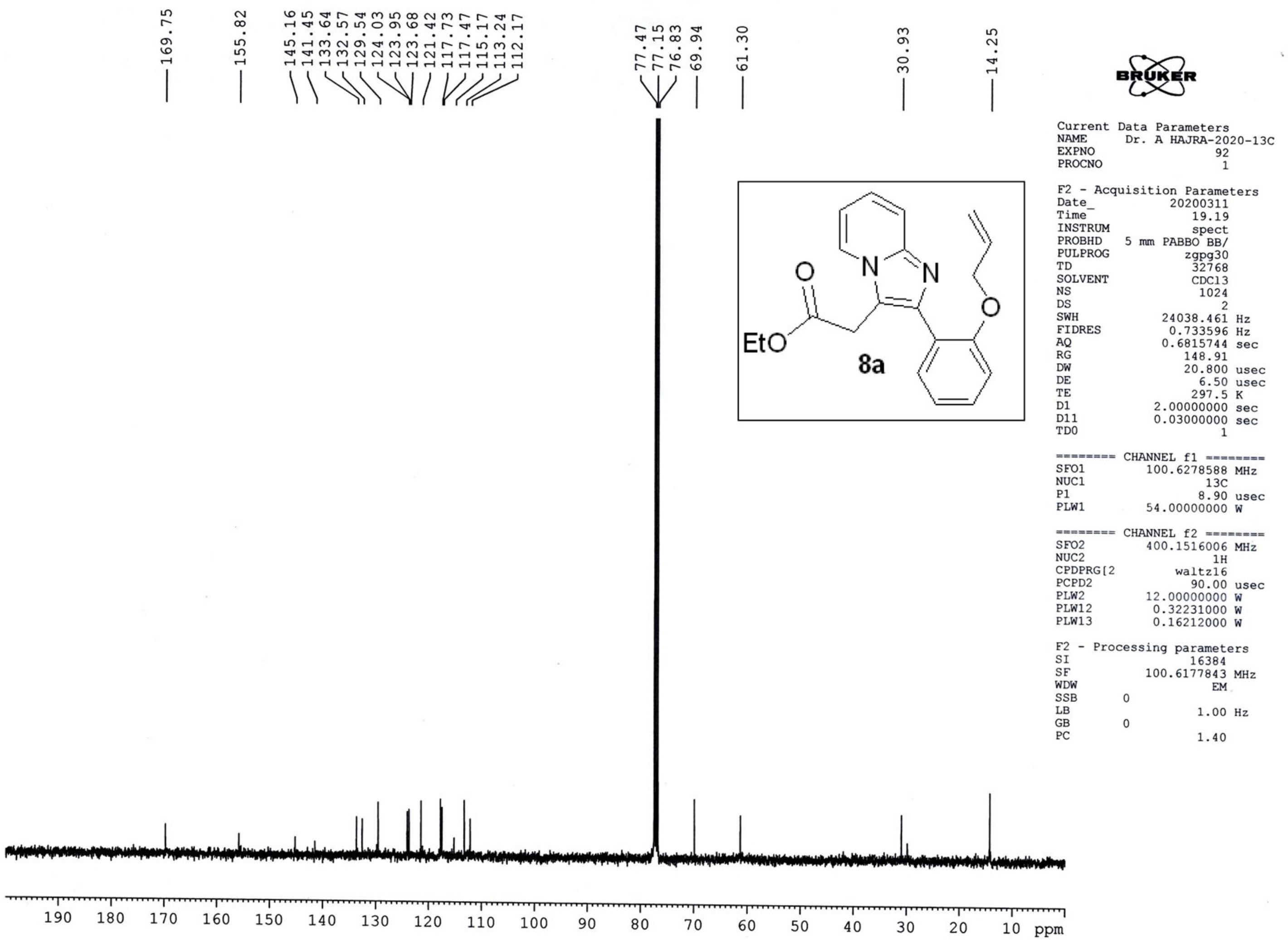
169.75
155.82
145.16
141.45
133.64
132.57
129.54
124.03
123.95
123.68
121.42
117.73
117.47
115.17
113.24
112.17
77.47
77.15
76.83
69.94
61.30
30.93
14.25
BRUKER
Current Data Parameters
NAME Dr. A HAJRA-2020-13C
EXPNO 92
PROCNO 1
F2 - Acquisition Parameters
Date_ 20200311
Time 19.19
INSTRUM spect
PROBHD 5 mm PABBO BB/
PULPROG zgpg30
TD 32768
SOLVENT CDCl3
NS 1024
DS 2
SWH 24038.461 Hz
FIDRES 0.733596 Hz
AQ 0.6815744 sec
RG 148.91
DW 20.800 usec
DE 6.50 usec
TE 297.5 K
D1 2.00000000 sec
D11 0.03000000 sec
TD0 1
======== CHANNEL f1 ========
SFO1 100.6278588 MHz
NUC1 13C
P1 8.90 usec
PLW1 54.00000000 W
======== CHANNEL f2 ========
SFO2 400.1516006 MHz
NUC2 1H
CPDPRG[2 waltz16
PCPD2 90.00 usec
PLW2 12.00000000 W
PLW12 0.32231000 W
PLW13 0.16212000 W
F2 - Processing parameters
SI 16384
SF 100.6177843 MHz
WDW EM
SSB 0
LB 1.00 Hz
GB 0
PC 1.40
EtO
O
N
N
O
8a
190 180 170 160 150 140 130 120 110 100 90 80 70 60 50 40 30 20 10 ppm
